# *Ortho*-Nitro Effect on the Diastereoselective Control in Sulfa-Staudinger and Staudinger Cycloadditions

**DOI:** 10.3390/molecules22050784

**Published:** 2017-05-12

**Authors:** Zhanhui Yang, Hassane Abdellaoui, Wei He, Jiaxi Xu

**Affiliations:** State Key Laboratory of Chemical Resource Engineering, Department of Organic Chemistry, Faculty of Science, Beijing University of Chemical Technology, Beijing 100029, China; zhyang_2008a@163.com (Z.Y.); hasabdel89@hotmail.fr (H.A.); bucthewei@163.com (W.H.)

**Keywords:** nitro effect, diastereoselectivity, inductive effect, sulfa-Staudinger cycloaddition, Staudinger cycloaddition, stereochemistry

## Abstract

The *ortho*-nitro effect was discovered in sulfa-Staudinger cycloadditions of ethoxycarbonylsulfene with linear imines. When an *ortho*-nitro group is present at the *C*-aryl substituents of linear imines, the sulfa-Staudinger cycloadditions deliver *cis*-β-sultams in considerable amounts, together with the predominant *trans*-β-sultams. In other cases, the above sulfa-Staudinger cycloadditions give rise to *trans*-β-sultams exclusively. Further mechanistic rationalization discloses that the *ortho*-nitro effect is attributed to its strong electron-withdrawing inductive effect. Similarly, the *ortho*-nitro effect also exists in Staudinger cycloadditions of ethoxycarbonyl ketene with the imines. The current research provides further insights into the diastereoselective control in sulfa-Staudinger and Staudinger cycloadditions.

## 1. Introduction

Recently, β-sultams have received much attention in both synthetic and medicinal chemistry [1,2,3,4,5,6,7,8], mainly because of their outstanding biological activities as, for example, antibiotics and enzyme inhibitors [9,10,11,12,13]. As a result, many synthetic methods to β-sultams have been developed [14,15,16,17]. Among them, the most promising is the sulfa-Staudinger cycloaddition [18,19,20,21,22,23,24], which is referred to as the [2^s^ + 2^i^] annulation between sulfenes (or their equivalents) with imines, because of the rich and diverse sources of the starting materials.

**Scheme 1:**
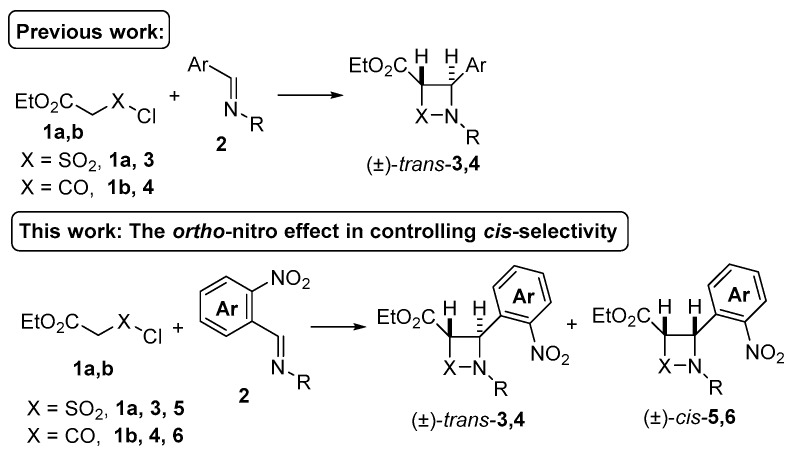
Diastereoselectivities in sulfa-Staudinger and Staudinger cycloadditions.

In our previous work, we successfully disclosed the stepwise mechanism of the sulfa-Staudinger cycloaddition [25,26], and the substituent-controlled annuloselectivity [27,28] and diastereoselectivity [29] in the cycloaddition. The previous studies on the diastereoselectivity revealed that the [2^s^ + 2^i^] annulation of the active ethoxycarbonylmethanesulfonyl chloride (**1a**) and ethyl malonyl chloride (**1b**) with various linear imines **2** gave *trans*-β-sultams **3** or *trans*-β-lactams **4** exclusively, regardless of the imine substituents (Scheme 1) [27,28,29,30,31]. However, in our continuing studies, we further found that when *C*-2-nitroaryl imines **2** were applied in the reactions, besides *trans*-β-sultams **3**, *cis*-β-sultams **5** were generated in considerable amounts (Scheme 1, X = SO_2_). This unexpected phenomenon is, herein, considered as the *ortho*-nitro effect. Moreover, such *ortho*-nitro effect also was observed in the Staudinger ketene-imine cycloadditions (Scheme 1, X = CO).

Previous work on the nitro effect mainly focused on the reactivity of certain compounds [32,33,34], mainly because of the electronic properties of the nitro group. In addition, the shielding effect of the nitro group was also reported [35]. However, the nitro effect on the diastereoselectivity has not been reported until now. Herein, we rationalize how the *ortho*-nitro effect works to generate *cis*-β-sultams and *cis*-β-lactams in the reactions of *C*-2-nitroaryl imines with sulfenes and ketenes, respectively, and hope that the current research could help to further understand the diastereoselective control in the sulfa-Staudinger and Staudinger cycloadditions.

## 2. Results and Discussion

### 2.1. Experimental Studies on the Ortho-Nitro Effect in Controlling Cis-Selectivity of Sulfa-Staudinger Cycloadditions

Previously, the *ortho*-nitro effect was seldom observed in our studies on the substituent-controlled annuloselectivity [27,28]. The reaction between sulfonyl chloride **1a** and *C*-2-nitrophenylimine **2a** gave rise to a mixture of *trans*- and *cis*-β-sultams **3a** and **5a** in 66% total isolated yield, with an 82:18 *trans/cis* ratio (Table 1, entry **1**) [36]. However, *C*-3- and 4-nitrophenyl imines **2b** and **2c** afforded the corresponding *trans*-β-sultams **3b** and **3c** exclusively in 79% and 41% isolated yields, respectively, in the reactions with the same sulfonyl chloride **1a** (Table 1, entries 2 and 3). The sulfa-Staudinger reactions of other *C*-2-nitrophenyl imines **2d**–**f** with sulfonyl chloride **1a** produced the corresponding *cis*-β-sultams **5d**–**f** as well, with *trans/cis* ratios 86:14, 88:12, and 97:3, respectively (Table 1, entries 4–6). The increasing trend of the *trans/cis* ratios from entry 4 to entry 6 is in good accordance to our previous diastereoselective guidelines, that is, the bulky *N*-substituents of imines favor the *trans*-selectivity [29]. These results indicate that the generation of *cis*-β-sultams in the sulfa-Staudinger reactions of *C*-2-nitrophenyl imines with ethoxycarbonylmethanesulfonyl chloride (**1a**) is a general and regular phenomenon. In these reactions, the *ortho*-nitro group on the *C*-aryl group plays an important role in formation of *cis*-β-sultams. Experimental results from entries 7–9 in Table 1 present a decreasing trend of the *trans/cis* ratios without obvious difference between *C*-2-nitrophenylimine **2a** and *C*-3- and 4-nitrophenylimines **2b** and **2c**, revealing that the *ortho*-nitro effect does not exist in the sulfa-Staudinger cycloadditions involving other alkanesulfonyl chlorides, such as phenylmethanesulfonyl chloride (**1c**). In the presence of weak bases such as pyridine, the *ortho*-nitro effect still exists (Table 1, entries 10–12). In addition, [2 + 2 + 2] cycloadducts **7** were observed in low yields when excessive *N*-methyl imines were employed (Table 1, entries 2 and 3), while [4 + 2] annuladducts **8**, generated from enolate-sulfene and imines, were not observed in any cases. It is noteworthy that the *trans/ci*s ratios do not change upon storage for several months or treatment with imines for several days, indicating that the reactions are under kinetic control.

To distinguish whether other *ortho*-substituents affect the diastereoselectivity, other imines derived from *ortho*-substituted aromatic aldehydes were also tested in the reactions of sulfonyl chloride **1a**. As shown in entries 2–9 in Table 2, regardless of the *ortho*-substituents of imines **2g**–**o**, all the sulfa-Staudinger annuladducts *trans*-**3g**–**o** were generated in excellent diastereoselectivities, with the *trans/cis* ratios varying from 97:3 to 100:0. All the above results indicate that only when a nitro group is present at the *ortho*-position in the *C*-aryl groups of imines **2** would *cis*-β-sultams be generated in considerable amounts. The other 2-substituents at the aryl groups of imines scarcely affect the diastereoselectivities, as demonstrated by reactions of imines **2g**–**o** (Table 2, entries 2–10). However, the presence of an additional electron-withdrawing group, such as 4-NO_2_ and 5-MeO (according to Hammett constants, *meta*-MeO is an electron-withdrawing group), at the 2-nitroaryl promoted the formation of the corresponding *cis*-products, as shown in entries 11 and 12 of Table 2.

### 2.2. Rationale on the Ortho-Nitro Effect in Controlling Cis-Selectivity of Sulfa-Staudinger Cycloadditions

**Scheme 2:**
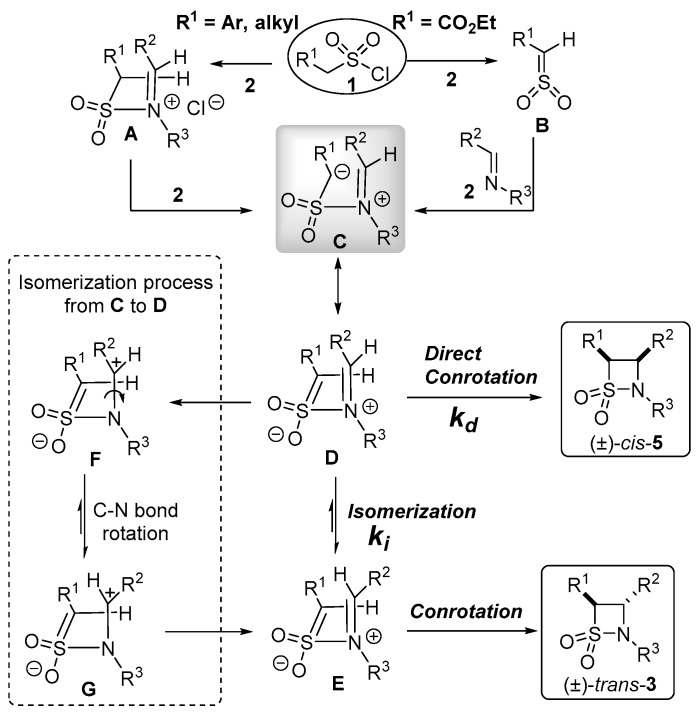
Mechanism of sulfa-Staudinger cycloadditions.

One question of significant concern is how the *ortho*-nitro effect controls the formation of *cis*-β-sultams together with *trans*-β-sultams. Before answering this question, the mechanism in sulfa-Staudinger reactions should first be introduced. In our previous report [25,26,27,28,29], the mechanism for the formation of β-sultam rings has been rationalized. Under thermal conditions, the imines exist in *E*-configuration, as demonstrated by our previous work [37]. As depicted in Scheme 2, arylmethane- or alkane-sulfonyl chlorides **1** (R^1^ = aryl or alkyl) first sulfonylate imines **2** to afford intermediates **A**, which further generate intermediates **C** or their resonation forms **D** through elimination of a proton by a weak base or imines. Alternatively, sulfenes **B**, generated from electron-deficient sulfonyl chlorides **1** (R^1^ = CO_2_Et) such as ethoxycarbonylmethanesulfonyl chloride (**1a**) in the presence of weakly basic imines **2** [27,28,29], react with nucleophilic imines **2** to deliver intermediates **C** or their resonation forms **D**. Direct conrotation of **D** gives rise to *cis*-β-sultams **5**, while isomerization of **D** into **E**, followed by conrotation of **E** produces *trans*-β-sultams **3**. The isomerization step is realized by a C–N bond rotation through **G** and **F** as possible intermediates. The diastereoselectivity (*cis*/*trans*-selectivity) is virtually the competitive results between the direct conrotation and isomerization of zwitterionic 2,3-thiaza-1,3-butadiene-type intermediates **D**. The electron-withdrawing substituents of sulfenes decrease the rate (*k*_d_) of the direct conrotatory ring closure of zwitterionic intermediates **D**, resulting in favorable formation of *trans*-β-sultams **3**, while the electron-withdrawing *C*-substituents of imines increase the rate of the direct conrotatory ring closure, leading to predominant formation of *cis*-β-sultams **5**.

**Scheme 3:**
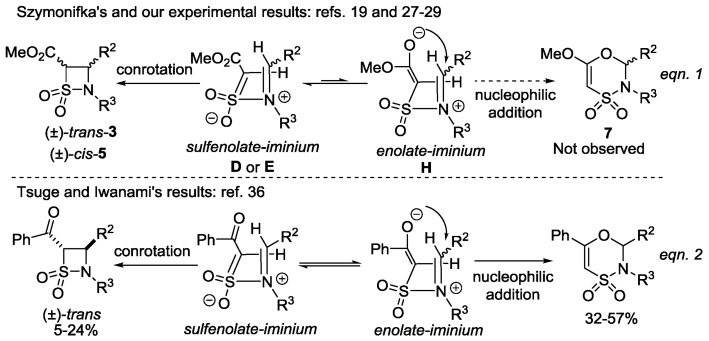
Sulfenolate-iminium vs. enolate-iminium intermediates in the sulfa-Staudinger cycloadditions involving acylsulfenes.

Szymonifka and Heck also studied the reactions of methoxycarbonylsufene with imines, and *trans*-β-sultams were formed exclusively [19]. They proposed zwitterionic enolate-iminium intermediates H (eqn. 1, Scheme 3). As demonstrated by Tsuge and Iwanami’s results [38], if enolate-iminium intermediates **H** are formed, the more favorable six-membered products 2,3-dihydro-1,4,3-oxathiazine 4,4-dioxides **8** should be generated (eqn. 2, Scheme 3). However, the fact is that 2,3-dihydro-1,4,3-oxathiazine 4,4-dioxide products **8** were not observed in either Szymonifka’s [19] or our studies (eqn. 1, Scheme 3) [27,28,29]. Therefore, it is not enolate-iminium intermediates **H** but sulfenolate-iminium intermediates **D** or **E** that partake in the ring closure step in the ester-stabilized systems. The results are in accordance with the general observation that acylacetate esters are predominantly enolized rather than malonate diesters.

The reaction of sulfonyl chloride **1a** and *C*-2-nitrophenylimine **2a** shows higher *cis*-selectivity (*trans/cis* ratio of 82:18) than those in reactions of **1a** with *C*-3- and 4-nitrophenyl imines **2b** and **2c** (Table 1, entries 1–3). Additionally, all reactions of *C*-2-nitrophenylimines **2d**–**f** increase *cis*-selectivities compared with reactions of other *C*-2-substituted phenyl imines **2g**–**o** (Table 1, entries 4–6 vs. Table 2, entries 2–9). Thus, in reactions of *C-*2-nitrophenylimines, the formation of *cis*-β-sultams **5** can be attributed to the strong electron-withdrawing inductive effect of the *ortho*-nitro group rather than steric hindrance. Both *C*-3- and 4-nitrophenyl imines **2b** and **2c** afforded the corresponding *trans*-β-sultams **3b** and **3c** exclusively in the reactions with the same sulfonyl chloride **1a** (Table 1, entries 2 and 3) due to longer distances from the nitro group to the carbon atom of the iminium than that in the *ortho*-nitro substrate, weakening the electron-withdrawing inductive effect because the inductive effect exerts electron-withdrawing influence through the carbon chain and decreases along with distance. The results also reveal that the conjugation effect of the nitro group does not play an important role in controlling the *cis*-selectivity because the conjugation effect is not closely related to the distance.

To further verify the influence of the inductive effect of the nitro group on the diastereoselectivity, imine **2p** with *C*-2,4-dinitrophenyl group and imine **2q** with *C*-5-methoxy-2-nitrophenyl group were reacted with sulfonyl chloride **1a**; the corresponding *cis*-β-sultams **5p** and **5q** increased obviously compared with that from *C*-2-nitrophenylimine **2a**. The results indicate that the *ortho*-nitro effect on the *cis*-selectivity is its strong electron-withdrawing inductive effect.

On the other hand, the increase of *cis*-β-sultams **5** may also be obtained by decelerating the isomerization of **D** to **E** (Scheme 2). One significantly important factor in affecting the isomerization is the steric hindrance of R^2^. The isomerization is realized by the C–N bond rotation (from **F** to **G**). If R^2^ is sterically too large, the C–N conrotation will not overcome the steric hindrance between R^1^ and R^2^, and the isomerization will be decelerated or prevented, promoting the generation of *cis*-β-sultams **5**, even as sole products in some cases. This phenomenon was observed in the reactions of phenylmethanesulfonyl chlorides with (*E*)-1-(anthracen-9-yl)-*N*-benzylmethanimine, which bore a sterically very large 9-anthracenyl group (R^2^), and only *cis*-β-sultam was obtained in our previous work [29]. Imines **2g**−**o** in entries 2–9 in Table 2 have sterically approximate or larger groups than the nitro group at their 2-positions. However, *cis*-β-sultams **5** were generated in trace amounts. The comparison indicates that it is not the steric interaction between the *ortho*-nitroaryl group (R^2^) and the ethoxycarbonyl group that leads to *cis*-β-sultams **5**. Actually, as illustrated in Scheme 2, during the isomerization of **D** to **E**, the C–N bond rotation in intermediates **F** could occur clockwise or anticlockwise, following the direction with the least steric hindrance. In other words, the steric interaction between the *ortho*-substituents and CO_2_Et cannot decelerate the isomerization, and this is the reason why the reactions of a variety of 2-substituted imines with **1a** predominantly give *trans*-β-sultams **3** (Table 2, entries 2–10). The steric interaction between the *ortho*-nitroaryl group (R^1^) and the ethoxycarbonyl group (R^2^) is not the key factor that leads to the *ortho*-nitro effect.

**Figure 1:**
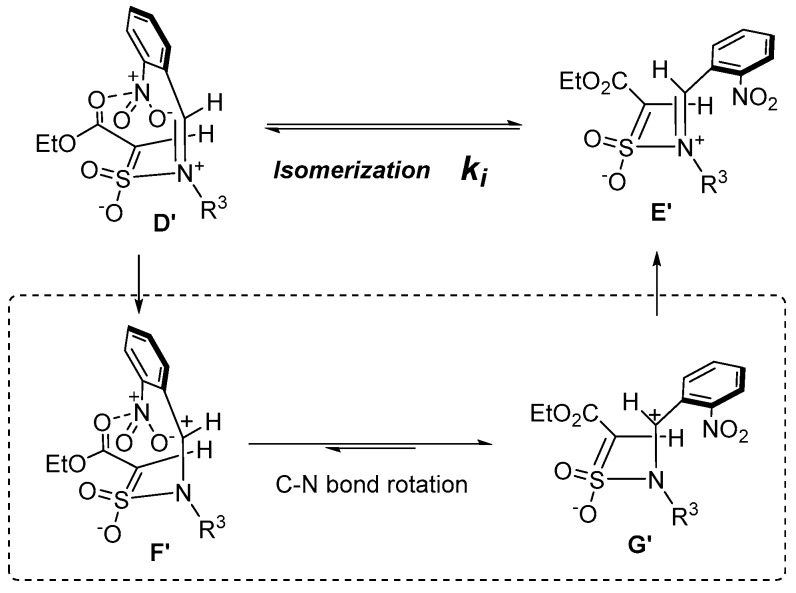
*Ortho*-nitro effect by possible electrostatic interaction.

Additionally, the electrostatic interaction between the *ortho*-nitro group and the ethoxycarbonyl group is possibly another factor for the *ortho*-nitro effect as shown in (Figure 1); the positively charged nitrogen atom of the nitro group interacts with the partially negatively charged oxygen atom of the C=O bond in the ethoxycarbonyl group, to some extent stabilizing the intermediates **D′** and decelerating the isomerization of **D′** to **E′**. As a consequence, *cis*-β-sultams **5** were generated. Thus, the electrostatic interaction is another rationale for the *ortho*-nitro effect in the current sulfa-Staudinger cycloadditions. Such an electrostatic interaction does not exist between the 3-nitro or 4-nitro group and the ethoxycarbonyl group possibly because of the far distance between them. Consequently, the corresponding *cis*-β-sultams **5b** or **5c** were not generated (Table 1, entries 2 and 3). However, on the basis of the results from imines **2p** and **2q**, the electrostatic interaction between the *ortho*-nitro group and the ethoxycarbonyl group is not a major factor for the *ortho*-nitro effect even if it exists.

### 2.3. Ortho-Nitro Effect in the Staudinger Ketene-Imine Cycloadditions

To examine the generality of the *ortho*-nitro effect, the Staudinger cycloadditions of ethyl malonyl chloride (**1b**) and some *C*-nitroaryl imines **2** were conducted. The *ortho*-nitro effect was also observed in Staudinger ketene-imine cycloadditions. The results were summarized in Table 3. Ethoxycarbonylketene, generated from ethyl malonyl choride (**1b**) in the presence of 2-chloropyridine, smoothly reacted with *C*-2-nitrophenyl imine **2d**, giving rise to both *trans*-**4d** and *cis*-**6d** with a *trans/cis* ratio as 83:17 (Table 3, entry 1) [36]. However, the reactions of *C*-3-nitrophenyl and *C*-4-nitrophenyl imines **2p** and **2q** delivered *trans*-β-lactams **4p** and **4q** exclusively in 54%, and 58% yields, respectively (Table 3, entries 2 and 3). The *ortho*-nitro effect in the Staudinger ketene-imine cycloaddition is a common phenomenon, and is affected by the *N*-substituents of *C*-2-nitroaryl imines. For example, the reactions of *N*-methyl imine **2a** and *N*-benzyl imine **2e** afforded the *trans/cis* ratios in 85:15 and 81:19, respectively (Table 3, entries 4 and 5). However, the reaction of *N-tert*-butyl imine **2f** produced more *cis*-β-lactam **6f** with a 64:36 *trans/cis* ratio (Table 3, entry 6) possibly due to the Thorpe-Ingold effect [39,40]. Correspondingly, imine **2r** reacted with acyl chloride **1b**, only giving *trans*-β-lactam **4r** in 74% yield (Table 3, entry 7).

**Figure 2:**
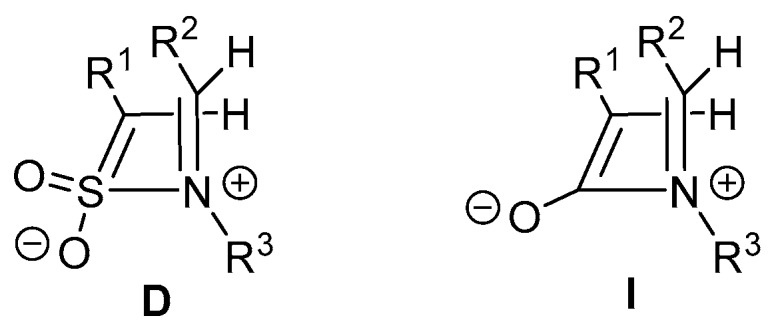
Key zwitterionic intermediates involved in sulfa-Staudinger and Staudinger cycloadditions.

The *ortho*-nitro effect exists in not only sulfa-Staudinger sulfene-imine cycloadditions, but also Staudinger ketene-imine cycloadditions, to some extent indicating that the structures of the two zwitterionic intermediates **D** and **I** involved in the above two types of cycloadditions are similar [37,41,42,43,44,45,46,47,48] (Figure 2). The nature of the *ortho*-nitro effect in Staudinger cycloadditions is the same as that in sulfa-Staudinger cycloadditions.

## 3. Materials and Methods

### 3.1. Materials and Instruments

Tetrahydrofuran was dried by refluxing over sodium with diphenyl ketone as an indicator. Melting points were obtained on a Yanaco MP-500 melting point apparatus (Yanaco Ltd., Osaka, Japan) and are uncorrected. ^1^H- and ^13^C-NMR spectra were recorded on a Bruker AM 400 MHz spectrometer (Bruker Company, Billerica, MA, USA) in CDCl_3_ with TMS as an internal standard and the chemical shifts (δ) are reported in parts per million (ppm). The IR spectra (KBr pellets) were taken on a Nicolet FTIR 920 spectrometer (Thermo Nicolet Corporation, Madison, WI, USA). HRMS measurements were carried out on an Agilent LC/MSD TOF mass spectrometer (Agilent, Santa Clara, CA, USA). TLC separations were performed on silica gel GF254 plates (Qingdao Ocean Chemical Industry, Qingdao, China), and the plates were visualized with UV light. Column chromatography was performed on silica gel zcx II (200–300 mesh) (Qingdao Ocean Chemical Industry, Qingdao, China) with petroleum-ether (PE) and ethyl-acetate (EA) (Beijing Chemical Reagent Company, Beijing, China) as the eluent.

### 3.2. General Procedure for the Sulfa-Staudinger Cycloadditions of Sulfonyl Chlorides ***1a**,**c*** and Imines ***2***

To a solution of imine **2** (1 mmol) in dry THF (2 mL) was added a solution of sulfonyl chloride **1a** (or **1c**) (0.5 mmol) in dry THF (0.5 mL) for 0.5 min at room temperature. The mixture was then allowed to stand at room temperature for 24 h, followed by dilution with ether (10 mL), washing with brine (10 mL), and drying over Na_2_SO_4_. Filtrating off the desiccant and removing the solvents under vacuum gave rise to a residue, which subsequently was subjected to column chromatography on silica gel with a mixture of petroleum ether (PE, 30–60 °C) and ethyl acetate (EA) as eluent to give the desired β-sultam products.

For the reactions in entries 10–12 in Table 1, the following procedure was used. To a solution of imine **2a** or **2b** or **2c** (22 mg, 0.125 mmol) and pyridine (12 mg, 0.15 mmol) in dry THF (0.5 mL) was added a solution of sulfonyl chloride **1a** (28 mg, 0.15 mmol) in dry THF (0.25 mL) for 0.5 min at room temperature. Then the mixture was allowed to stand at room temperature for 24 h, followed by dilution with ether (5 mL), and washing with brine (5 mL). Removing the solvents under vacuum gave rise to a residue, which subsequently was subjected to NMR analysis to determine the *trans/cis* ratios and yields.

*Ethyl trans-2-methyl-3-(2-nitrophenyl)-1,2-thiazetidine-4-carboxylate 1,1-dioxide* (**3a**) [27,28]: Compounds *trans*-**3a** and *cis*-**5a** were isolated as a mixture, with a *trans/cis* ratio as 82:18. Yellow oil. Total yield 66% (103 mg). ^1^H-NMR (400 MHz, CDCl_3_): 8.11–8.08 (m, 1H), 7.83–7.79 (m, 1H), 7.63–7.59 (m, 1H), 5.02 (d, *J* = 5.0 Hz, 1H), 4.87 (d, *J* = 5.0 Hz, 1H), 4.40 (q, *J* = 7.2 Hz, 2H), 2.90 (s, 3H), 1.37 (t, *J* = 7.2 Hz, 3H). ^13^C-NMR (400 MHz, CDCl_3_): 162.3, 148.3, 134.7, 131.7, 130.1, 129.6, 128.3, 125.4, 80.8, 63.3, 51.6, 31.8, 14.0.

*Ethyl cis-2-methyl-3-(2-nitrophenyl)-1,2-thiazetidine-4-carboxylate 1,1-dioxide* (**5a**) [27,28]: Yellowish oil. ^1^H-NMR (400 MHz, CDCl_3_): 8.23 (dd, *J* = 8.2, 1.1 Hz, 1H), 8.02 (d, *J* = 7.4 Hz, 1 H), 7.83–7.79 (m, 1H), 7.63–7.59 (m, 1H), 5.67 (d, *J* = 8.4 Hz, 1H), 4.94 (d, *J* = 8.4 Hz, 1H), 4.08–3.97 (m, 2H), 2.87 (s, 3H), 1.09 (t, *J* = 7.2 Hz, 3H). ^13^C-NMR (400 MHz, CDCl_3_): 161.5, 147.7, 134.4, 133.4, 129.65, 129.64, 125.5, 76.9, 62.5, 53.1, 29.7, 13.7.

*Ethyl trans-2-methyl-3-(3-nitrophenyl)-1,2-thiazetidine-4-carboxylate 1,1-dioxide* (**3b**): Known compound [27,28]. Colorless crystals. M.p.: 105–107 °C. Yield 79% (124 mg). ^1^H-NMR (400 MHz, CDCl_3_): 8.37 (t, *J* = 1.8 Hz, 1H), 8.26 (ddd, *J* = 8.2, 2.1, 0.9 Hz, 1H), 7.89 (d, *J* = 7.7 Hz, 1H), 7.65 (t, *J* = 8.0 Hz, 1H), 4.88 (d, *J* = 6.6 Hz, 1H), 4.61 (d, *J* = 6.6 Hz, 1H), 4.44−4.37 (m, 1H), 4.37−4.28 (m, 1H), 2.81 (s, 3H), 1.36 (t, *J* = 7.2 Hz, 3H). ^13^C NMR (400 MHz, CDCl_3_): 161.8, 148.8, 137.6, 132.6, 130.5, 124.3, 121.7, 79.2, 63.5, 54.5, 31.4, 14.0.

*Ethyl trans-2-methyl-3-(4-nitrophenyl)-1,2-thiazetidine-4-carboxylate 1,1-dioxide* (**3c**): Known compound. Colorless oil. Yield 41% (69 mg). ^1^H-NMR (400 MHz, CDCl_3_): 8.29 (d, *J* = 8.7 Hz, 2H), 7.72 (d, *J* = 8.7 Hz, 2H), 4.85 (d, *J* = 6.6 Hz, 1H), 4.60 (d, *J* = 6.6 Hz, 1H), 4.40 (dq, *J* = 10.8, 7.1 Hz, 1H), 4.33 (dq, *J* = 10.8, 7.1 Hz, 1H), 2.80 (s, 3H), 1.36 (t, *J* = 7.1 Hz, 3H). ^13^C-NMR (400 MHz, CDCl_3_): 161.8, 148.5, 142.3, 127.6, 124.5, 79.2, 63.5, 54.5, 31.4, 14.0.

*Ethyl trans-3-(2-nitrophenyl)-2-propyl-1,2-thiazetidine-4-carboxylate 1,1-dioxide* (**3d**): Compounds *trans*-**3d** and *cis*-**5d** were isolated as a mixture, with a *trans/cis* ratio as 86:14, from a 1mmol-scale reaction. Yellow oil, total yield 66% (225 mg). ^1^H-NMR (400 MHz, CDCl_3_): 8.15 (d, *J* = 7.8 Hz, 1H), 8.06 (dd, *J* = 8.2, 0.9 Hz, 1H), 7.78 (t, *J* = 7.8 Hz, 1H), 7.61–7.55 (m, 1H), 5.07 (d, *J* = 4.8 Hz, 1H), 4.81 (d, *J* = 4.8 Hz, 1H), 4.40 (q, *J* = 7.1 Hz, 2H), 3.44–3.31 (m, 1H), 3.01–2.90 (m, 1H), 1.68–1.57 (m, 2H), 1.36 (t, *J* = 7.1 Hz, 3H), 0.98 (t, *J* = 7.4 Hz, 3H). ^13^C-NMR (101 MHz, CDCl_3_): 162.3, 134.4, 132.4, 130.0, 128.5, 125.2, 80.4, 63.3, 50.1, 49.1, 21.6, 14.0, 11.6. IR (KBr): ν 2968, 2938, 2872, 1745, 1533, 1367, 1338, 1258, 1184, 1001, 1011, 861, 793, 518 cm^−1^. HRMS (ESI) calcd for C_14_H_18_N_2_NaO_6_S [M + Na]^+^: 365.0778; found: 365.0771.

*Ethyl cis-3-(2-nitrophenyl)-2-propyl-1,2-thiazetidine-4-carboxylate 1,1-dioxide* (**5d**): Yellow oil. ^1^H-NMR (400 MHz, CDCl_3_): 8.20 (d, *J* = 8.2 Hz, 1H), 8.08 (d, *J* = 7.7 Hz, 1H), 7.78 (t, *J* = 7.7 Hz, 1H), 7.62–7.54 (m, 1H), 5.62 (d, *J* = 8.5 Hz, 1H), 4.95 (d, *J* = 8.5 Hz, 1H), 4.10–4.02 (m, 1H), 4.02–3.94 (m, 1H), 3.43–3.32 (m, 1H), 2.84–2.74 (m, 1H), 1.82–1.71 (m, 2H), 1.09 (t, *J* = 7.2 Hz, 3H), 1.05 (t, *J* = 7.4 Hz, 3H). ^13^C-NMR (101 MHz, CDCl_3_): 162.4, 148.3, 134.2, 129.9, 129.6, 125.4, 76.3, 62.5, 51.6, 49.5, 21.8, 13.7, 11.7. IR (KBr): ν 2968, 2938, 2872, 1745, 1533, 1367, 1338, 1258, 1184, 1001, 1011, 861, 793, 518 cm^−1^. HRMS (ESI) calcd for C_14_H_18_N_2_NaO_6_S [M + Na]^+^: 365.0778; found: 365.0771.

*Ethyl trans-2-benzyl-3-(2-nitrophenyl)-1,2-thiazetidine-4-carboxylate 1,1-dioxide* (**3e**): Compounds *trans*-**3e** and *cis*-**5e** were isolated as a mixture, with a *trans/cis* ratio as 88:12, from a 1-mmol-scale reaction. Yellow oil, total yield 71% (276 mg). ^1^H-NMR (400 MHz, CDCl_3_): 8.04 (d, *J* = 7.8 Hz, 1H), 7.96 (d, *J* = 8.0 Hz, 1H), 7.67 (t, *J* = 7.6 Hz, 1H), 7.56–7.46 (m, 1H), 7.32–7.22 (m, 5H), 5.13 (d, *J* = 4.8 Hz, 1H), 4.84 (d, *J* = 4.8 Hz, 1H), 4.53 (d, *J* = 14.2 Hz, 1H), 4.39 (q, *J* = 7.1 Hz, 2H), 4.25 (d, *J* = 14.2 Hz, 1H), 1.36 (t, *J* = 7.1 Hz, 3H). ^13^C-NMR (101 MHz, CDCl_3_): 162.2, 148.0, 134.2, 133.4, 131.9, 129.8, 128.9, 128.7, 128.7, 128.3, 125.0, 80.4, 63.3, 50.7, 49.8, 14.0. IR (KBr): ν 2987, 2920, 2846, 1735, 1610, 1533, 1453, 1370, 1348, 1181, 1030, 858, 742, 508 cm^−1^. HRMS (ESI) calcd for C_18_H_18_N_2_NaO_6_S [M + Na]^+^: 413.0778; found: 413.0774.

*Ethyl cis-2-benzyl-3-(2-nitrophenyl)-1,2-thiazetidine-4-carboxylate 1,1-dioxide* (**5e**): Yellow oil, ^1^H-NMR (400 MHz, CDCl_3_): 8.15 (d, *J* = 7.9 Hz, 1H), 8.00 (d, *J* = 8.1 Hz, 1H), 7.67 (t, *J* = 7.6 Hz, 1H), 7.56–7.46 (m, 1H), 7.39–7.32 (m, 5H), 5.65 (d, *J* = 8.6 Hz, 1H), 5.04 (d, *J* = 8.6 Hz, 1H), 4.53 (d, *J* = 14.2 Hz, 1H), 4.13 (d, *J* = 14.3 Hz, 1H), 4.09–4.01 (m, 1H), 4.02–3.92 (m, 1H), 1.07 (t, *J* = 7.1 Hz, 3H). ^13^C-NMR (101 MHz, CDCl_3_): 162.1, 148.1, 134.0, 130.1, 129.8, 129.5, 128.9, 128.7, 128.7, 128.4, 125.2, 76.4, 62.4, 51.4, 51.0, 13.7. IR (KBr): ν 2987, 2920, 2846, 1735, 1610, 1533,1453, 1370, 1348, 1181, 1030, 858, 742, 508 cm^−1^. HRMS (ESI) calcd for C_18_H_18_N_2_NaO_6_S [M + Na]^+^: 413.0778; found: 413.0774.

*Ethyl trans-2-(tert-butyl)-3-(2-nitrophenyl)-1,2-thiazetidine-4-carboxylate 1,1-dioxide* (**3f**): Compounds *trans*-**3f** and *cis*-**5f** were isolated as a mixture, with a *trans/cis* ratio as 97:3, from a 1-mmol-scale reaction. Only the characteristic data of *trans*-**3f** are given. Colorless crystals, yield 65% (231 mg). M.p.: 145–150 °C, ^1^H-NMR (400 MHz, CDCl_3_): 8.25 (d, *J* = 7.8 Hz, 1H), 7.99 (d, *J* = 8.1 Hz, 1H), 7.77 (t, *J* = 7.6 Hz, 1H), 7.59–7.52 (m, 1H), 5.35 (d, *J* = 3.6 Hz, 1H), 4.65 (d, *J* = 2.0 Hz, 1H), 4.39 (q, *J* = 7.1 Hz, 2H), 1.35 (t, *J* = 7.1 Hz, 3H), 1.31 (s, 9H). ^13^C-NMR (101 MHz, CDCl_3_): 162.1, 148.0, 134.1, 129.7, 128.7, 124.8, 80.3, 63.1, 57.8, 45.1, 29.6, 27.7, 13.9, 0.9. IR (KBr): ν 2965, 2923, 1738, 1533, 1455, 1332, 1267, 1178, 1162, 1069, 742, 707 cm^−^^1^. HRMS (ESI) calcd for C_15_H_20_N_2_NaO_6_S [M + Na]^+^: 379.0934; found: 379.0930.

*Trans- and Cis-2-methyl-3-(2-nitrophenyl)-4-phenyl-1,2-thiazetidine 1,1-dioxides* (**3ac** and **5ac**): These two compounds were isolated as a mixture, with a *trans*/*cis* ratio as 52:48, from a 1-mmol-scale reaction. Since it was difficult to distinguish their ^13^C-NMR data. The analytical data are summarized together. Colorless crystals. M.p.: 118–125 °C. Yield 15% (47 mg). For *trans*-**3ac**, ^1^H-NMR (400 MHz, CDCl_3_): 8.19 (d, *J* = 8.0 Hz, 1H), 7.82–7.78 (m, 1H), 7.57–7.52 (m, 1H), 7.46 (s, 5H), 7.42–7.38 (m, 1H), 5.19 (d, *J* = 6.4 Hz, 1H), 5.04 (d, *J* = 6.4 Hz, 1H), 2.84 (s, 3H). For *cis*-**5ac**, ^1^H-NMR (400 MHz, CDCl_3_): 8.00–7.91 (m, 3H), 7.74–7.68 (m, 1H), 7.17–7.05 (m, 5H), 6.09 (d, *J* = 8.6 Hz, 1H), 5.27 (d, *J* = 8.6 Hz, 1H), 2.96 (s, 3H). ^13^C NMR (101 MHz, CDCl_3_): 144.5, 144.4, 134.4, 134.1, 131.8, 130.4, 130.0, 129.8, 129.44, 129.38, 129.29, 129.1, 128.9, 128.1, 128.0, 127.9, 127.43, 127.35, 125.7, 125.0, 84.5, 81.1, 57.7, 55.7, 32.1, 30.9. IR (KBr): ν 3070, 2920, 2337, 1524, 1447, 1351, 1322, 1168, 726, 508 cm^−1^. HRMS (ESI) calcd for C_15_H_14_N_2_NaO_4_S [M + Na]^+^: 341.0566; found 341.0563.

*Trans-2-methyl-3-(3-nitrophenyl)-4-phenyl-1,2-thiazetidine 1,1-dioxide* (**3bc**): Compounds **3bc** and **5bc** were isolated from a 1-mmol-scale reaction. Colorless crystals. M.p.: 118–125 °C. Yield: 15% (47 mg). ^1^H-NMR (400 MHz, CDCl_3_): 8.28 (s, 1H), 8.25–8.21 (m, 1H), 7.78 (d, *J* = 7.7 Hz, 1H), 7.61 (t, *J* = 7.9 Hz, 1H), 7.46 (s, 5H), 5.20 (d, *J* = 7.2 Hz, 1H), 4.36 (d, *J* = 7.2 Hz, 1H), 2.85 (s, 4H). ^13^C-NMR (101 MHz, CDCl_3_): 148.8, 138.0, 132.4, 130.4, 130.2, 129.3, 129.2, 127.6, 124.2, 121.6, 83.5, 60.3, 30.9. IR (KBr): ν 3070, 2920, 2337, 1524, 1447, 1351, 1322, 1168, 726, 508 cm^−1^. HRMS (ESI) calcd for C_15_H_14_N_2_NaO_4_S [M + Na]^+^: 341.0566; found 341.0563.

*Cis-2-methyl-3-(3-nitrophenyl)-4-phenyl-1,2-thiazetidine 1,1-dioxide* (**5bc**): Colorless crystals. M.p.: 154–160 °C. Yield 15% (48 mg) ^1^H-NMR (400 MHz, CDCl_3_): 8.06–7.96 (m, 2H), 7.49 (d, *J* = 7.7 Hz, 1H), 7.41–7.34 (m, 1H), 7.21–7.11 (m, 5H), 5.81 (d, *J* = 8.7 Hz, 1H), 4.81 (d, *J* = 8.6 Hz, 1H), 2.96 (s, 3H). ^13^C-NMR (101 MHz, CDCl_3_): 148.2, 135.9, 133.1, 129.9, 129.5, 129.3, 128.5, 127.5, 123.2, 122.2, 80.8, 58.0, 31.7. IR (KBr): ν 3070, 2920, 2337, 1524, 1447, 1351, 1322, 1168, 726, 508 cm^−1^. HRMS (ESI) calcd for C_15_H_14_N_2_NaO_4_S [M + Na]^+^: 341.0566; found 341.0563.

*Cis-2-methyl-3-(4-nitrophenyl)-4-phenyl-1,2-thiazetidine 1,1-dioxide* (**5cc**): Known compound [29]. Colorless crystals. M.p.: 182–183 °C. ^1^H-NMR (400 MHz, CDCl_3_): 8.20–7.10 (m, 9H, ArH), 5.83 (d, *J* = 8.8 Hz, 1H), 4.82 (d, *J* = 8.8 Hz, 1H), 2.96 (s, 3H).

*Trans-2-methyl-3-(4-nitrophenyl)-4-phenyl-1,2-thiazetidine 1,1-dioxide* (**3cc**): Known compound [29]. Colorless crystals. M.p.: 168–169 °C, ^1^H-NMR (400 MHz, CDCl_3_): 8.39–7.43 (m, 9H, ArH), 5.18 (d, *J* = 7.2 Hz, 1H), 4.37 (d, *J* = 7.2 Hz, 1H), 2.86 (s, 3H).

*Ethyl trans-2-propyl-3-(O-Tolyl)-1,2-thiazetidine-4-carboxylate 1,1-dioxide* (**3g**): Compounds *trans*-**3g** and *cis*-**5g** were isolated as a mixture, with a *trans/cis* ratio as 99:1, from a 1-mmol-scale reaction. Only the characteristic data of *trans*-**3g** are given. Colorless oil, yield 55% (171 mg). ^1^H-NMR (400 MHz, CDCl_3_): 7.72 (d, *J* = 7.5 Hz, 1H), 7.30 (t, *J* = 7.4 Hz, 1H), 7.26 (t, *J* = 6.9 Hz, 1H), 7.18 (d, *J* = 7.3 Hz, 1H), 4.82 (d, *J* = 6.3 Hz, 1H), 4.76 (d, *J* = 6.3 Hz, 1H), 4.40–4.32 (m, 1H), 4.32–4.25 (m, 1H), 3.33–3.20 (m, 1H), 2.86–2.75 (m, 1H), 2.41 (s, 3H), 1.66–1.52 (m, 2H), 1.33 (t, *J* = 7.1 Hz, 3H), 0.94 (t, *J* = 7.4 Hz, 3H). ^13^C-NMR (101 MHz, CDCl_3_): 162.4, 136.5, 134.2, 130.9, 128.8, 127.0, 126.0, 79.1, 63.1, 50.5, 48.5, 21.6, 19.2, 14.0, 11.6. IR (KBr): ν 2965, 2930, 2869, 1741, 1469, 1380, 1332, 1242, 1197, 1155, 1014, 752, 701 cm^−1^. HRMS (ESI) calcd for C_15_H_22_NO_4_S [M + H]^+^: 312.1264; found: 312.1256.

*Ethyl trans-2-propyl-3-(2-(trifluoromethyl)phenyl)-1,2-thiazetidine-4-carboxylate 1,1-dioxide* (**3h**): Compounds *trans*-**3h** and *cis*-**5h** were isolated as a mixture, with a *trans/cis* ratio as 97:3, from a 1-mmol-scale reaction. Only the characteristic data of *trans*-**3h** are given. Colorless oil, yield: 37% (135 mg). ^1^H-NMR (400 MHz, CDCl_3_): 8.09 (d, *J* = 7.9 Hz, 1H), 7.71 (t, *J* = 7.5 Hz, 2H), 7.51 (t, *J* = 7.6 Hz, 1H), 5.04 (d, *J* = 5.2 Hz, 1H), 4.78 (d, *J* = 5.2 Hz, 1H), 4.40–4.33 (m, 1H), 4.33–4.26 (m, 1H), 3.36–3.23 (m, 1H), 2.96–2.82 (m, 1H), 1.62–1.48 (m, 2H), 1.33 (t, *J* = 7.1 Hz, 3H), 0.91 (t, *J* = 7.4 Hz, 3H). ^13^C-NMR (101 MHz, CDCl_3_): 161.6, 135.43 (q, *J* = 1.2 Hz), 133.0, 129.1, 128.75 (q, *J* = 30.8 Hz), 128.2, 126.05 (q, *J* = 5.8 Hz, 1H), 123.72 (q, *J* = 273.9 Hz), 80.3, 63.1, 49.2 (q, *J* = 2.3 Hz), 48.3, 21.4, 13.9, 11.5. IR (KBr): ν 2974, 2929, 1747, 1453, 1312, 1267, 1164, 1119, 1036, 774 cm^−1^. HRMS (ESI) calcd for C_15_H_19_F_3_NO_4_S [M + H]^+^: 366.0981; found: 366.0977.

*Ethyl trans-3-(2-methoxyphenyl)-2-propyl-1,2-thiazetidine-4-carboxylate 1,1-dioxide* (**3i**): Compounds *trans*-**3i** and *cis*-**5i** were isolated as a mixture, with a *trans/cis* ratio as 99:1, from a 1-mmol-scale reaction. Only the characteristic data of *trans*-**3i** are given. Colorless oil, yield 78% (255 mg). ^1^H-NMR (400 MHz, CDCl_3_): 7.55 (d, *J* = 7.6 Hz, 1H), 7.38–7.30 (m, 1H), 7.02 (t, *J* = 7.5 Hz, 1H), 6.89 (d, *J* = 8.2 Hz, 1H), 4.84 (d, *J* = 6.1 Hz, 1H), 4.81 (d, *J* = 6.0 Hz, 1H), 4.41–4.33 (m, 1H), 4.34–4.25 (m, 1H), 3.81 (s, 3H), 3.38–3.25 (m, 1H), 2.91–2.80 (m, 1H), 1.75–1.55 (m, 2H), 1.34 (t, *J* = 7.1 Hz, 3H), 0.97 (t, *J* = 7.4 Hz, 3H). ^13^C-NMR (101 MHz, CDCl_3_): 162.4, 157.1, 130.0, 126.8, 124.5, 120.9, 110.6, 78.1, 62.6, 55.3, 49.14, 49.00, 21.7, 14.1, 11.6. IR (KBr): ν 2968, 2927, 2875, 1748, 1604, 1492, 1335, 1245, 1191, 1024, 755, 710 cm^−1^. HRMS (ESI) calcd for C_15_H_22_NO_5_S [M + H]^+^: 328.1213; found: 328.1206.

*Ethyl trans-3-(2fluorophenyl)-2-propyl-1,2-thiazetidine-4-carboxylate 1,1-dioxide* (**3j**): Compounds was isolated from a 0.25-mmol-scale reaction. Pale yellow oil. Yield: 36% (27 mg). ^1^H-NMR (400 MHz, CDCl_3_) δ 7.68 (td, *J* = 7.6, 1.6 Hz, 1H), 7.43–7.35 (m, 1H), 7.30–7.23 (m, 1H), 7.16–7.07 (m, 1H), 4.91 (s, 2H), 4.47–4.27 (m, 2H), 3.37–3.25 (m, 1H), 2.93–2.80 (m, 1H), 1.71–1.54 (m, 2H), 1.36 (t, *J* = 7.1 Hz, 3H), 0.97 (t, *J* = 7.4 Hz, 3H). ^13^C-NMR (101 MHz, CDCl_3_) δ 161.9, 160.79 (d, *J* = 249.1 Hz), 130.81 (d, *J* = 8.2 Hz), 127.79 (d, *J* = 3.1 Hz), 124.92 (d, *J* = 3.7 Hz), 123.35 (d, *J* = 12.4 Hz), 115.93 (d, *J* = 20.9 Hz), 78.2, 63.11, 48.7, 47.83, 47.80, 21.5, 14.0, 11.5. IR (KBr): ν 2968, 2935, 2875, 1745, 1491, 1458, 1372, 1338, 1237, 1191, 1159, 1100, 1015, 763 cm^−1^. HRMS (ESI) calcd for C_14_H_18_FNNaO_4_S [M + Na]^+^ 338.0833, found 338.0839.

*Ethyl trans-3-(2-chlorophenyl)-2-propyl-1,2-thiazetidine-4-carboxylate 1,1-dioxide* (**3k**): Compounds *trans*-**3j** and *cis*-**5j** were isolated as a mixture, with a *trans/cis* ratio as 97:3, from a 1-mmol-scale reaction. Only the characteristic data of *trans*-**3j** are given. Colorless crystals, yield 68% (225 mg), M.p.: 51–59 °C. ^1^H-NMR (400 MHz, CDCl_3_): 7.78 (d, *J* = 7.8 Hz, 1H), 7.39 (t, *J* = 8.2 Hz, 2H), 7.35–7.29 (m, 1H), 5.05 (d, *J* = 5.6 Hz, 1H), 4.77 (d, *J* =5.6 Hz, 1H), 4.41–4.35 (m, 1H), 4.34–4.28 (m, 1H), 3.41–3.28 (m, 1H), 2.96–2.79 (m, 1H), 1.73–1.53 (m, 2H), 1.34 (t, *J* = 7.1 Hz, 3H), 0.96 (t, *J* = 7.4 Hz, 3H). ^13^C-NMR (101 MHz, CDCl_3_): 162.0, 134.2, 133.3, 130.2, 130.0, 127.68, 127.66, 78.9, 63.1, 50.4, 48.9, 21.6, 14.0, 11.6. IR (KBr): ν 2962, 2933, 2869, 1741, 1475, 1469, 1341, 1187, 1040, 755, 701, 518 cm^−1^. HRMS (ESI) calcd for C_14_H_19_ClNO_4_S [M + H]^+^: 332.0718; found: 332.0713.

*Ethyl trans-3-(2-bromophenyl)-2-propyl-1,2-thiazetidine-4-carboxylate 1,1-dioxide* (**3l**): Compounds *trans*-**3k** and *cis*-**5k** were isolated as a mixture, with a *trans/cis* ratio as 97:3, from a 1-mmol-scale reaction. Only the characteristic data of *trans*-**3k** are given. Colorless crystals, yield: 70% (262 mg), M.p.: 76–81 °C. ^1^H-NMR (400 MHz, CDCl_3_): 7.82–7.77 (m, 1H), 7.58 (d, *J* = 7.9 Hz, 1H), 7.43 (t, *J* = 7.5 Hz, 1H), 7.24 (dd, *J* = 11.5, 5.0 Hz, 1H), 5.04 (d, *J* = 5.5 Hz, 1H), 4.75 (d, *J* = 5.5 Hz, 1H), 4.42–4.35 (m, 1H), 4.35–4.27 (m, 1H), 3.40–3.26 (m, 1H), 2.97–2.83 (m, 1H), 1.70–1.52 (m, 2H), 1.34 (t, *J* = 7.1 Hz, 3H), 0.96 (t, *J* = 7.4 Hz, 3H). ^13^C-NMR (101 MHz, CDCl_3_): 162.0, 135.9, 133.3, 130.5, 128.3, 128.0, 123.0, 79.2, 63.1, 52.6, 48.7, 21.5, 14.0, 11.5; IR (KBr): ν 2968, 2933, 2872, 1738, 1469, 1370, 1332, 1261, 1184, 1030, 758, 518 cm^−1^. HRMS (ESI) calcd for C_14_H_19_BrNO_4_S [M + H]^+^: 376.0213; found: 376.0204.

*Ethyl trans-3-(2,6-dichlorophenyl)-2-propyl-1,2-thiazetidine-4-carboxylate 1,1-dioxide* (**3m**): Compounds *trans*-**3l** and *cis*-**5l** were isolated as a mixture, with a *trans/cis* ratio as 99:1, from a 1-mmol-scale reaction. Only the characteristic data of *trans*-**3l** are given. Colorless crystals, yield 53% (193 mg), M.p.: 116–122 °C. ^1^H-NMR (400 MHz, CDCl_3_): 7.39 (d, *J* = 7.8 Hz, 2H), 7.30–7.24 (m, 1H), 5.82 (d, *J* = 7.1 Hz, 1H), 5.52 (d, *J* = 7.1 Hz, 1H), 4.40–4.34 (m, 1H), 4.32–4.25 (m, 1H), 3.32–3.25 (m, 1H), 2.81–2.74 (m, 1H), 1.54–1.41 (m, 2H), 1.34 (t, *J* = 7.1 Hz, 3H), 0.87 (t, *J* = 7.4 Hz, 3H). ^13^C-NMR (101 MHz, CDCl_3_): 162.3, 136.8, 130.8, 130.01, 129.96, 128.2, 128.1, 73.9, 63.1, 50.7, 48.9, 21.3, 14.0, 11.5. IR (KBr): ν 2971, 2939, 2879, 1738, 1565, 1434, 1389, 1344, 1261, 1194, 1155, 1091, 1011, 774, 518 cm^−1^. HRMS (ESI) calcd for C_14_H_17_Cl_2_NNaO_4_S [M + Na]^+^: 388.0148; found: 388.0371.

*Ethyl trans-3-([1,1′-biphenyl]-2-yl)-2-propyl-1,2-thiazetidine-4-carboxylate 1,1-dioxide* (**3n**): Only compound *trans*-**3m** was isolated from a 1-mmol-scale reaction, and the characteristic data are given as following. Yellow oil, yield: 258 mg (69%). ^1^H-NMR (400 MHz, CDCl_3_): 7.85 (d, *J* = 7.8 Hz, 1H), 7.49 (dd, *J* = 11.0, 4.2 Hz, 1H), 7.45–7.36 (m, 4H), 7.25–7.17 (m, 3H), 4.82 (d, *J* = 5.8 Hz, 1H), 4.71 (d, *J* = 5.8 Hz, 1H), 4.15 (q, *J* = 7.1 Hz, 2H), 3.21–3.05 (m, 1H), 2.78–2.71 (m, 1H), 1.55–1.42 (m, 2H), 1.23 (t, *J* = 7.1 Hz, 4H), 0.88 (t, *J* = 7.4 Hz, 3H). ^13^C-NMR (101 MHz, CDCl_3_): 161.6, 142.7, 139.4, 133.9, 130.3, 129.3, 128.6, 128.5, 128.4, 127.7, 126.4, 79.6, 62.8, 50.3, 48.0, 21.5, 13.9, 11.5. IR (KBr): ν 2962, 2933, 2869, 1735, 1601, 1482, 1364, 1332, 1258, 1191, 1001, 1011, 861, 752, 697, 521 cm^−1^. HRMS (ESI) calcd for C_20_H_24_NO_4_S [M + H]^+^: 374.1421; found: 374.1430.

*Ethyl trans-3-(naphthalen-2-Yl)-2-propyl-1,2-thiazetidine-4-carboxylate 1,1-dioxide* (**3o**): Only compound *trans*-**3n** was isolated, and the characteristic data are given as following. Colorless crystals. Yield: 147 mg (85%). M.p: 114–118 °C. ^1^H-NMR (400 MHz, CDCl_3_): 7.96 (d, *J* = 0.8 Hz, 1H), 7.91 (d, *J* = 8.5 Hz, 1H), 7.89–7.82 (m, 2H), 7.65 (dd, *J* = 8.5, 1.7 Hz, 1H), 7.58–7.49 (m, 2H), 4.90 (d, *J* = 6.4 Hz, 1H), 4.72 (d, *J* = 6.4 Hz, 1H), 4.42–4.33 (m, 1H), 4.33–4.25 (m, 1H), 3.28 (ddd, *J* = 12.9, 8.2, 7.1 Hz, 1H), 2.86 (ddd, *J* = 12.9, 8.2, 7.1 Hz, 1H), 1.68–1.52 (m, 2H), 1.33 (t, *J* = 7.1 Hz, 3H), 0.93 (t, *J* = 7.4 Hz, 3H). ^13^C-NMR (101 MHz, CDCl_3_): 162.3, 133.6, 133.2, 133.1, 129.4, 128.0, 127.8, 126.84, 126.83, 126.6, 123.4, 79.0, 63.1, 54.4, 48.4, 21.6, 14.0, 11.6. IR (KBr): ν 2969, 2920, 2870, 1735, 1469, 1332, 1194, 1162, 1024, 819, 745, 473 cm^−1^. HRMS (ESI) calcd for C_18_H_22_NO_4_S [M + H]^+^: 348.1264; found 348.1274.

*Ethyl trans-3-(2,4-dinitrophenyl)-2-propyl-1,2-thiazetidine-4-carboxylate 1,1-dioxide* (**3p**): Compounds *trans*-**3o** and *cis*-**5o** were isolated as a mixture, with a *trans/cis* ratio as 75:25, from a 1-mmol-scale reaction. Yellow oil, total yield 60% (232 mg). ^1^H-NMR (400 MHz, CDCl_3_): 8.89 (s, 1H), 8.61 (d, *J* = 7.3 Hz, 1H), 8.44 (d, *J* = 8.7 Hz, 1H), 5.14 (d, *J* = 4.6 Hz, 1H), 4.84 (d, *J* = 4.6 Hz, 1H), 4.41 (q, *J* = 7.1 Hz, 2H), 3.49–3.35 (m, 1H), 3.05–2.89 (m, 1H), 1.63 (dq, *J* = 14.7, 7.2 Hz, 2H), 1.37 (t, *J* = 7.1 Hz, 3H), 0.99 (t, *J* = 7.3 Hz, 3H). ^13^C-NMR (101 MHz, CDCl_3_): 161.8, 148.3, 148.2, 138.9, 130.6, 128.4, 120.7, 80.4, 63.6, 63.0, 50.0, 49.5, 21.6, 14.0, 11.5; IR (KBr): ν 2965, 2933, 2872, 1741, 1607, 1540, 1466, 1348, 1258, 1184, 1002, 1009, 835, 796 cm^−1^. HRMS (ESI) calcd for C_14_H_18_N_3_O_8_S [M + H]^+^: 388.0809; found: 388.0802.

*Ethyl cis-3-(2,4-dinitrophenyl)-2-propyl-1,2-thiazetidine-4-carboxylate 1,1-dioxide* (**5p**): ^1^H-NMR (400 MHz, CDCl_3_): 9.02 (s, 1H), 8.61 (d, *J* = 7.3 Hz, 1H), 8.32 (d, *J* = 8.7 Hz, 1H), 5.71 (d, *J* = 8.6 Hz, 1H), 4.99 (d, *J* = 8.6 Hz, 1H), 4.17–4.08 (m, 1H), 4.09–3.99 (m, 1H), 3.49–3.36 (m, 1H), 2.86–2.75 (m, 1H), 1.81–1.69 (m, 2H), 1.19 (t, *J* = 7.1 Hz,3H), 1.06 (t, *J* = 7.4 Hz, 3H). ^13^C-NMR (101 MHz, CDCl_3_): 161.4, 147.9, 147.7, 137.9, 131.7, 127.9, 120.7, 76.5, 63.6, 51.3, 49.8, 21.8, 13.8, 11.6. IR (KBr): ν 2965, 2933, 2872, 1741, 1607, 1540, 1466, 1348, 1258, 1184, 1002, 1009, 835, 796 cm^−1^. HRMS (ESI) calcd for C_14_H_18_N_3_O_8_S [M + H]^+^: 388.0809; found: 388.0802.

*Ethyl trans-3-(5-methoxy-2-nitrophenyl)-2-propyl-1,2-thiazetidine-4-carboxylate 1,1-dioxide* (**3q**): Compounds *trans*-**3q** and *cis*-**5q** were isolated as a mixture, with a *trans/cis* ratio as 85:15, from a 0.25-mmol-scale reaction. Colorless oil. Yield: 68% (63 mg).

NMR data for the *trans*-isomer: ^1^H-NMR (400 MHz, CDCl_3_) δ 8.18 (d, *J* = 9.1 Hz, 1H), 7.65 (d, *J* = 2.7 Hz, 1H), 7.01 (dd, *J* = 9.1, 2.8 Hz, 1H), 5.16 (d, *J* = 4.5 Hz, 1H), 4.78 (d, *J* = 4.5 Hz, 1H), 4.41 (q, *J* = 7.1 Hz, 2H), 3.97 (s, *3*H), 3.52–3.39 (m, 1H), 3.06–2.94 (m, 1H), 1.73–1.59 (m, 2H), 1.38 (t, *J* = 7.1 Hz, 3H), 1.03 (t, *J* = 7.3 Hz, 3H). ^13^C-NMR (101 MHz, CDCl_3_): 164.5, 162.5, 140.6, 135.8, 128.4, 114.4, 113.5, 80.5, 63.2, 56.1, 50.7, 49.3, 21.7, 14.0, 11.6.

NMR data for the *cis*-isomer: ^1^H-NMR (400 MHz, CDCl_3_) δ 8.26 (d, *J* = 9.1 Hz, 1H), 7.53 (d, *J* = 2.7 Hz, 1H), 7.01 (dd, *J* = 9.1, 2.8 Hz, 1H), 5.65 (d, *J* = 8.6 Hz, 1H), 5.00 (d, *J* = 8.6 Hz, 1H), 4.41 (q, *J* = 7.1 Hz, 2H), 3.97 (s, 3H), 3.47–3.36 (m, 1H), 2.82 (dt, *J* = 12.7, 7.9 Hz, 1H), 1.83–1.72 (m, 2H), 1.38 (t, *J* = 7.1 Hz, 3H), 1.03 (t, *J* = 7.3 Hz, 3H). ^13^C-NMR (101 MHz, CDCl_3_): 164.2, 161.4, 133.9, 128.3, 114.8, 114.4, 114.0, 76.1, 62.3, 56.1, 51.9, 49.6, 21.9, 13.7, 11.7.

IR (KBr): ν 2968, 2939, 2875, 1747, 1612, 1581, 1517, 1484, 1466, 1370, 1340, 1288, 1240, 1230, 1182, 1085, 1033, 860, 848, 828, 754 cm^−1^. HRMS (ESI) calcd for C_15_H_20_N_2_NaO_7_S [M + Na]^+^: 395.0883; found: 395.0885.

### 3.3. General Procedure for the Staudinger Cycloadditions of Ethyl Malonyl Chloride *(**1b**)*

To a solution of imine **2** (0.5 mmol) and 2-chloropyridne (113 μL, 1.2 mmol) in dry THF (3 mL) was dropwise added a solution of ethyl malonyl chloride (**1b**) (0.181 g, 1.2 mmol) in dry THF (2 mL) via a syringe for 3 min. Upon addition, the resulting mixture was allowed to stir at room temperature for 12 h. Then ether (15 mL) and brine (10 mL) were added sequentially. After washing, the organic phase was dried over MgSO_4_. Removal of the solvent and purification on silica gel chromatography afforded the desired corresponding products *trans*-**4** and *cis*-**6**.

*Ethyl trans-4-(2-nitrophenyl)-1-propylazetidin-2-one-3-carboxylate* (**4d**): Compounds *trans*-**4d** and *cis*-**6d** were isolated as a mixture, with a *trans/cis* ratio as 90:10. Yellowish oil. Yield 43% (65 mg). ^1^H-NMR (400 MHz, CDCl_3_): 8.14–8.08 (m, 1H), 7.77–7.71 (m, 1H), 7.60–7.52 (m, 2H), 5.31 (d, *J* = 2.3 Hz, 1H), 4.40–4.33 (m, 1H), 4.33–4.26 (m, 1H), 3.79 (d, *J* = 2.2 Hz, 1H), 3.72–3.61 (m, 1H), 2.96–2.88 (m, 1H), 1.69–1.57 (m, 2H), 1.34 (t, *J* = 7.1 Hz, 3H), 1.00 (t, *J* = 7.4 Hz, 3H). ^13^C-NMR (100 MHz, CDCl_3_): 166.6, 162.9, 148.3, 134.2, 133.2, 129.5, 126.7, 125.5, 63.7, 61.9, 54.1, 43.5, 20.8, 14.1, 11.2. IR (KBr): ν 1770, 1731, 1526, 1349, 1190, 1041 cm^−1^. HRMS (ESI) calcd for C_15_H_19_N_2_O_5_ [M + H]^+^: 307.1288; found 307.1294.

*Ethyl cis-4-(2-nitrophenyl)-1-propylazetidin-2-one-3-carboxylate* (**6d**): Yellowish oil. ^1^H-NMR (400 MHz, CDCl_3_): 8.18 (dd, *J* = 8.2, 1.1 Hz, 1H), 7.72–7.71 (m, 1H), 7.66–7.62 (m, 1H), 7.55–7.53 (m, 1H), 5.52 (d, *J* = 5.9 Hz, 1H), 4.62 (d, *J* = 5.9 Hz, 1H), 3.82 (q, *J* = 7.1 Hz, 2H), 3.72–3.61 (m, 1H), 3.03–2.97 (m, 1H), 1.70–1.58 (m, 2H), 0.94 (t, *J* = 7.4 Hz, 4H), 0.90 (t, *J* = 7.1 Hz, 3H). ^13^C-NMR (100 MHz, CDCl_3_): 165.4, 163.3, 147.8, 133.6, 131.6, 129.3, 128.7, 125.4, 61.1, 60.4, 54.2, 43.6, 20.6, 13.7, 11.5. IR (KBr): ν 1770, 1731, 1526, 1349, 1190, 1041 cm^−1^. HRMS (ESI) calcd for C_15_H_19_N_2_O_5_ [M + H]^+^: 307.1288; found 307.1288.

*Ethyl trans-4-(3-nitrophenyl)-1-propylazetidin-2-one-3-carboxylate* (**4r**): Only the *trans*-**4r** was isolated, and the characteristic data are given as following. Yellowish oil. Yield: 54% (82 mg).^1^H-NMR (400 MHz, CDCl_3_): 8.29–8.20 (m, 2H), 7.72 (d, *J* = 7.7 Hz, 1H), 7.65 (dd, *J* = 7.8 Hz, 1H), 5.00 (d, *J* = 2.2 Hz, 1H), 4.34–4.28 (m, 1H), 4.28–4.22 (m, 1H), 3.87 (d, *J* = 2.2 Hz, 1H), 3.55–3.43 (m, 1H), 2.95–2.83 (m, 1H), 1.63–1.49 (m, 2H), 1.33 (t, *J* = 7.1 Hz, 3H), 0.95 (t, *J* = 7.4 Hz, 3H). ^13^C-NMR (100 MHz, CDCl_3_): 166.2, 161.9, 148.7, 139.1, 132.4, 130.3, 123.9, 121.7, 63.4, 62.0, 56.4, 43.0, 20.8, 14.0, 11.2. IR (KBr): ν 1770, 1735, 1533, 1351, 1200, 1093, 1041, 1013 cm^−1^. HRMS (ESI) calcd for C_15_H_19_N_2_O_5_ [M + H]^+^: 307.1288; found 307.1288.

*Ethyl trans-4-(4-nitrophenyl)-1-propylazetidin-2-one-3-carboxylate* (**4s**): Only the *trans*-**4s** was isolated, and the characteristic data are given as following. Known compound [47]. Colorless crystals, Yield: 58% (95 mg). M.p.: 84–85 °C. ^1^H-NMR (400 MHz, CDCl_3_): 8.28 (d, *J* = 8.6 Hz, 2H, ArH), 7.54 (d, *J* = 8.6 Hz, 2H, ArH), 4.98 (d, *J* = 2.1 Hz, 1H), 4.28 (q, *J* = 7.1 Hz, 2H), 3.84 (d, *J* = 2.1 Hz, 1H), 3.53–3.43 (m, 1H), 2.91–2.84 (m, 1H), 1.62–1.49 (m, 2H), 1.32 (t, *J* = 7.1 Hz, 3H), 0.94 (t, *J* = 7.4 Hz, 3H). ^13^C-NMR (100 MHz, CDCl_3_): 166.3, 161.9, 148.3, 144.0, 127.5, 124.4, 63.4, 62.1, 56.4, 43.1, 20.9, 14.0, 11.3.

*Ethyl trans-1-methyl-2-(2-nitrophenyl)-4-oxoazetidine-3-carboxylate* (**4a**): Compounds *trans*-**4a** and *cis*-**6a** were isolated as a mixture, with a *trans/cis* ratio as 85:15. Yellowish oil. Total yield: 18% (28 mg). ^1^H-NMR (400 MHz, CDCl_3_): 8.12 (dd, *J* = 8.2, 1.1 Hz, 1H), 7.74 (ddd, *J* = 7.6, 7.6, 0.9 Hz, 1H), 7.60–7.50 (m, 2H), 5.25 (d, *J* = 2.4 Hz, 1H), 4.40–4.33 (m, 1H), 4.33–4.27 (m, 1H), 3.83 (d, *J* = 2.4, 1H), 2.97 (d, *J* = 0.6 Hz, 3H), 1.34 (t, *J* = 7.1 Hz, 4H). ^13^C-NMR (100 MHz, CDCl_3_): 166.5, 162.9, 145.4, 134.3, 133.0, 129.5, 126.5, 125.6, 64.3, 62.0, 56.3, 28.4, 14.1. IR (KBr): ν 1772, 1731, 1526, 1349, 1260, 1190, 1085 cm^−1^. HRMS (ESI) calcd for C_13_H_15_N_2_O_5_ [M + H]^+^: 279.0975; found 279.0977.

*Ethyl cis-1-methyl-2-(2-nitrophenyl)-4-oxoazetidine-3-carboxylate* (**6a**): Yellowish oil. ^1^H-NMR (400 MHz, CDCl_3_): 8.20 (dd, *J* = 8.2, 1.2 Hz, 1H), 7.60–7.50 (m, 1H), 7.35–7.27 (m, 2H), 5.43 (d, *J* = 5.8 Hz, 1H), 4.65 (d, *J* = 5.8 Hz, 1H), 3.84 (q, *J* = 7.2 Hz, 2H), 2.98 (s, 3H), 1.28 (t, *J* = 7.2 Hz, 3H). IR (KBr): ν 1772, 1731, 1526, 1349, 1260, 1190, 1085 cm^−1^. HRMS (ESI) calcd for C_13_H_15_N_2_O_5_ [M + H]^+^: 279.0975; found 279.0977.

*Ethyl trans-1-benzyl-2-(2-nitrophenyl)-4-oxoazetidine-3-carboxylate* (**4e**): Compounds *trans*-**4e** and *cis*-**6e** were isolated as a mixture, with a *trans/cis* ratio as 95:5. Yellowish oil. Yield 34% (60 mg). ^1^H-NMR (400 MHz, CDCl_3_): 8.07 (dd, *J* = 8.2, 1.1 Hz, 1H), 7.70 (td, *J* = 7.8, 0.9 Hz, 1H), 7.55 (ddd, *J* = 15.5, 8.2, 4.1 Hz, 2H), 7.35–7.27 (m, 3H), 7.26–7.21 (m, 2H), 5.16 (d, *J* = 2.4 Hz, 1H), 5.02 (d, *J* = 15.3 Hz, 1H), 4.38–4.31 (m, 1H), 4.31–4.24 (m, 1H), 4.05 (d, *J* = 15.3 Hz, 1H), 3.85 (d, *J* = 2.1 Hz, 1H), 1.32 (t, *J* = 7.2 Hz, 3H). ^13^C-NMR (100 MHz, CDCl_3_): 166.3, 163.0, 148.1, 134.1, 134.0, 133.0, 129.4, 128.9, 128.2, 128.1, 126.7, 125.5, 64.0, 61.9, 54.1, 45.8, 20.9, 14.0. IR (KBr): ν 1771, 1735, 1526, 1348, 1188 cm^−1^. HRMS (ESI) calcd for C_19_H_19_N_2_O_5_ [M + H]^+^: 355.1288; found 355.1294.

*Ethyl trans-1-(tert-butyl)-2-(2-nitrophenyl)-4-oxoazetidine-3-carboxylate* (**4f**): Yellowish oil. Yield 33% (51 mg). ^1^H-NMR (400 MHz, CDCl_3_): 8.01 (dd, *J* = 8.2, 1.0 Hz, 1H), 7.83 (dd, *J* = 7.9, 1.1 Hz, 1H), 7.75–7.69 (m, 1H), 7.55–7.49 (m, 1H), 5.34 (d, *J* = 2.3 Hz, 1H), 4.38–4.31 (m, 1H), 4.31–4.24 (m, 1H), 3.61 (d, *J* = 2.3 Hz, 1H), 1.34 (s, 9H), 1.33 (t, *J* = 7.2 Hz, 3H). ^13^C-NMR (100 MHz, CDCl_3_): 166.6, 163.2, 148.2, 135.2, 133.7, 129.3, 127.3, 125.0, 62.7, 61.9, 55.4, 52.4, 27.8, 14.1. IR (KBr): ν 1766, 1732, 1370, 1348, 1321, 1259, 1224, 1183 cm^−1^. HRMS (ESI) calcd for C_16_H_21_N_2_O_5_ [M + H]^+^: 321.1445; found 321.1452.

*Ethyl cis-1-(tert-butyl)-2-(2-nitrophenyl)-4-oxoazetidine-3-carboxylate* (**6f**): Yellowish oil. Yield 18% (22 mg). ^1^H-NMR (400 MHz, CDCl_3_): 8.08 (dd, *J* = 8.2, 1.1 Hz, 1H), 7.86 (dd, *J* = 7.9, 1.0 Hz, 1H), 7.72–7.66 (m, 1H), 7.56–7.48 (m, 1H), 5.58 (d, *J* = 6.3 Hz, 1H), 4.48 (d, *J* = 6.3 Hz, 1H), 3.78 (q, *J* = 7.2 Hz, 2H), 1.35 (s, 10H), 0.87 (t, *J* = 7.1 Hz, 3H). ^13^C-NMR (100 MHz, CDCl_3_): 165.4, 164.2, 148.2, 133.0, 132.9, 129.4, 129.3, 125.0, 61.1, 58.9, 55.4, 52.6, 27.8, 13.6. IR (KBr): ν 1766, 1734, 1371, 1348, 1322, 1259, 1223, 1183 cm^−1^. HRMS (ESI) calcd for C_16_H_21_N_2_O_5_ [M + H]^+^: 321.1445; found 321.1452.

*Ethyl trans-1-(tert-butyl)-4-phenylazetidin-2-one-3-carboxylate* (**4t**): Known compound [49]. Colorless crystals, yield 69% (95 mg); M.p.: 91–94 °C. ^1^H-NMR (400 MHz, CDCl_3_): 7.41–7.32 (m, 5H, ArH), 4.85 (d, *J* = 2.0 Hz, 1H), 4.234 (q, *J* = 7.2, 1H), 4.225 (q, *J* = 7.2, 1H), 3.69 (d, *J* = 2.0 Hz, 1H), 1.29 (t, *J* = 7.2 Hz, 3H), 1.27 (s, 9H).

## 4. Conclusions

In conclusion, the *ortho*-nitro effect was discovered in sulfa-Staudinger cycloadditions of ethoxycarbonylsulfene with *C*-2-nitroarylimines, as well as in Staudinger cycloadditions of ethoxycarbonylketene with *C*-2-nitroarylimines. When an *ortho*-nitro group is present at the *C*-aryl substituents of linear imines, sulfa-Staudinger and Staudinger cycloadditions deliver the corresponding *cis*-β-sultams and *cis*-β-lactams in considerable amounts, respectively, together with the corresponding predominant *trans*-β-sultams and *trans*-β-lactams. In other cases without *C*-2-nitroarylimines, the above sulfa-Staudinger and Staudinger cycloadditions produce *trans*-β-sultams and *trans*-β-lactams, respectively, in excellent diastereoselectivities (*trans/cis* ratios from 97:3 to 100:0). Further mechanistic rationalization discloses that the *ortho*-nitro effect is attributed to its strong electron-withdrawing inductive effect rather than the steric hindrance of the 2-nitroaryl group and the electrostatic interaction between the *ortho*-nitro group and the ethoxycarbonyl group. The current research provides further insights into the diastereoselective control in sulfa-Staudinger and Staudinger cycloadditions.

## Figures and Tables

**Table 1:**
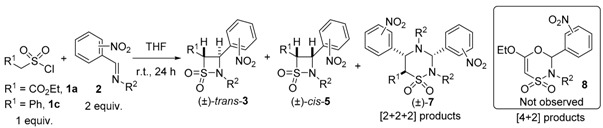
Sulfa-Staudinger cycloadditions involving *C*-nitrophenyl imines ^a^.

Entry	R^1^	NO_2_	Imine	R^2^	Yield (%) ^b^	*trans/cis* Ratio ^c^		7
1	EtO_2_C	2-NO_2_	**2a**	Me	66	**3a**:**5a**	82:18	0
2	EtO_2_C	3-NO_2_	**2b**	Me	79 ^d^	**3b**:**5b**	100:0	13
3	EtO_2_C	4-NO_2_	**2c**	Me	41 ^d^	**3c**:**5c**	100:0	3
4	EtO_2_C	2-NO_2_	**2d**	*n*-Pr	66	**3d**:**5d**	86:14	0
5	EtO_2_C	2-NO_2_	**2e**	Bn	71	**3e**:**5e**	88:12	0
6	EtO_2_C	2-NO_2_	**2f**	*t*-Bu	65	**3f**:**5f**	97:3	0
7	Ph	2-NO_2_	**2a**	Me	36	**3ac**:**5ac**	52:48	0
8	Ph	3-NO_2_	**2b**	Me	30	**3bc**:**5bc**	54:46	0
9	Ph	4-NO_2_	**2c**	Me	37	**3cc**:**5cc**	43:57	0
10 ^e^	EtO_2_C	2-NO_2_	**2a**	Me	97	**3a**:**5a**	63:37	0
11 ^e^	EtO_2_C	3-NO_2_	**2b**	Me	92	**3b**:**5b**	100:0	0
12 ^e^	EtO_2_C	4-NO_2_	**2c**	Me	99	**3c**:**5c**	100:0	0

^a^ Reactions conducted on a 0.5-mmol scale based on sulfonyl chloride; The data in entries 1–3 were directly cited from refs. [27,28]. ^b^ Isolated total yield; ^c^ Ratios were determined by the ^1^H-NMR analysis of the isolated mixture. ^d^ Also see ref. [27]. ^e^ These reactions were performed with 1.2 equivalents of sulfonyl chloride **1a**, 1.0 equivalent of imines **2a**–**c**, and 1.2 equivalents of pyridine.

**Table 2:**
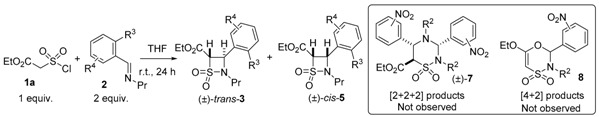
Sulfa-Staudinger reactions of *C*-2-substitutedaryl imines with ethoxycarbonylmethanesulfonyl chloride (**1a**) ^a^.

Entry	Imine	R^3^	R^4^	Yield (%) ^b^	*trans/cis* Ratio ^c^	
1	**2a**	NO_2_	H	66	**3a**:**5a**	82:18
2	**2g**	Me	H	55	**3**:**5g**	99:1
3	**2h**	CF_3_	H	37	**3h**:**5h**	97:3
4	**2i**	OMe	H	78	**3i**:**5i**	99:1
5	**2j**	F	H	72	**3j**:**5j**	95:5
6	**2k**	Cl	H	68	**3k**:**5k**	97:3
7	**2l**	Br	H	70	**3l**:**5l**	97:3
8	**2m**	Cl	6-Cl	53	**3m:5m**	99:1
9	**2n**	Ph	H	69	**3n:5n**	100:0
10	**2o**	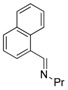		85	**3o:5o**	100:0
11	**2p**	NO_2_	4-NO_2_	60	**3p:5p**	75:25
12	**2q**	NO_2_	5-MeO	87	**3q:5q**	56:44

^a^ Reactions conducted on a 0.5-mmol scale based on sulfonyl chloride. ^b^ Isolated total yield. ^c^ Ratios were determined by the ^1^H-NMR analysis of the isolated mixture.

**Table 3:**
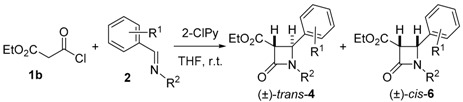
*Ortho*-nitro effect in Staudinger reactions ^a^.

Entry	Imine	R^1^	R^2^	Yield (%) ^b^	*Trans/cis* Ratio ^c^	
1	**2d**	2-NO_2_	Pr	43	**4d**:**6d**	83:17
2	**2r**	3-NO_2_	Pr	54	**4r**:**6r**	100:0
3	**2s**	4-NO_2_	Pr	58	**4s**:**6s**	100:0
4	**2a**	2-NO_2_	Me	18	**4a**:**6a**	85:15
5	**2e**	2-NO_2_	Bn	34	**4e**:**6e**	81:19
6	**2f**	2-NO_2_	*t*-Bu	51	**4f**:**6f**	64:36
7	**2t**	H	*t*-Bu	74	**4t**:**6t**	100:0

^a^ Reactions conducted a on 0.5-mmol scale based on imine **2**; ^b^ Isolated total yield; ^c^ Ratios were determined by the ^1^H-NMR analysis of the reaction mixture.

## Supplementary Materials

Copies of ^1^H-NMR and ^13^C-NMR spectra of unknown products and ^1^H-NMR spectra of representative crude reaction mixtures are included in the Supporting Information.

## References

[1] Hudhomme P. (2008). Comprehensive Heterocyclic Chemistry III. Four-Membered Rings with One Sulfur and One Nitrogen Atom.

[2] Chanet-Ray J., Vessiere R. (1986). Synthesis and reactions of β-sultams. Org. Prep. Proc. Int..

[3] Otto H.H., Schwenkkraus P. (1982). β-Sutams II: Synthesis of tri-, tetra- and pentamethylene-1,2-thiazetidine 1,1-dioxides. Tetrahedron Lett..

[4] Cavagna F., Koller W., Linkies A., Rehling H., Reuschling D. (1982). Synthese bicyclischer β-sultame. Angew. Chem..

[5] Müller M., Otto H.H. (1991). Properties and reactions of substituted 1,2-thiazetidine 1,1-dioxides: Acylation of β-sultams at C-4, reduction of 4-acyl-β-sultams, and stereochemistry of 4-(α-hydroxyalkyl)-β-sultams. Lieb. Ann. Chem..

[6] Iwama T., Karaoka A., Muraoka O., Tanabe G. (1988). A new synthesis of α-amino acid thioesters by Pummerer reaction of 3-sbstituted-4-sulfinyl-β-sultams. J. Org. Chem..

[7] Iwama T., Karaoka A., Muraoka O., Tanabe G. (1998). Reactions of 1,2-thiazetidine 1,1-dioxides with organometallics: β-Elimination and N–S bond cleavage. Tetrahedron.

[8] Baxter N.J., Laws A.P., Rigoreau L.J.H., Page M.I. (1999). General acid catalysed hydrolysis of β-sultams involves nucleophilic catalysis. Chem. Commun..

[9] Page M.I. (2004). β-Sultams mechanism of reactions and use as inhibitors of serine proteases. Acc. Chem. Res..

[10] Page M.I., Tsang W.Y., Ahmed N. (2006). Comparison of the mechanisms of reactions of β-lactams and β-sultams, including their reactions with some serine enzymes. J. Phys. Org. Chem..

[11] Tsang W.Y., Ahmed N., Hinchliffe P.S., Wood J.M., Harding L.P., Laws A.P., Page. M.I. (2005). Different transition-state structures for the reactions of β-lactams and analogous β-sultams with serine β-lactamases. J. Am. Chem. Soc..

[12] Tsang W.Y., Ahmed N., Harding L.P., Hemming K., Laws A.P., Page M.I. (2005). Acylation versus sulfonylation in the inhibition of elastase by 3-oxo-β-sultams. J. Am. Chem. Soc..

[13] Tsang W.Y., Ahmed N., Hemming K., Page M.I. (2007). Reactivity and selectivity in the inhibition of elastase by 3-oxo-β-sultams and in their hydrolysis. Org. Biomol. Chem..

[14] Enders D., Wallert S., Runsink J. (2003). Asymmetric synthesis of β-amino cyclohexyl sulfonates, β-sultams and γ-sultones. Synthesis.

[15] Paquette L.A., Barton W.R.S., Gallucci. J.C. (2004). Synthesis of 1-aza-8-thiabicyclo[4.2.1]nona-2,4-diene 8,8-dioxide and its conversion to a strained spirocycle via photoinduced SO_2_−N bond cleavage. Org. Lett..

[16] Baldoli C., Del B.P., Perdicchia D., Pilati T. (1999). Stereoselective synthesis of β-sultams using chiral tricarbonyl(η6-arene)chromium(0) complexes. Tetrahedron.

[17] De Lewis A.K., Mok B.J., Tocher D.A., Wilden J.D., Caddick S. (2006). New synthesis of β-sultams from pentafluorophenyl sulfonates. Org. Lett..

[18] Staudinger H., Pfenninger E. (1916). Über die einwirkung von schwefeldioxyd auf diphenyl-diazomethan. Chem. Ber..

[19] Szymonifka M.J., Heck J.V. (1989). The synthesis and reactions of 4-carbomethoxy β-sultams. Tetrahedron Lett..

[20] Grunder E., Leclerc G. (1989). Synthesis of (±)-erythro o- and (±)-threo-1.2-diphenyltaurines by diasteroselective hydrolysis of thiazetidine 1,1-dioxides. Synthesis.

[21] Grunder-Klotz E., Ehrhardt J.-D. (1991). The 3,4-dimethoxybenzyl moiety as a new N-protecting group of 1,2-thiazetidine 1,1-dioxides. Tetrahedron Lett..

[22] Gordeev M.F., Gordon E.M., Patel D.V. (1997). Solid-phase synthesis of β-sultams. J. Org. Chem..

[23] Zajac M., Peters R. (2007). Catalytic asymmetric formation of β-sultams. Org. Lett..

[24] Zajac M., Peters R. (2009). Catalytic asymmetric synthesis of β-sultams as precursors for taurine derivatives. Chem. Eur. J..

[25] Liu J., Hou S.L., Xu J.X. (2011). Diverse reactions of sulfonyl chlorides and cyclic imines. Phosphorus Sulfur Silicon Relat. Elem..

[26] Yang Z.H., Xu. J.X. (2014). Insights into the β-sultam ring formation in the sulfa-Staudinger cycloadditions. J. Org. Chem..

[27] Yang Z.H., Chen N., Xu. J.X. (2015). Substituent-controlled annuloselectivity and stereoselectivity in the sulfa-Staudinger cycloadditions. J. Org. Chem..

[28] Yang Z.H., Xu J.X. (2015). Annuloselectivity and stereochemistry in the sulfa-Staudinger cycloadditions of cyclic imines. RSC Adv..

[29] Yang Z.H., Xu J.X. (2015). Substituent effect on the diastereoselectivity in the sulfa-Staudinger cycloaddition. Tetrahedron.

[30] Wu Q.Y., Yang Z.H., Xu J.X. (2016). Temperature-dependent annuloselectivity and stereochemistry in the reactions of methanesulfonyl sulfene with imines. Org. Biomol. Chem..

[31] Yang Z.H., Li S.Q., Zhang Z., Xu J.X. (2014). Base-switched annuloselectivity in the reactions of ethyl malonyl chloride and imines. Org. Biomol. Chem..

[32] Clark L.W. (1958). The effect of aromatic nitro compounds on malonic acid. J. Phys. Chem..

[33] Terrier F., Xie H.-Q., Farrel P.G. (1990). The effect of nitro-substitution upon diphenylmethane reactivity. J. Org. Chem..

[34] Font-Sanchis E., Aliaga C., Cornejo R., Scaiano J.C. (2003). Reactivity toward oxygen of isobenzofuranyl radicals: Effect of nitro group substitution. Org. Lett..

[35] Franck R.W., Williamson S.M.A. (1966). The shielding effect of the nitro group. J. Org. Chem..

[B36-molecules-22-00784] 36.The *cis*- and *trans*-configurations of β-sultams or β-lactams are decided by the coupling constants between the protons at C3 and C4 positions. Generally, *J* = 5–7 Hz for *trans*-β-sultams, and *J* = 8–9 Hz for *cis*-β-sultams; *J* = 4–6 Hz for *cis*-β-lactams, and *J* = 0–3 Hz for *trans*-β-lactams.

[37] Jiao L., Liang Y., Xu J.X. (2006). Origin of the Relative Stereoselectivity of the β-Lactam Formation in the Staudinger Reaction. J. Am. Chem. Soc..

[38] Tsuge O., Iwanami S. (1970). Cycloaddition reactions of benzoylsulfene with anil. Bull. Chem. Soc. Jpn..

[39] Zheng Y.P., Xu J.X. (2014). Thorpe-Ingold effect and its application in cyclizations in organic chemistry. Prog. Chem..

[40] Jung M.E., Piizzi G. (2005). *gem*-Disubstituent effect: Theoretical basis and synthetic applications. Chem. Rev..

[41] Cossio F.P., Arrieta A., Sierra M.A. (2008). The mechanism of the ketene-imine (Staudinger) reaction in its centennial: Still an unsolved problem?. Acc. Chem. Res..

[42] Liang Y., Jiao L., Zhang S.W., Xu J.X. (2005). Microwave- and photoirradiation-induced Staudinger reactions of cyclic imines and ketenes generated from α-diazoketones. A further investigation into the stereochemical process. J. Org. Chem..

[43] Li B.N., Wang Y.K., Du D.-M., Xu J.X. (2007). Notable and obvious ketene substituent-dependent effect of temperature on the stereoselectivity in the Staudinger reaction. J. Org. Chem..

[44] Wang Y.K., Liang Y., Jiao L., Du D.M., Xu J.X. (2006). Do reaction conditions affect the stereoselectivity in the Staudinger reaction?. J. Org. Chem..

[45] Qi H.Z., Li X.Y., Xu J.X. (2011). Stereoselective control in the Staudinger reactions involving monosubstituted ketenes with electron acceptor substituents: experimental investigation and theoretical rationalization. Org. Biomol. Chem..

[46] Wang Z.X., Chen N., Xu J.X. (2011). Selectivities in the reaction of vicinal diimines and acyl chlorides. Tetrahedron.

[47] Hu L.B., Wang Y.K., Li B.N., Du D.M., Xu J.X. (2007). Diastereoselectivity in the Staudinger reaction: A useful probe for investigation of nonthermal microwave effects. Tetrahedron.

[48] Yang Z.H., Xu J.X. (2012). Remarkable ketene substituent dependent effect of photo irradiation on the diastereoselectivity in the Staudinger reaction. Tetrahedron Lett..

[49] Jiao L., Zhang Q.F., Liang Y., Zhang S.W., Xu J.X. (2006). A versatile method for the synthesis of 3-alkoxycarbonyl β-lactam derivatives. J. Org. Chem..

