# Supplementary material for: Ortho-Nitro Effect on the Diastereoselective Control in Sulfa-Staudinger and Staudinger Cycloadditions
Source: Molecules. 2017 May 12;22(5):784. doi: 10.3390/molecules22050784 (PMC6154622; doi:10.3390/molecules22050784)
Supplement: Supplementary file 1 [file molecules-22-00784-s001.pdf]

Supporting Information

Ortho-Nitro Effect on the Diastereoselective Control in Sulfa-Staudinger and Staudinger Cycloadditions

Zhanhui Yang, Hassane Abdellaoui, Wei He, and Jiaxi Xu*

State Key Laboratory of Chemical Resource Engineering, Department of Organic Chemistry, Faculty of Science, Beijing University of Chemical Technology, Beijing 100029, People's Republic of China

Email: jxxu@mail.buct.edu.cn

Contents

1. Copies of ^{1}H and ^{13}C NMR spectra of unknown products S2

2. Copies of representative ^{1}H spectra of reaction mixtures.................. S26

---

* Corresponding author. Tel.: +8610-6443-5565; fax: +8610-6443-5565; e-mail: jxxu@mail.buct.edu

1. Copies of ^{1}H and ^{13}C NMR spectra of unknown products

Ethyl trans-3-(2-nitrophenyl)-2-propyl-1,2-thiazetidine-4-carboxylate 1,1-dioxide **(*trans*-3d)**
This product was isolated as a mixture of *cis*-**5d** (*trans/cis* = 86:14)

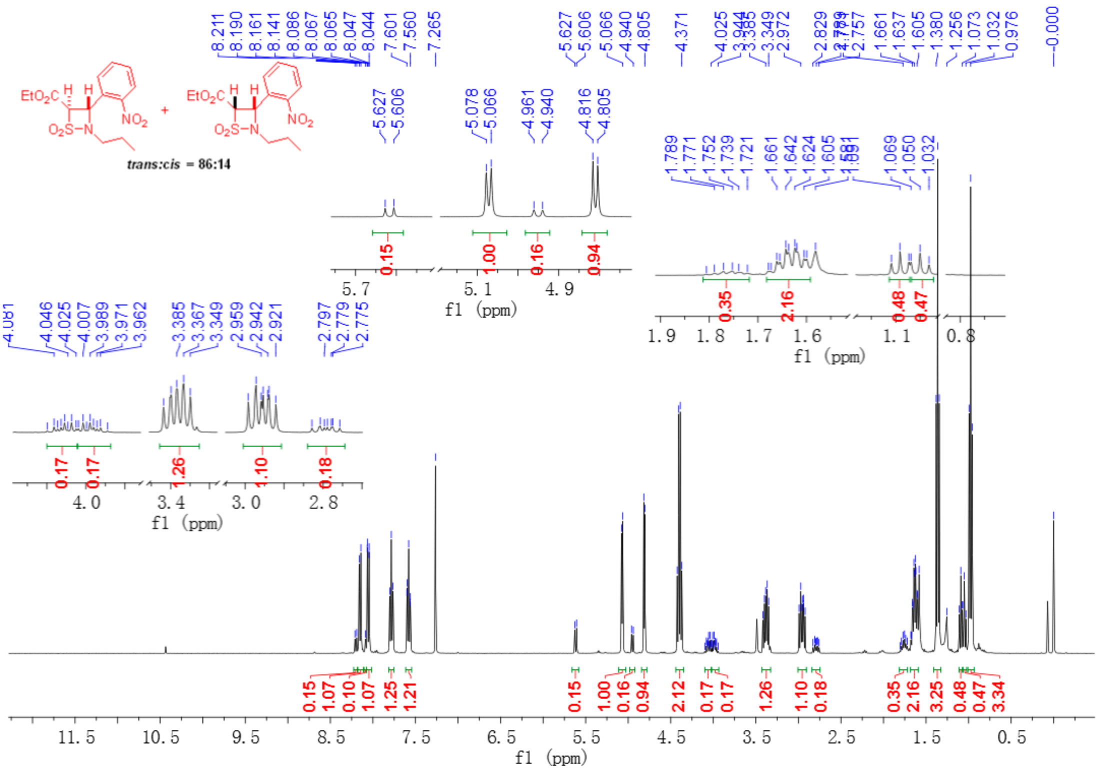

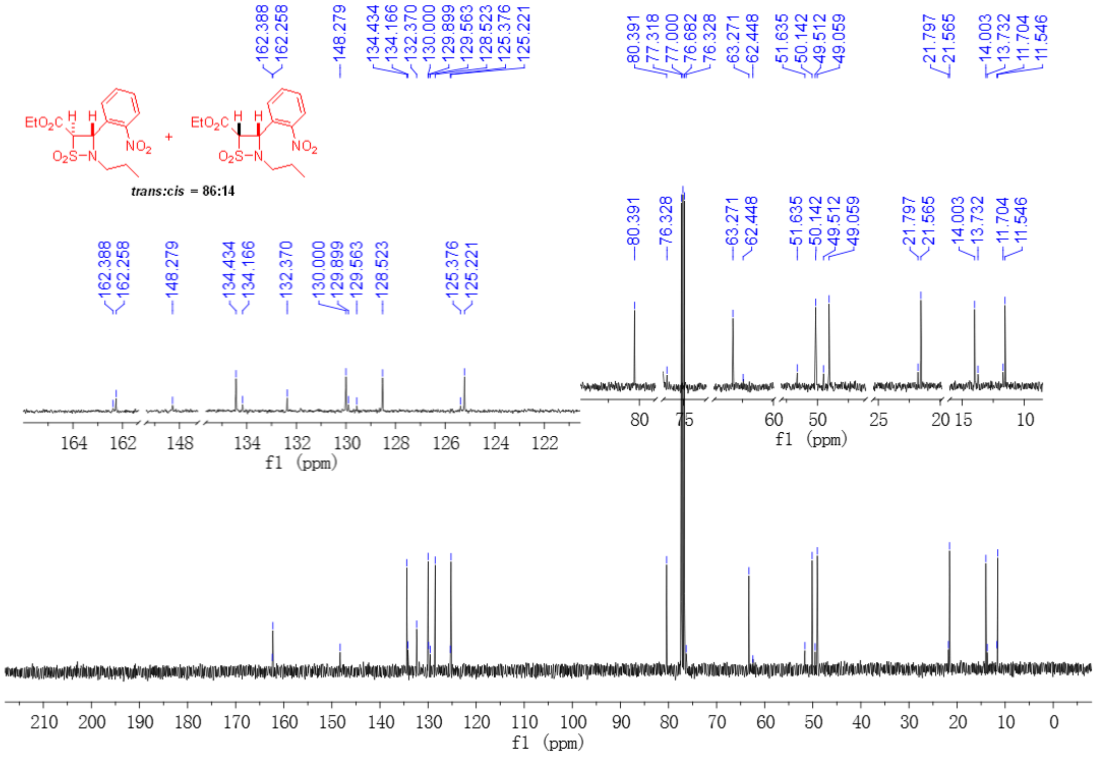

Ethyl trans-2-benzyl-3-(2-nitrophenyl)-1,2-thiazetidine-4-carboxylate 1,1-dioxide **(3e)**

Compounds *trans*-**3e** and *cis*-**5e** were isolated as a mixture, with a *trans/cis* ratio of 88:12.

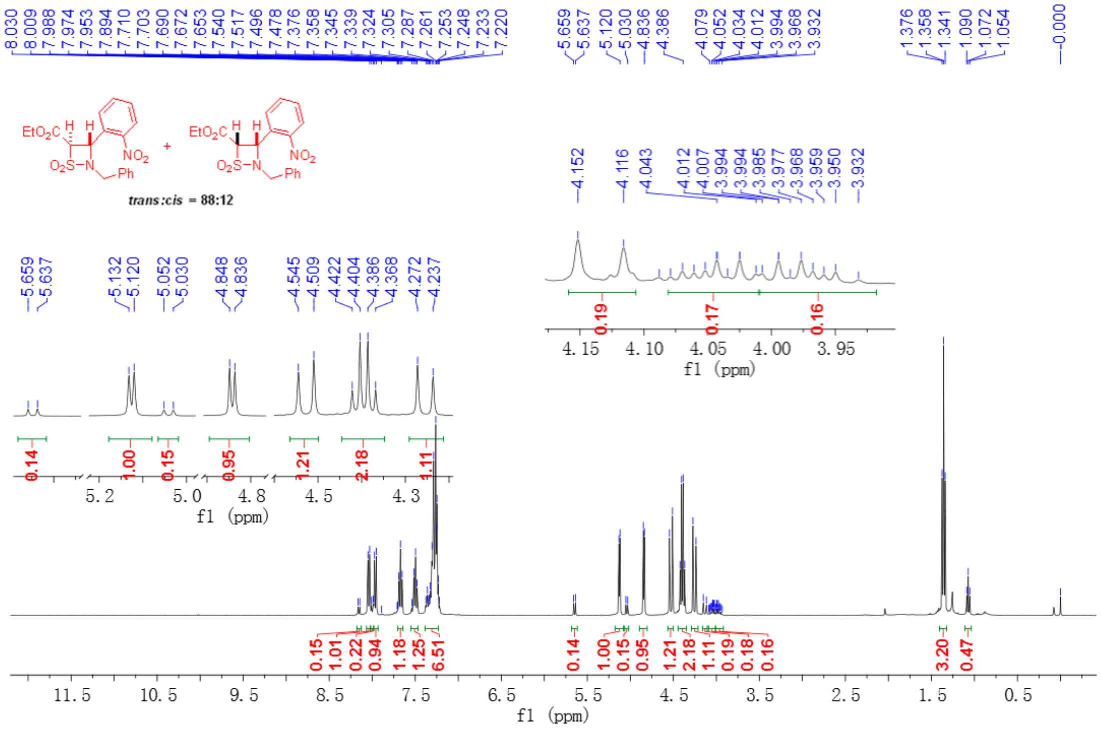

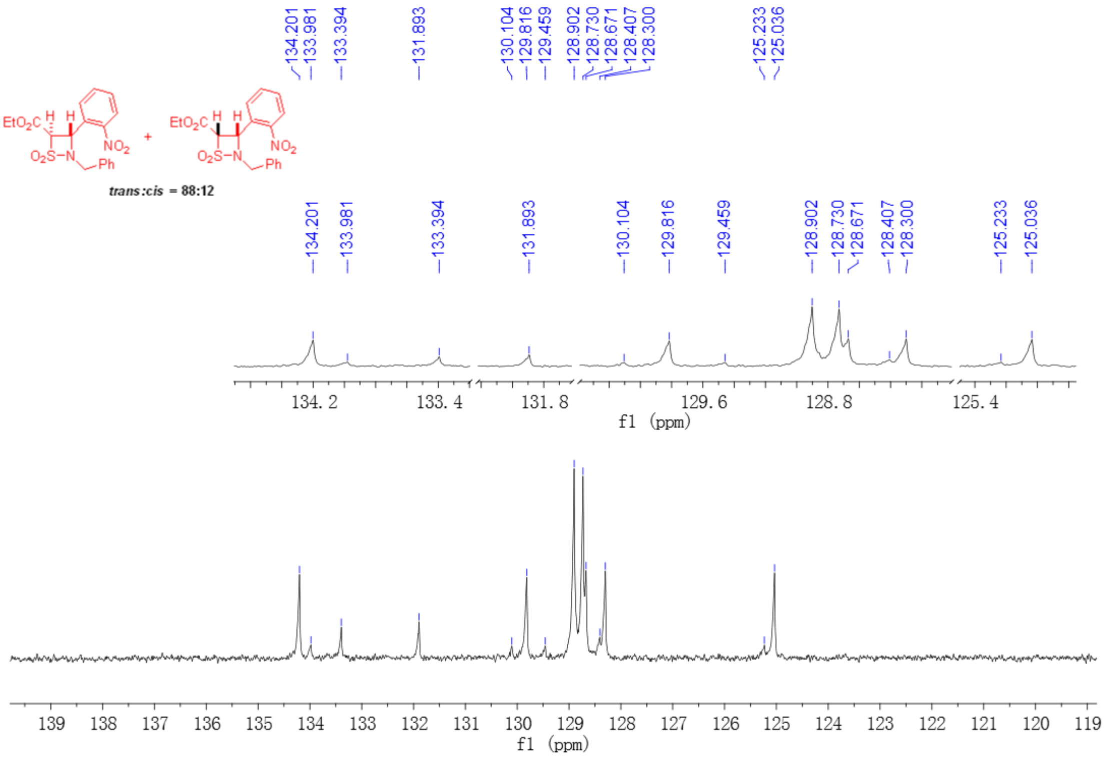

Ethyl trans-2-(tert-butyl)-3-(2-nitrophenyl)-1,2-thiazetidine-4-carboxylate 1,1-dioxide **(3f)**

Compounds *trans*-**3f** and *cis*-**5f** were isolated as a mixture, with a *trans/cis* ratio of 97:3.

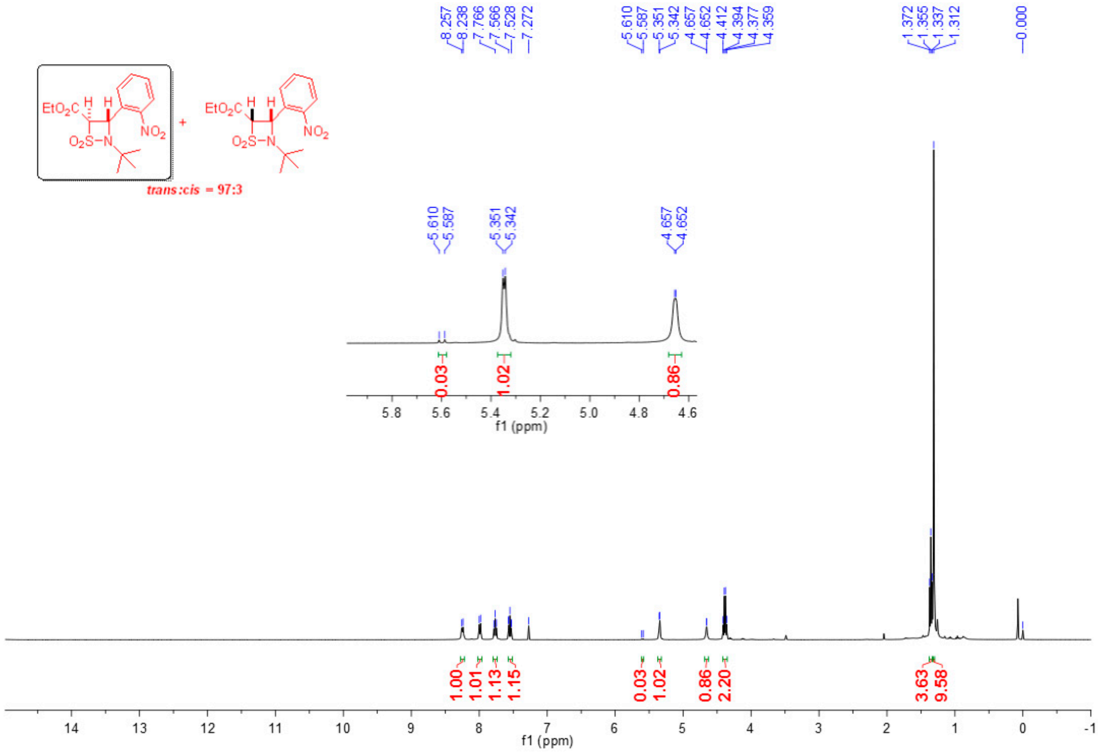

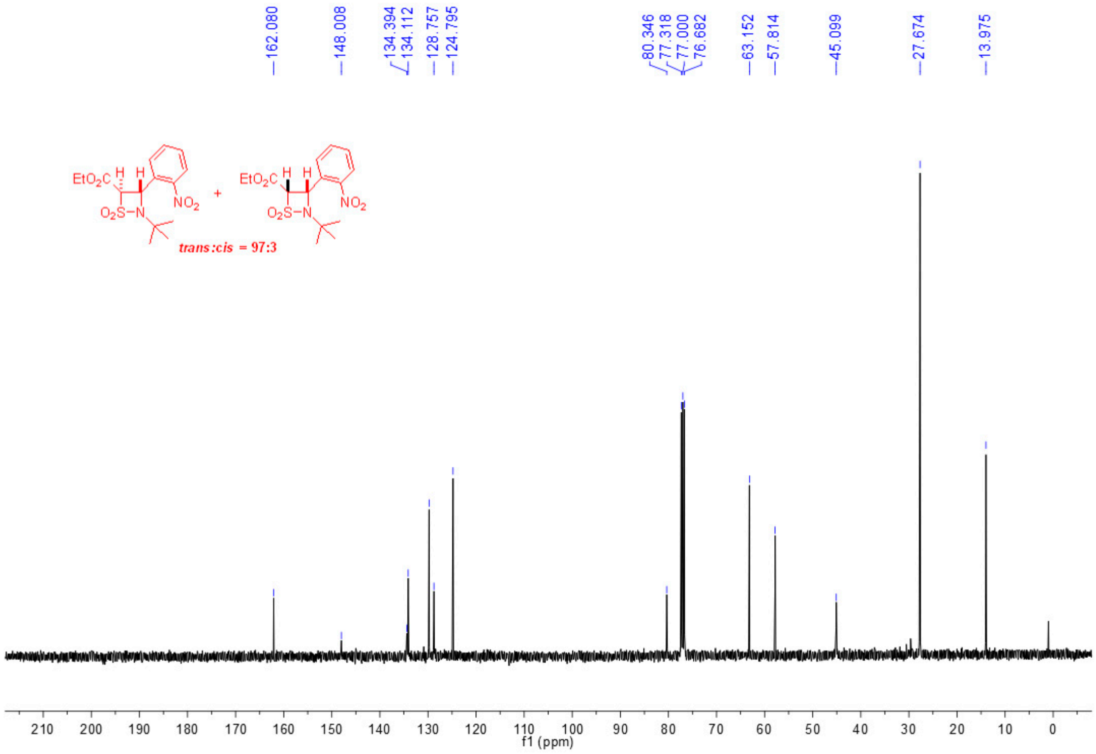

trans-2-Methyl-3-(3-nitrophenyl)-4-phenyl-1,2-thiazetidine 1,1-dioxide **(3bc)**

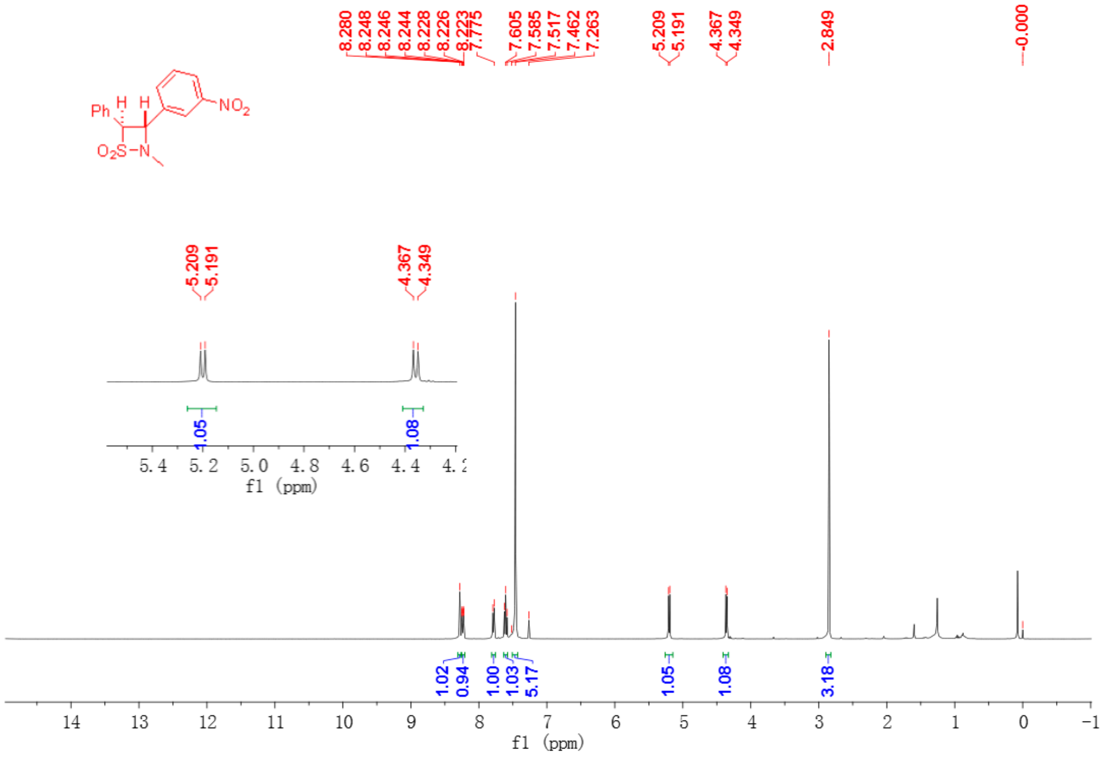

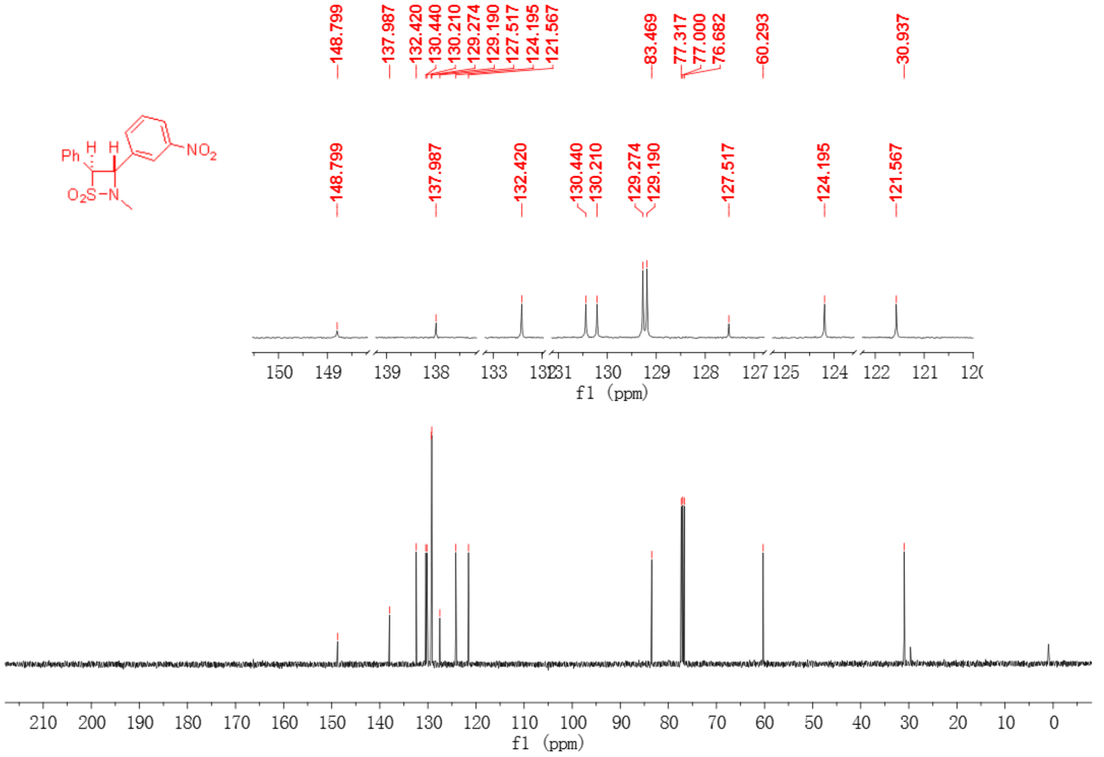

Cis-2-methyl-3-(3-nitrophenyl)-4-phenyl-1,2-thiazetidine 1,1-dioxide (*cis*-**5bc)**

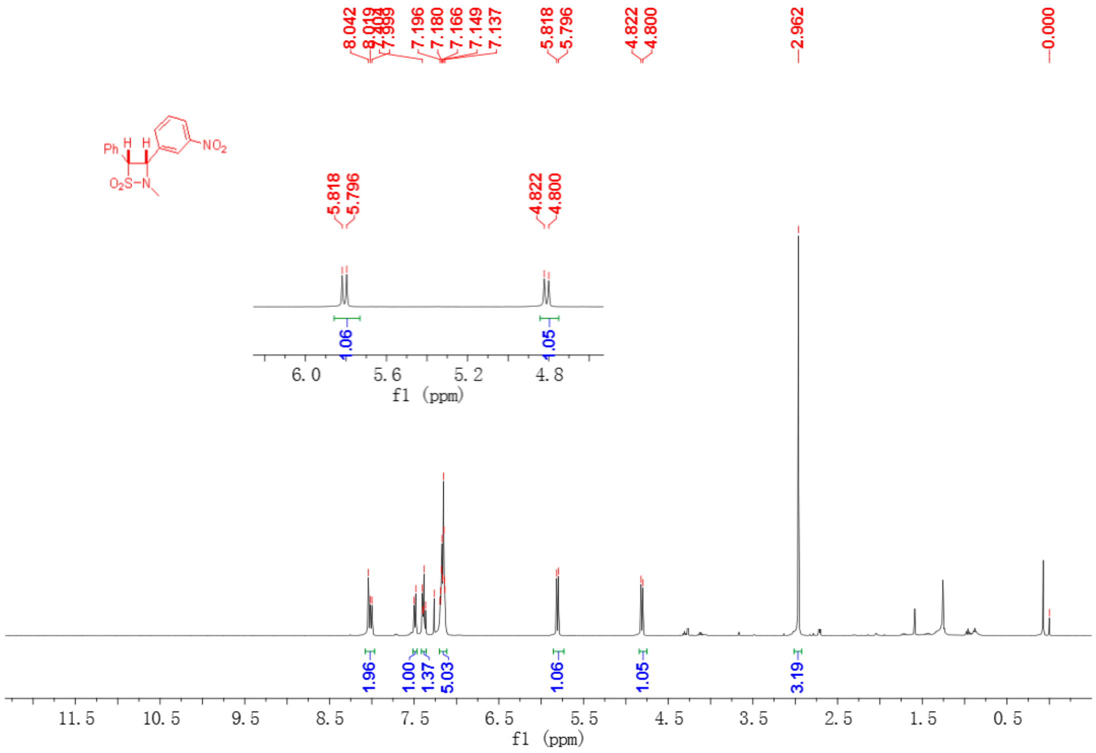

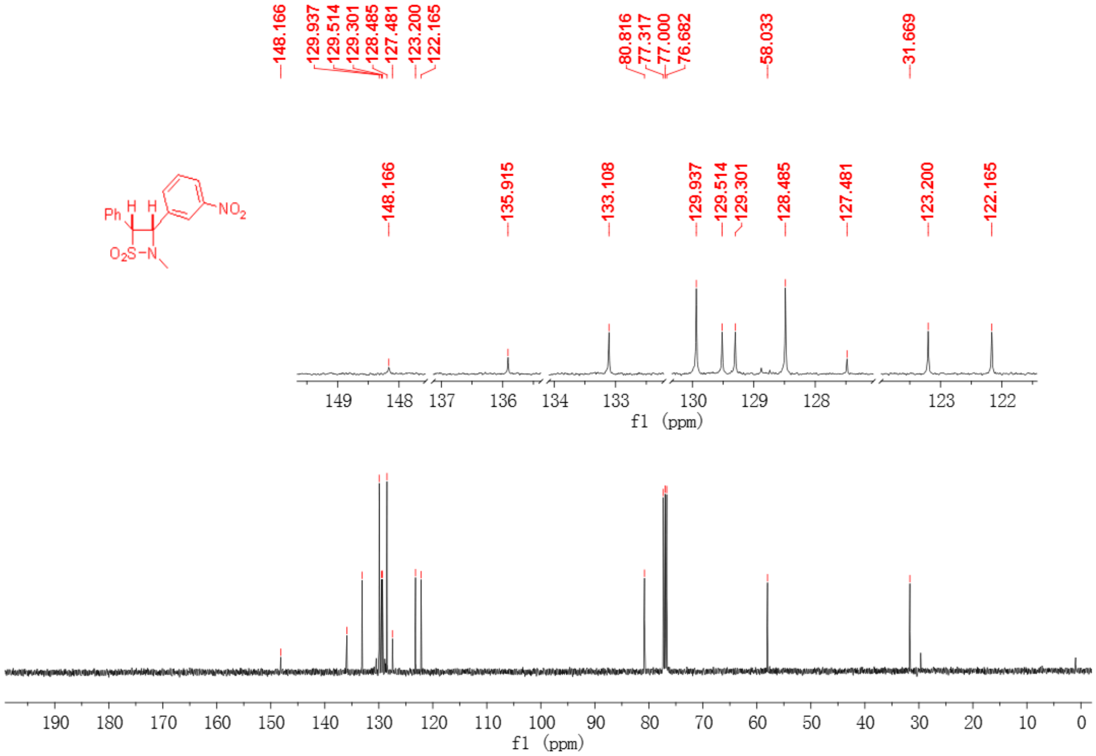

trans and cis-2-Methyl-3-(2-nitrophenyl)-4-phenyl-1,2-thiazetidine 1,1-dioxides **(3ac** and **5ac)**
Isolated as a mixture, trans/cis = 52:48.

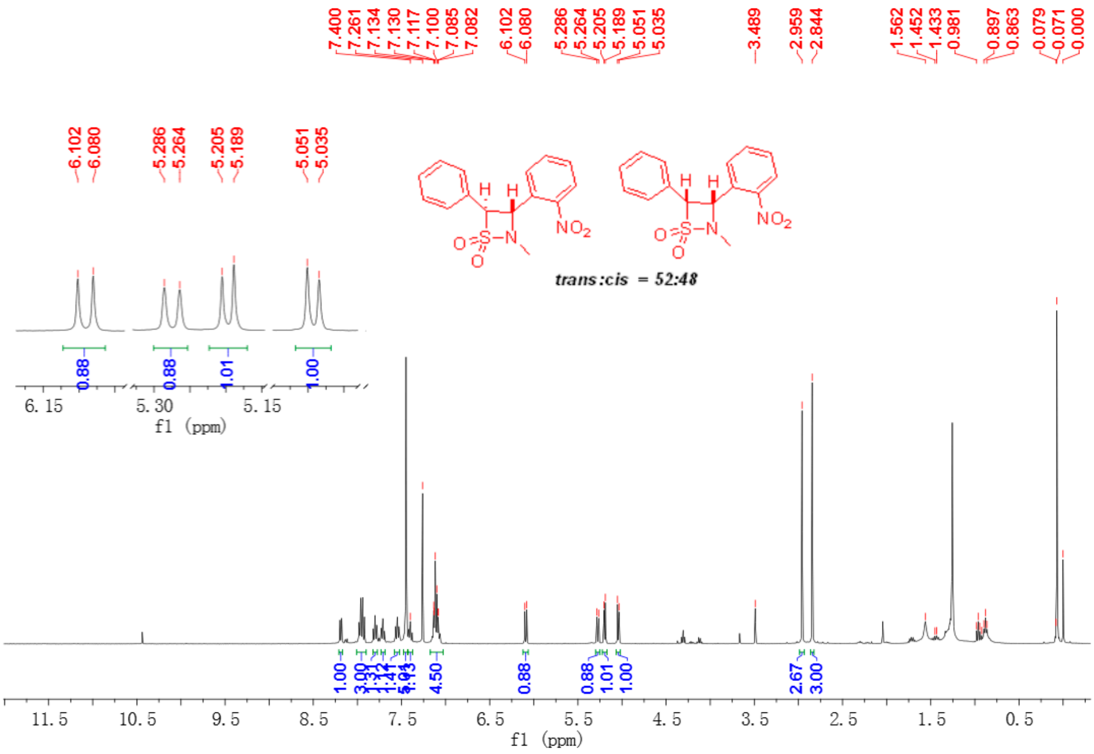

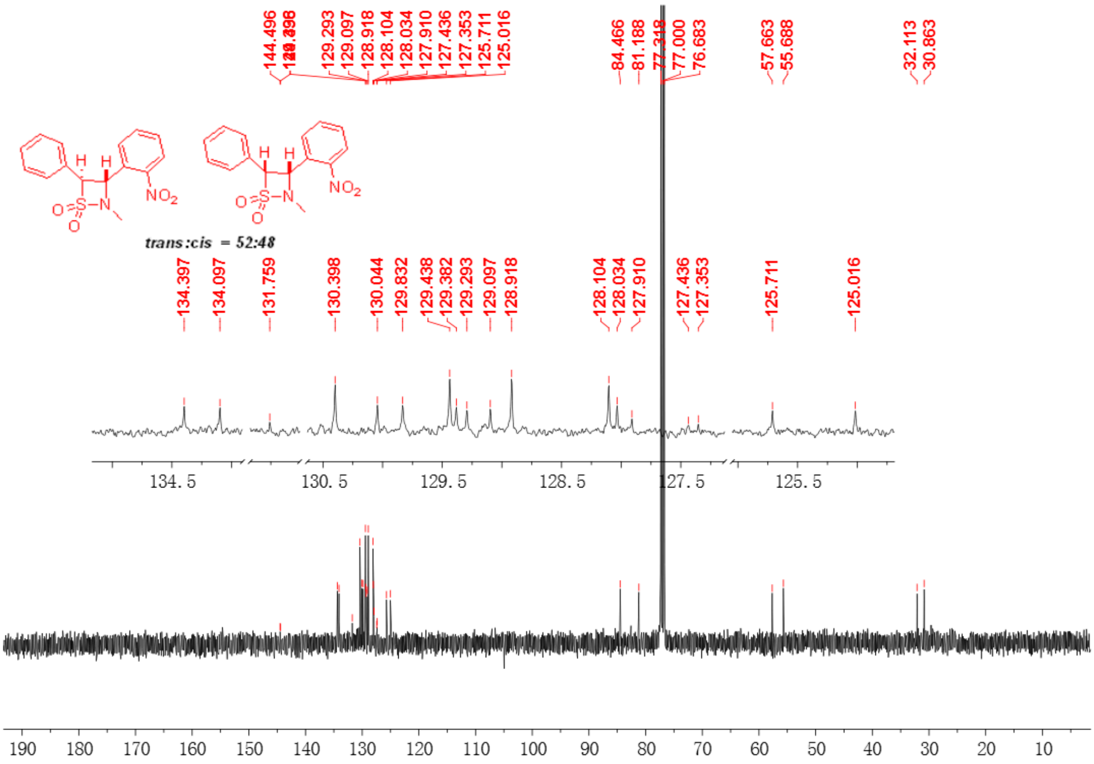

***Ethyl trans-2-propyl-3-(o-tolyl)-1,2-thiazetidine-4-carboxylate 1,1-dioxide* (3g)**

Compounds *trans*-**3g** and *cis*-**5g** were isolated as a mixture, with a *trans/cis* ratio of 99:1.

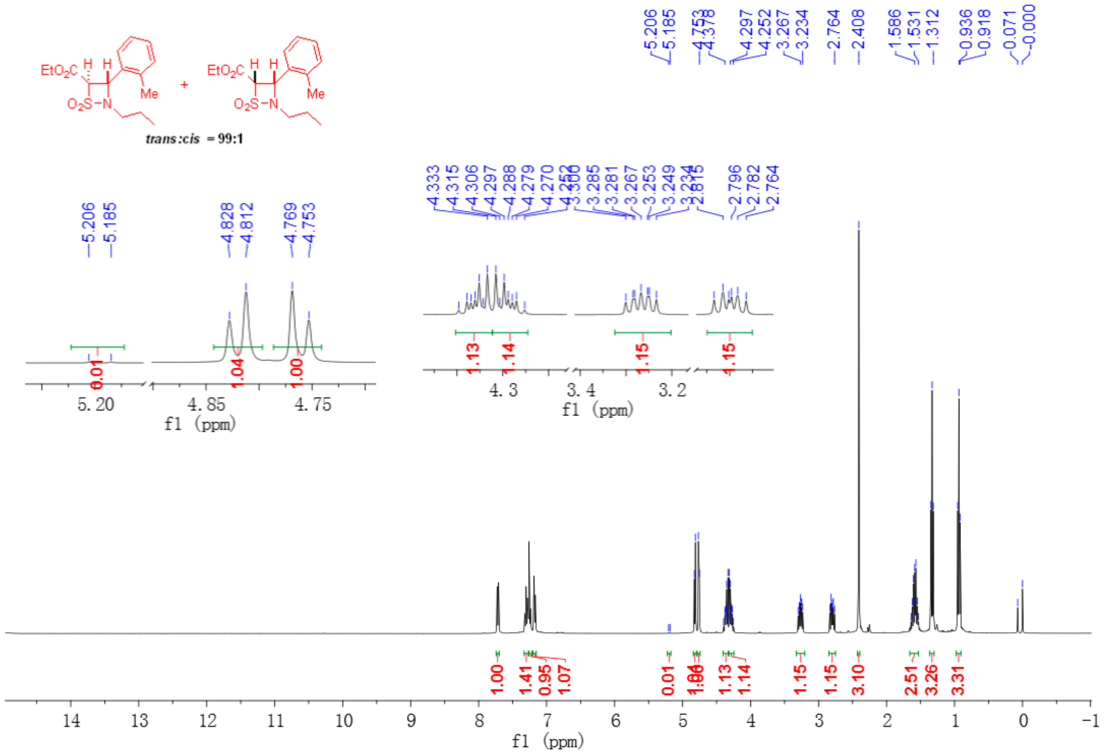

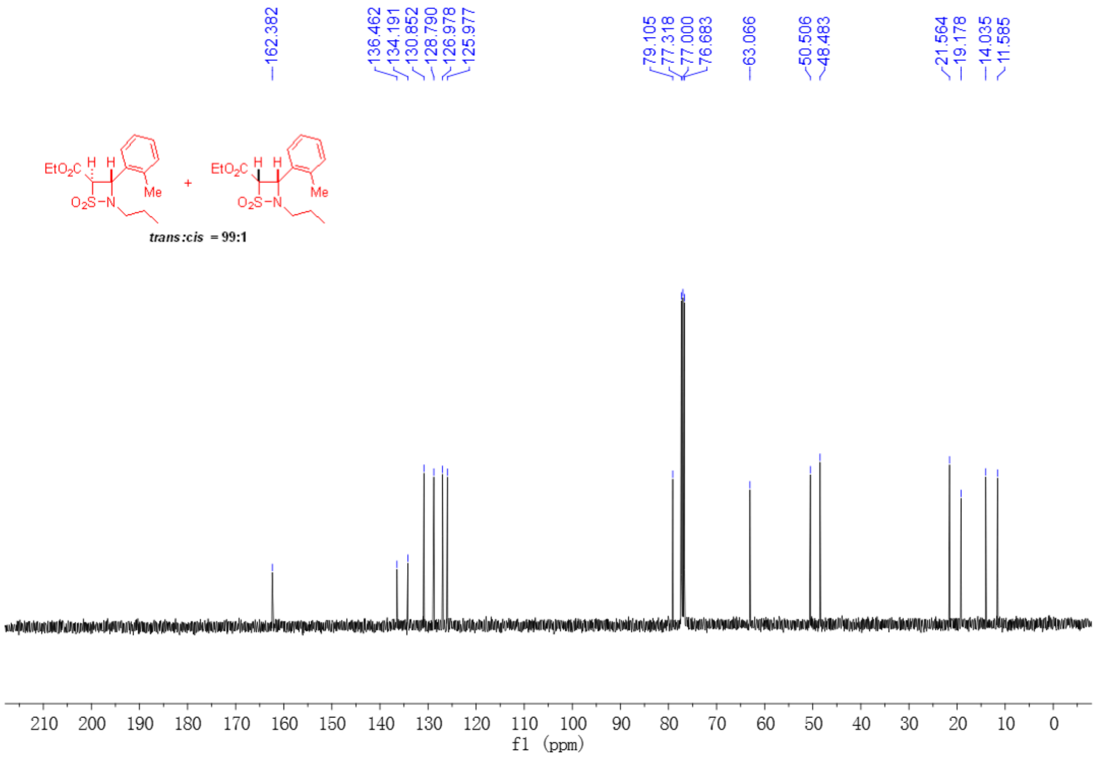

Ethyl trans-2-propyl-3-(2-(trifluoromethyl)phenyl)-1,2-thiazetidine-4-carboxylate 1,1-dioxide **(3h)**

Compounds *trans*-**3h** and *cis*-**5h** were isolated as a mixture, with a *trans/cis* ratio of 97:3.

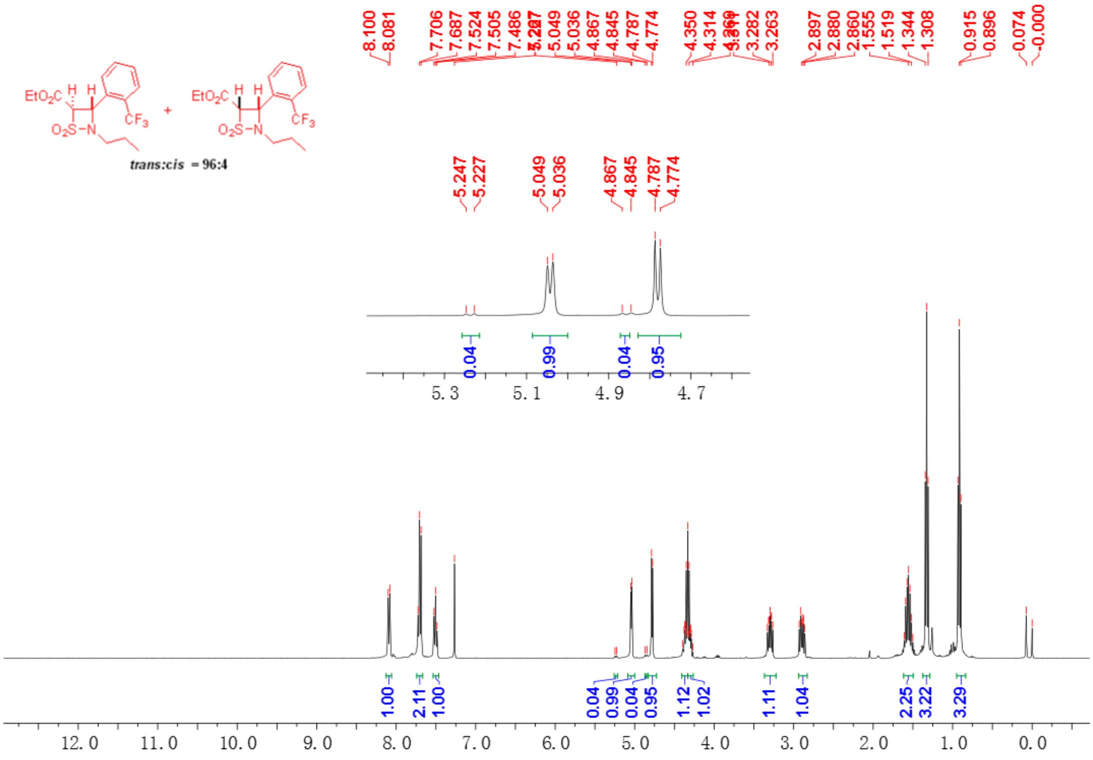

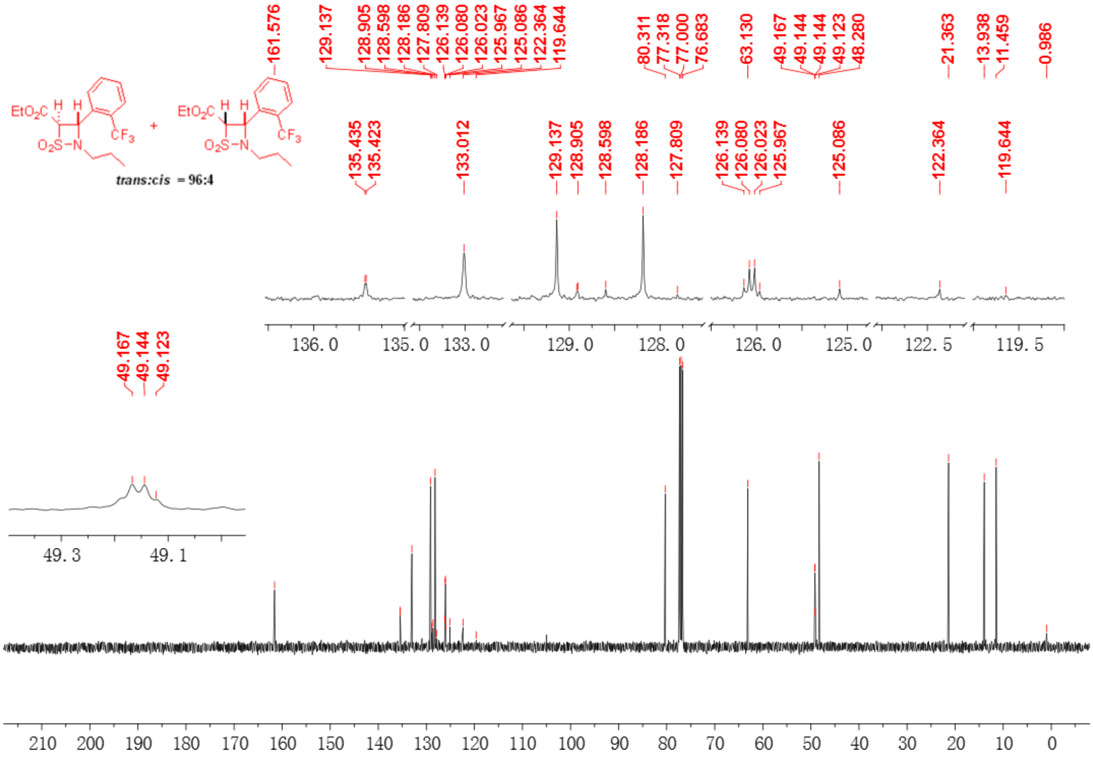

Ethyl trans-3-(2-methoxyphenyl)-2-propyl-1,2-thiazetidine-4-carboxylate 1,1-dioxide **(3i)**

Compounds *trans*-**3i** and *cis*-**5i** were isolated as a mixture, with a *trans/cis* ratio of 99:1.

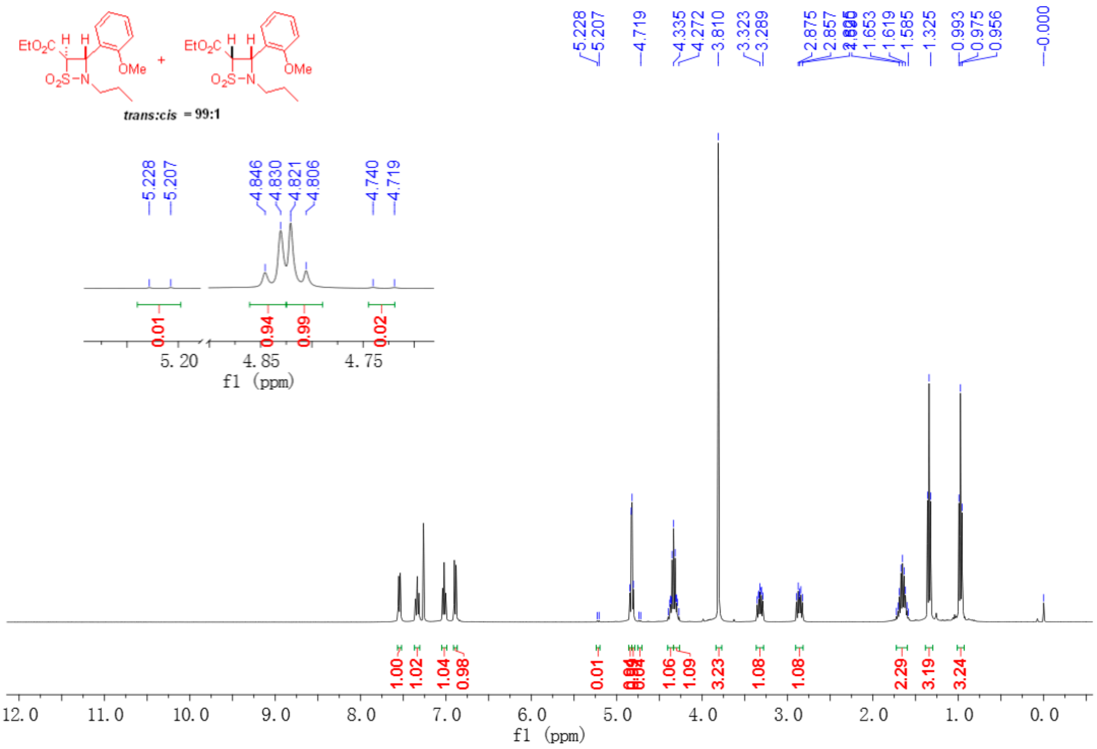

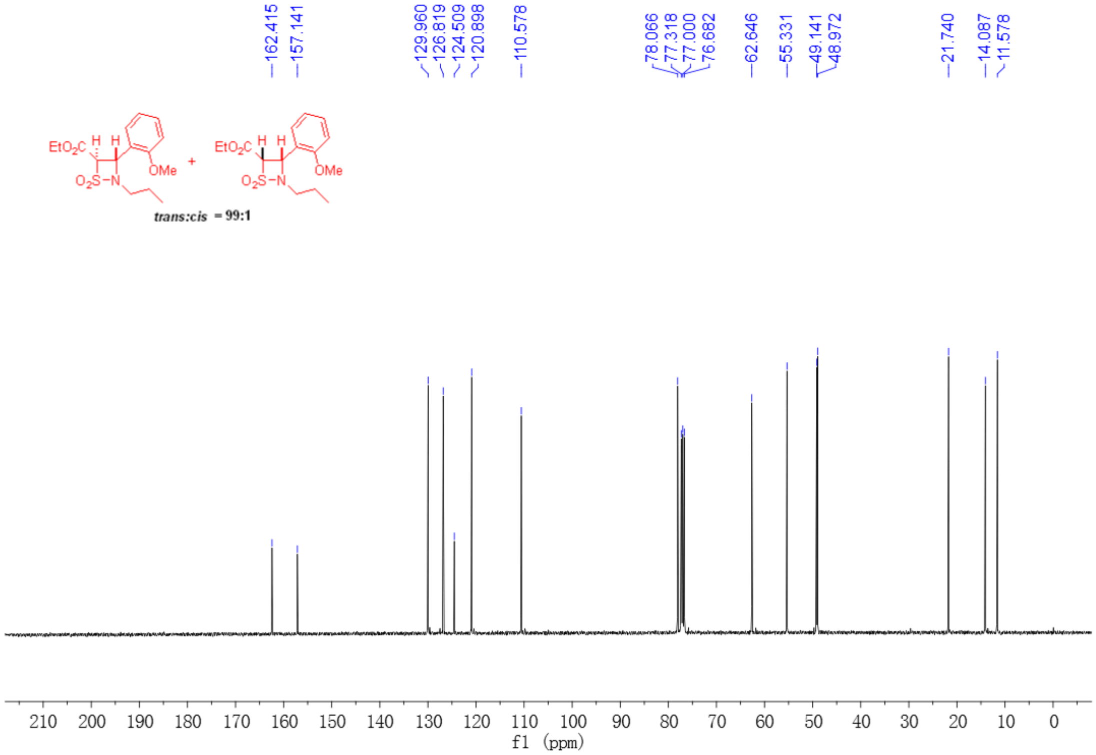

Ethyl trans-3-(2-fluorophenyl)-2-propyl-1,2-thiazetidine-4-carboxylate 1,1-dioxide **(3j)**
Compounds *trans*-**3j** and *cis*-**5j** were isolated as a mixture, with a *trans/cis* ratio of 97:3.

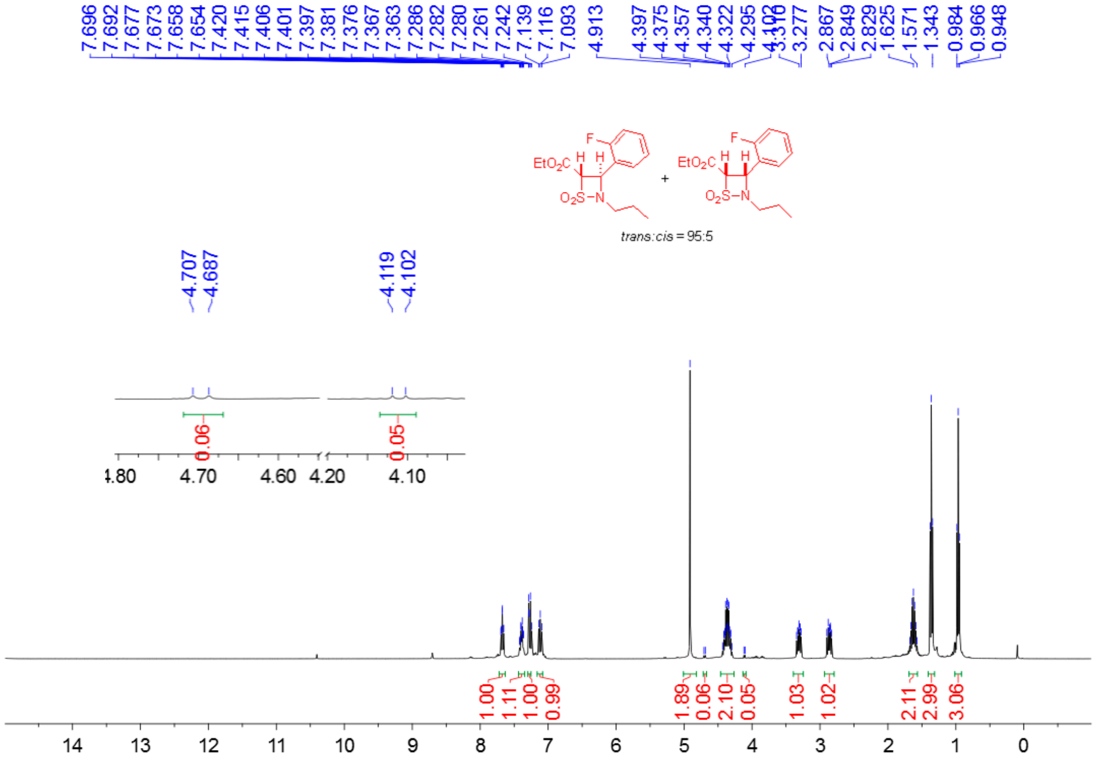

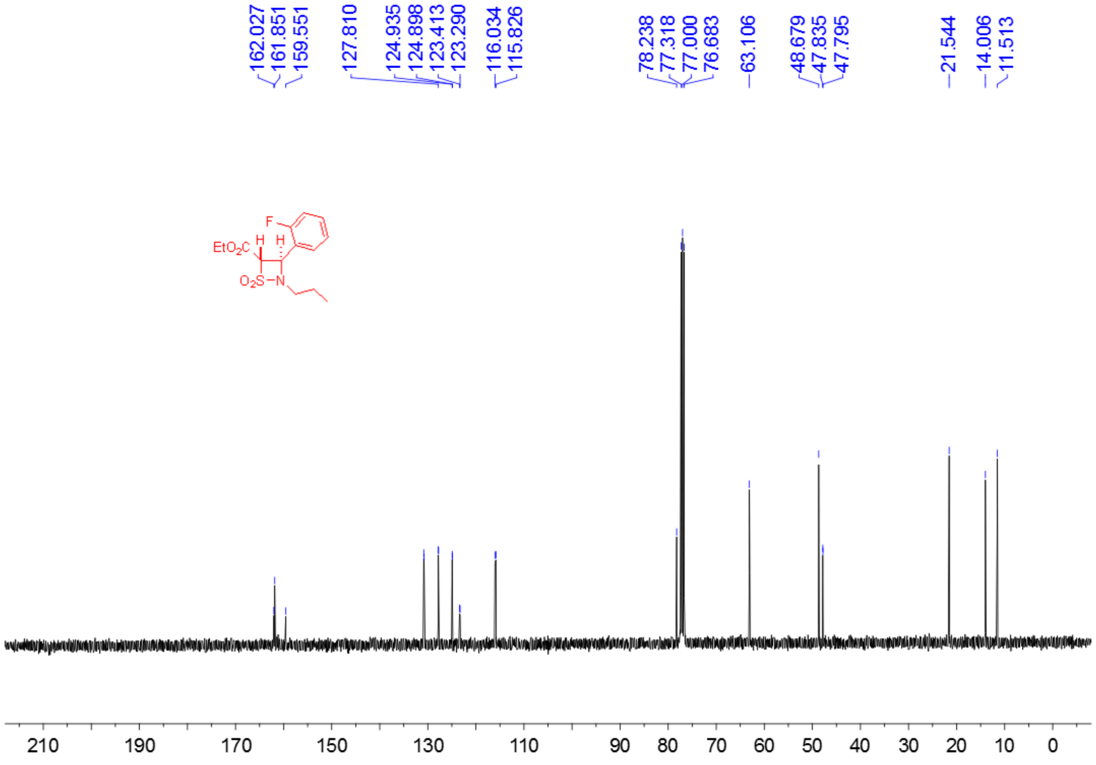

Ethyl trans-3-(2-chlorophenyl)-2-propyl-1,2-thiazetidine-4-carboxylate 1,1-dioxide **(3k)**

Compounds *trans*-**3j** and *cis*-**5j** were isolated as a mixture, with a *trans/cis* ratio of 97:3.

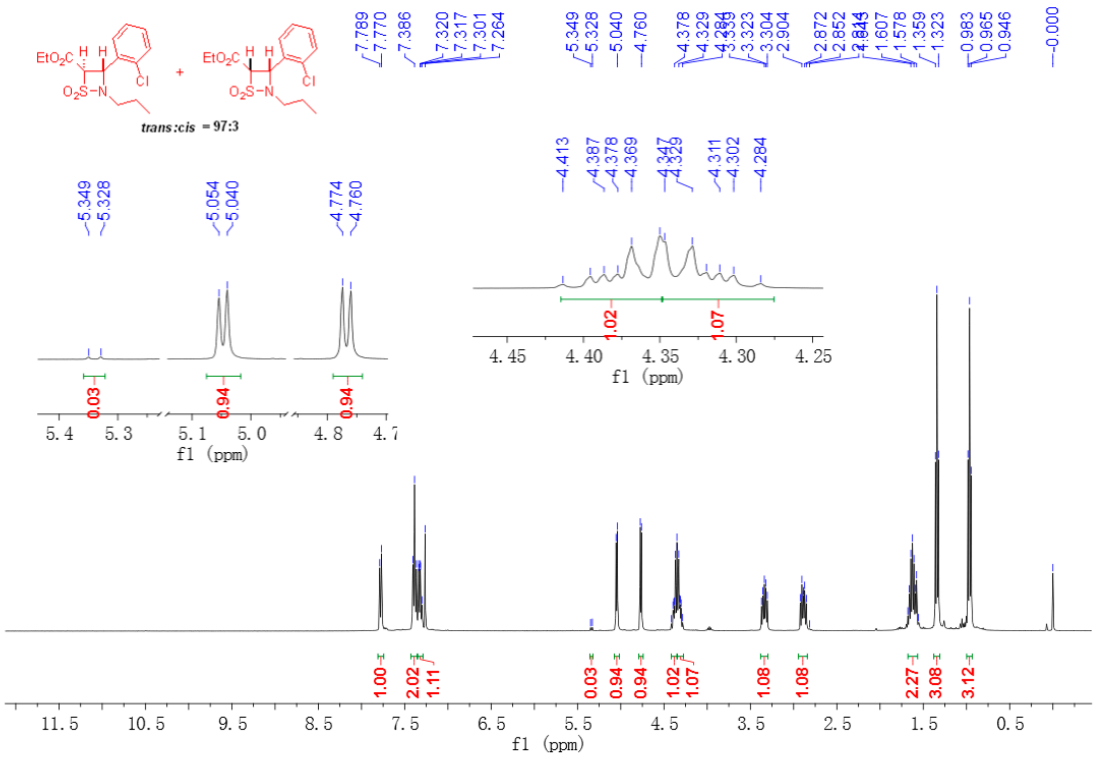

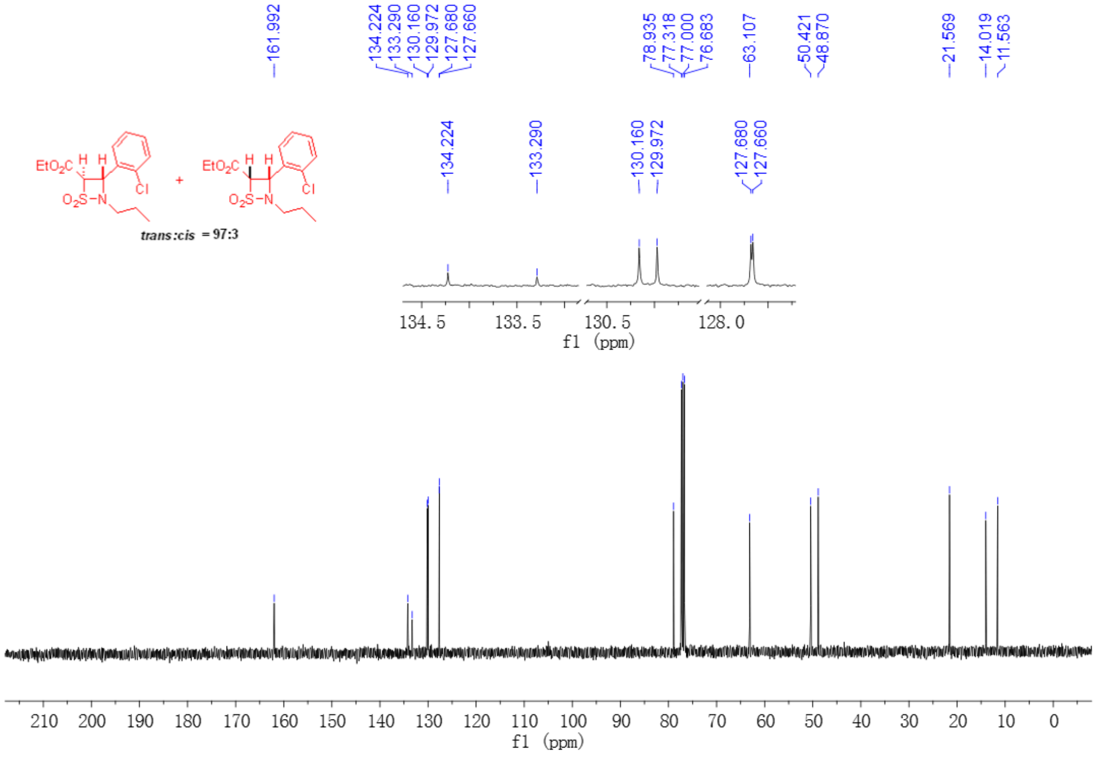

Ethyl trans-3-(2-bromophenyl)-2-propyl-1,2-thiazetidine-4-carboxylate 1,1-dioxide **(3l)**

Compounds *trans*-**3k** and *cis*-**5k** were isolated as a mixture, with a *trans/cis* ratio as 97:3.

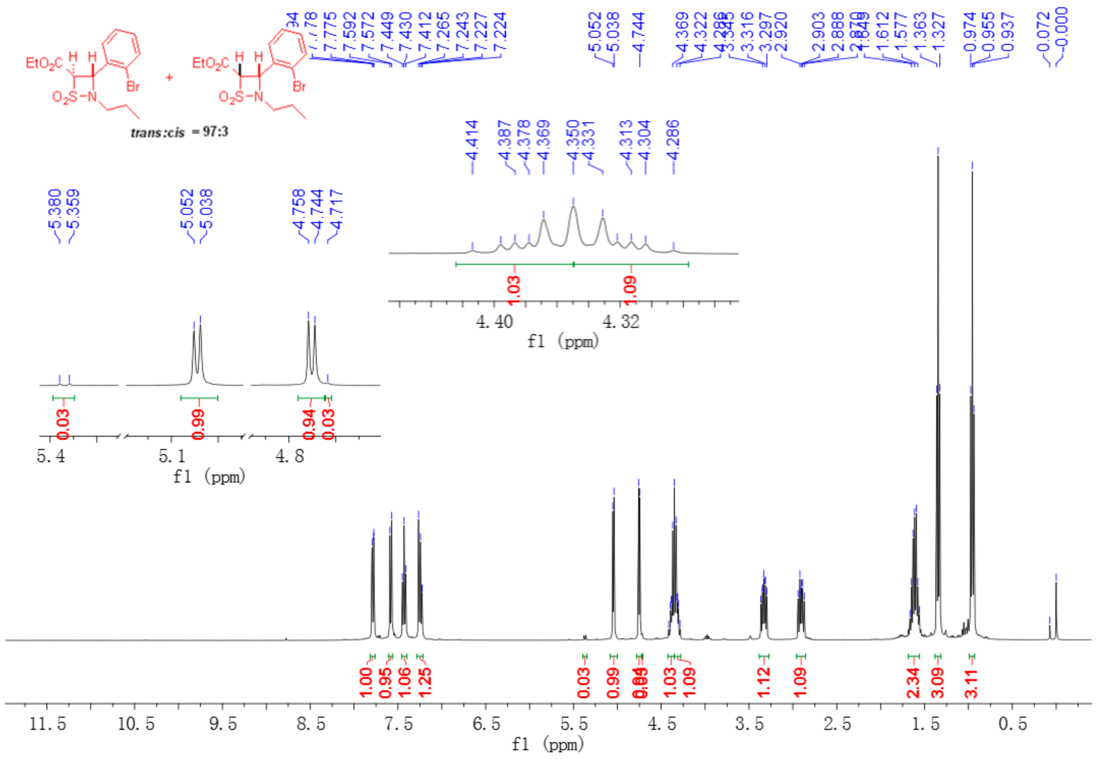

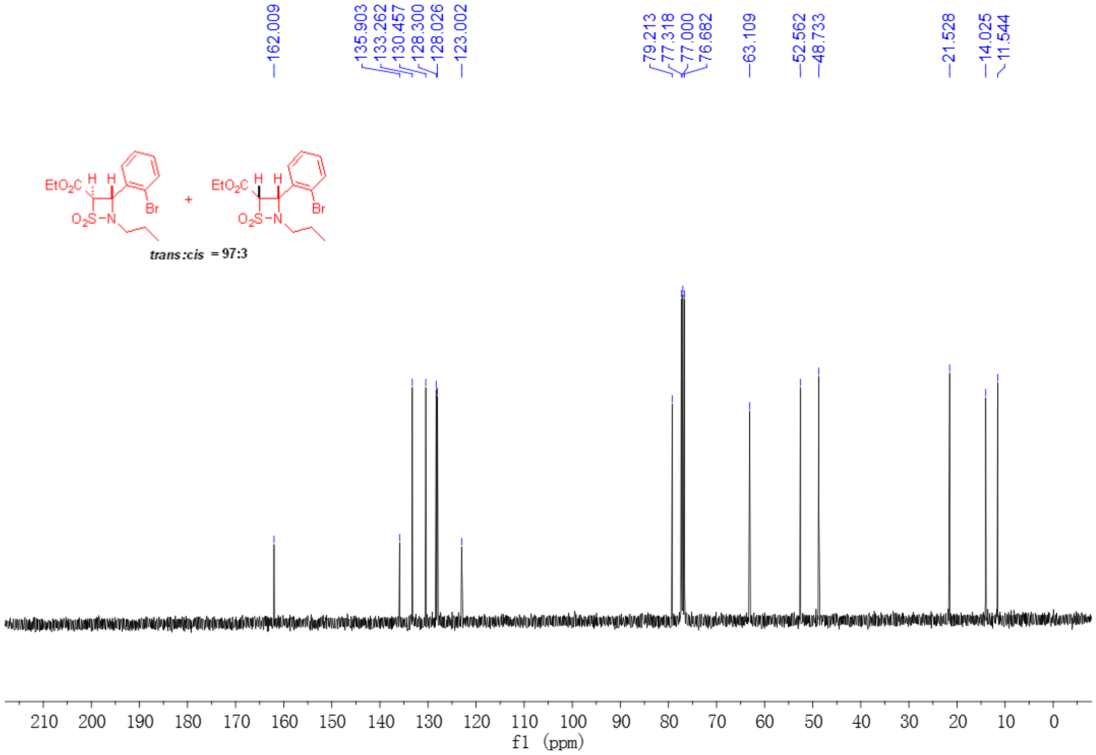

Ethyl trans-3-(2,6-dichlorophenyl)-2-propyl-1,2-thiazetidine-4-carboxylate 1,1-dioxide **(3m)**

Compounds *trans*-**3l** and *cis*-**5l** were isolated as a mixture, with a *trans/cis* ratio of 99:1.

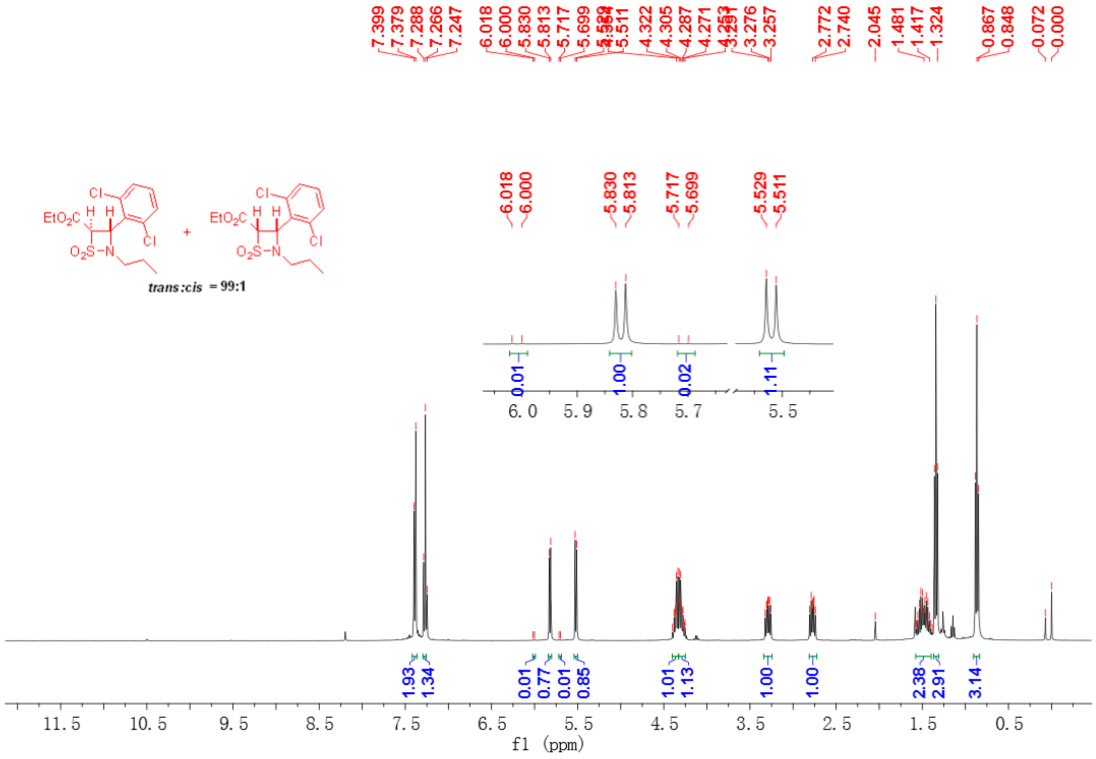

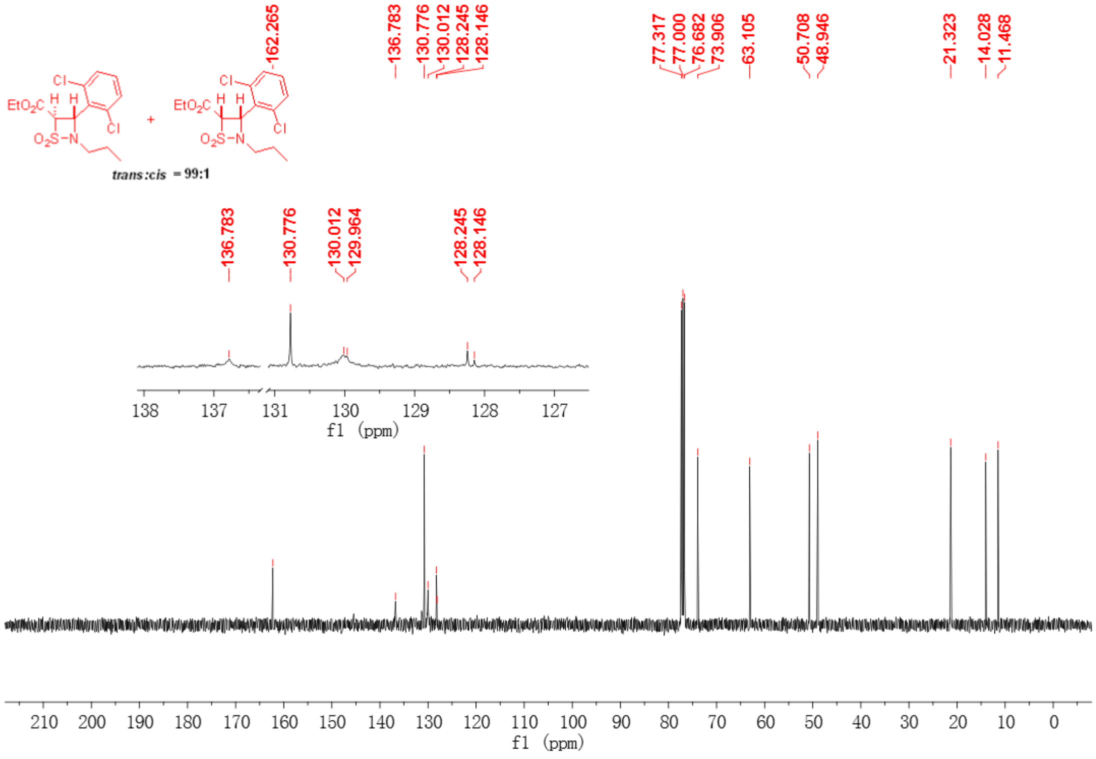

Ethyl trans-3-([1,1'-biphenyl]-2-yl)-2-propyl-1,2-thiazetidine-4-carboxylate 1,1-dioxide **(3n)**

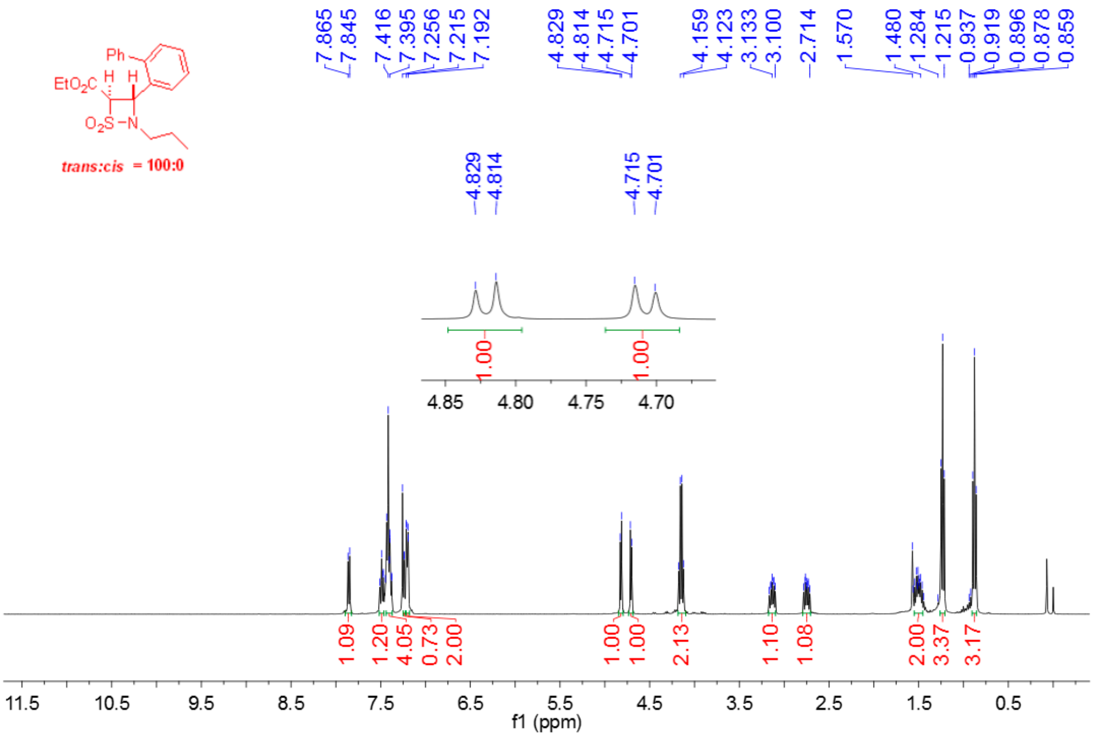

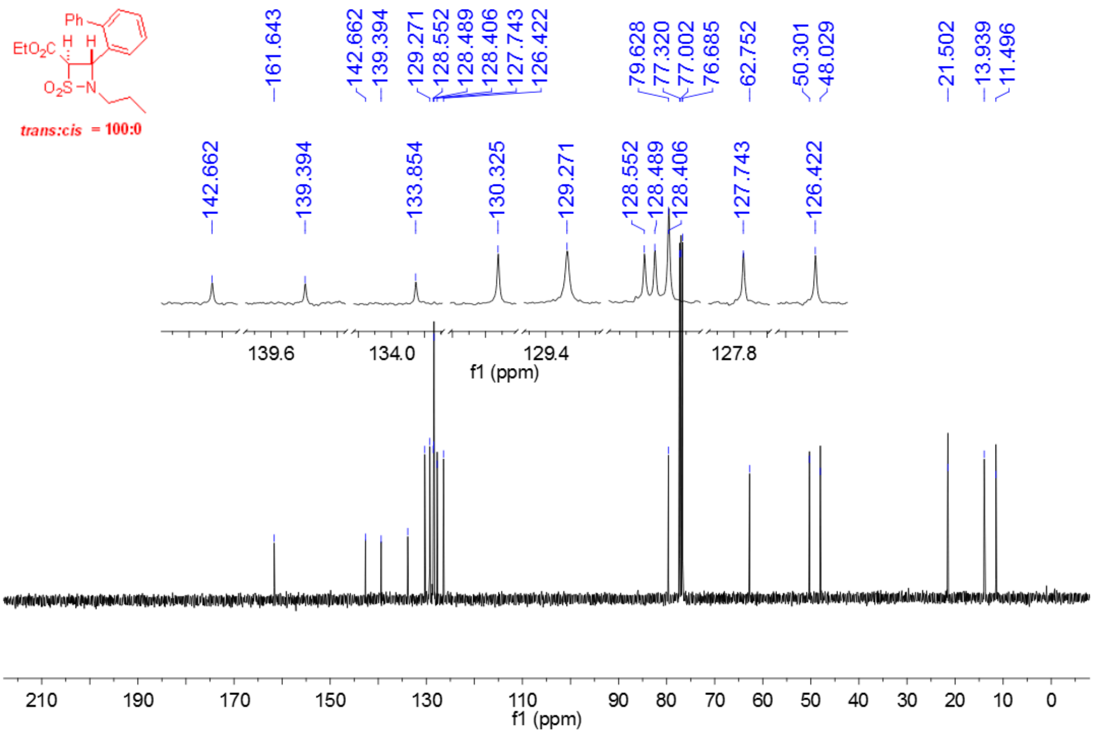

Ethyl trans-3-(naphthalen-2-yl)-2-propyl-1,2-thiazetidine-4-carboxylate 1,1-dioxide **(3o)**

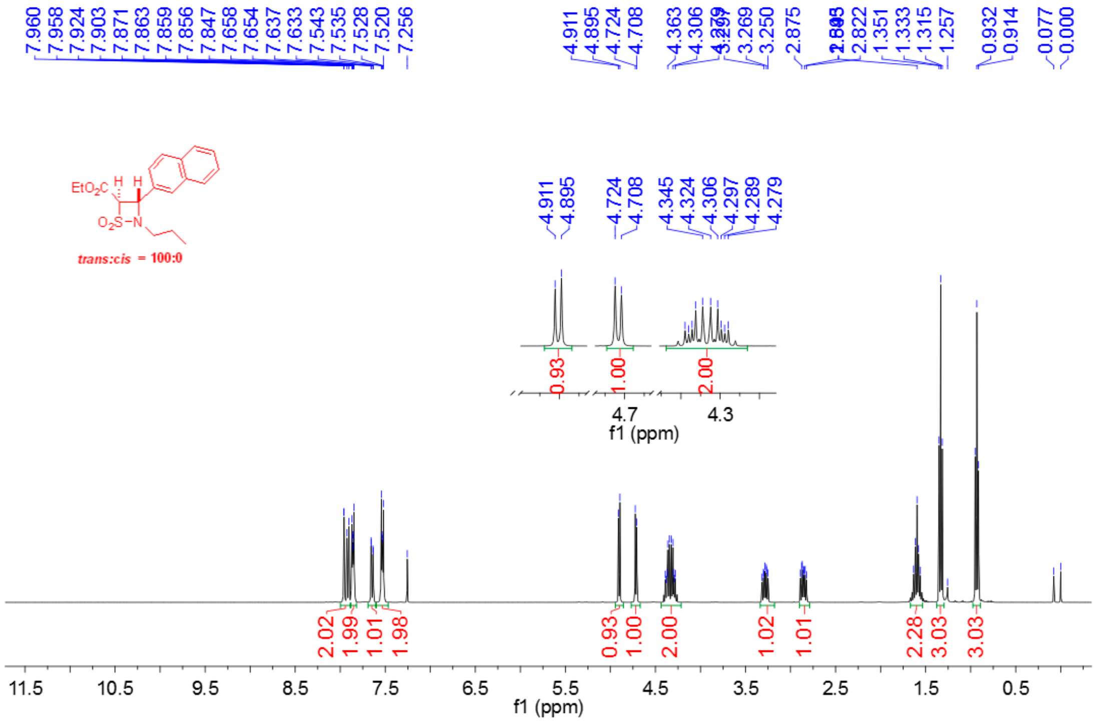

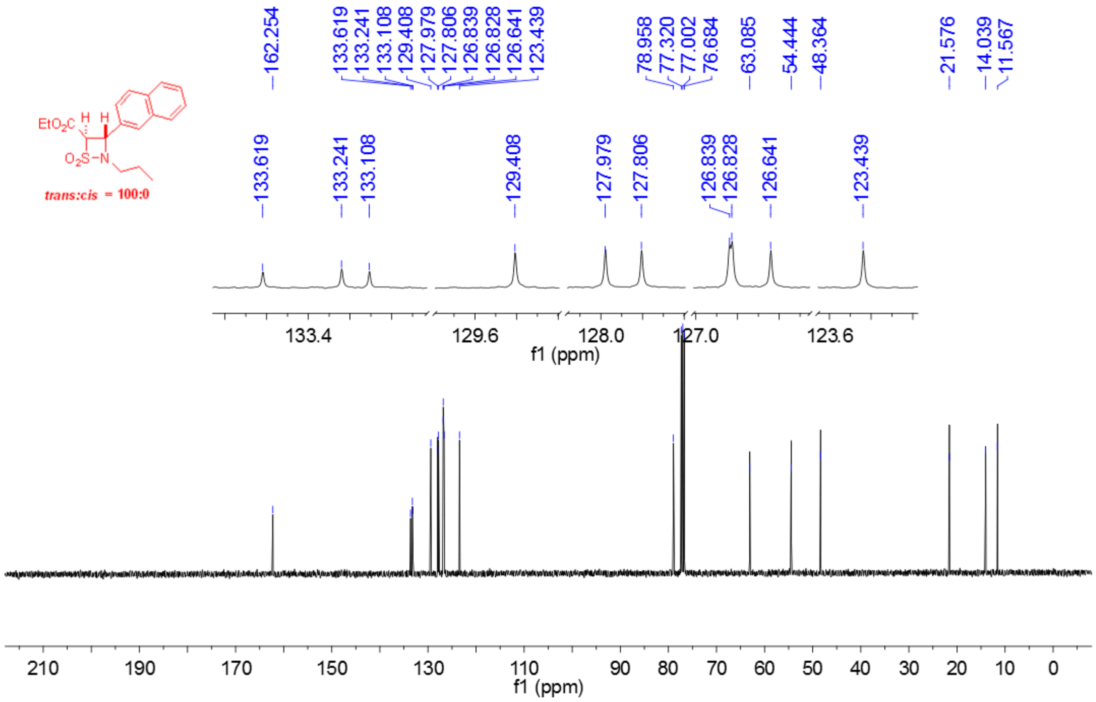

Ethyl trans-3-(2,4-dinitrophenyl)-2-propyl-1,2-thiazetidine-4-carboxylate 1,1-dioxide **(3p)**

Compounds *trans*-**3o** and *cis*-**5o** were isolated as a mixture, with a *trans/cis* ratio of 75:25.

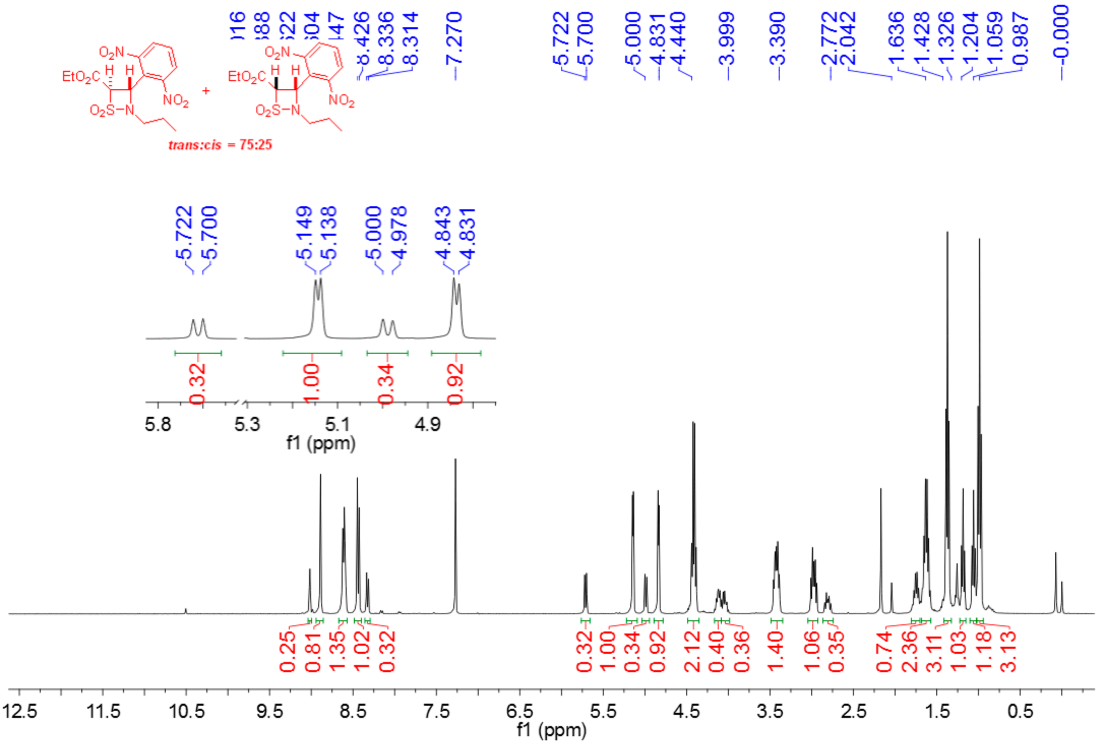

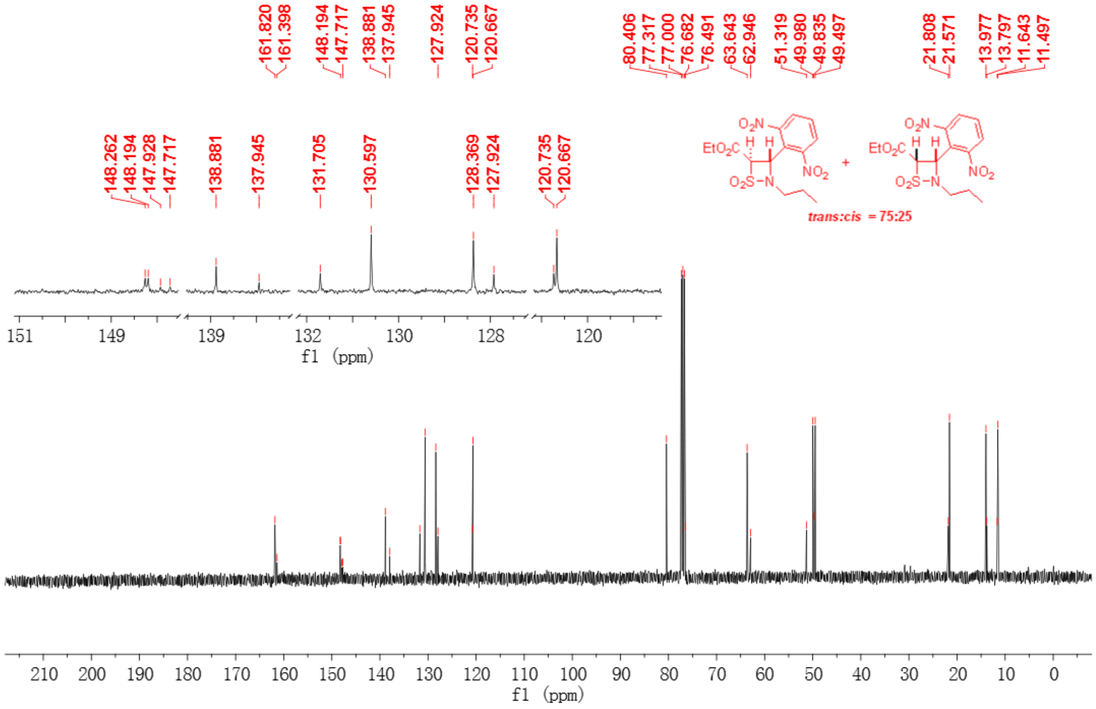

Ethyl trans- and cis-3-(5-methoxy-2-nitrophenyl)-2-propyl-1,2-thiazetidine-4-carboxylate 1,1-dioxide **(3q)**

Compounds *trans*-**3q** and *cis*-**5q** were isolated as a mixture, with a *trans/cis* ratio of 75:25.

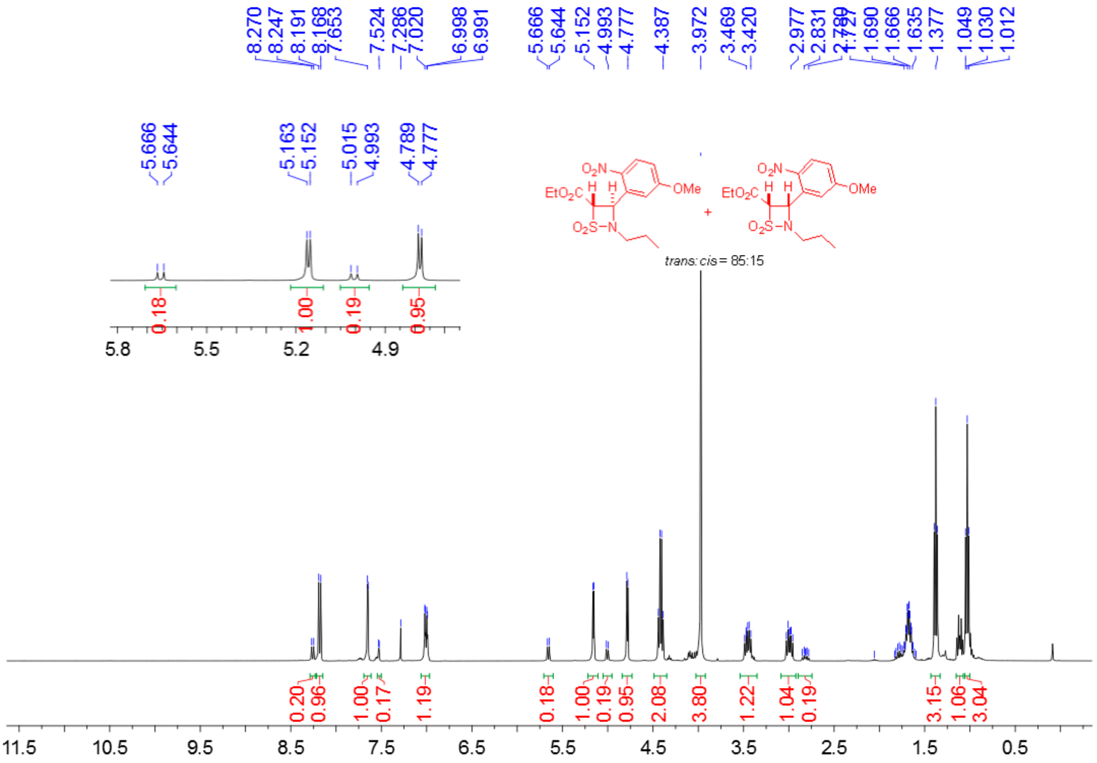

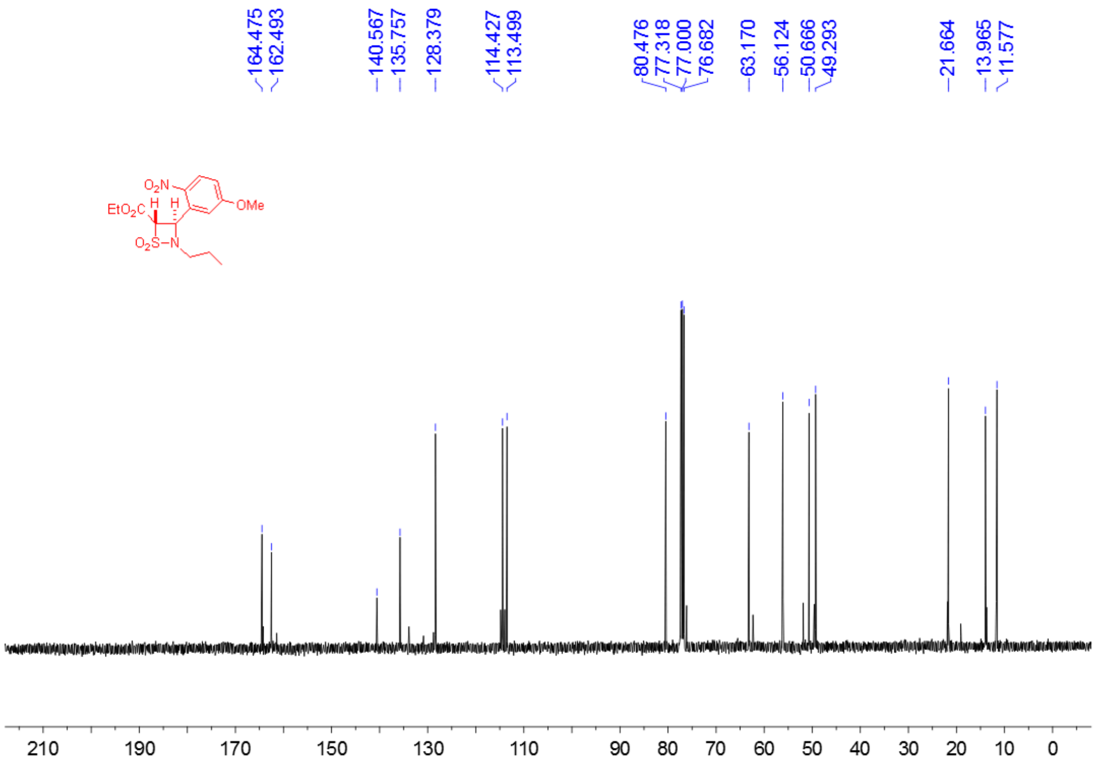

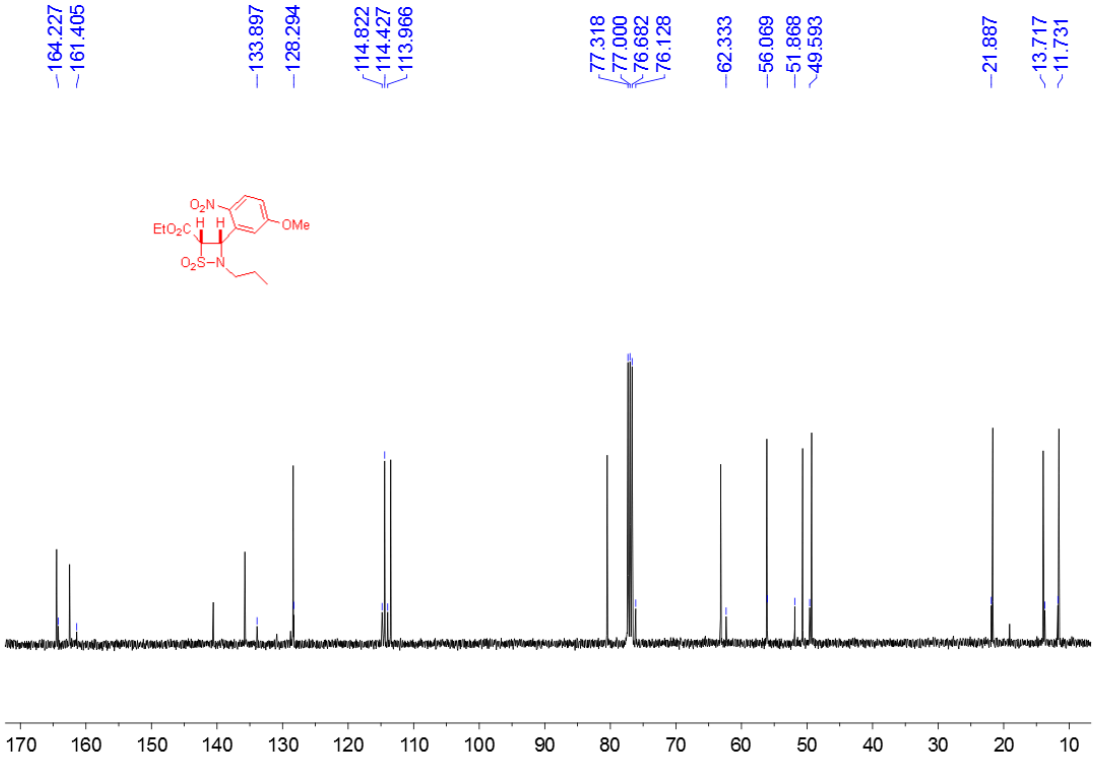
164.227
161.405
133.897
128.294
114.822
114.427
113.966
77.318
77.000
76.682
76.128
62.333
56.069
51.868
49.593
21.887
13.717
11.731
O_2N
OMe
EtO_2C
H
H
O_2S
N
170 160 150 140 130 120 110 100 90 80 70 60 50 40 30 20 10

Ethyl trans-4-(2-nitrophenyl)-1-propylazetidin-2-one-3-carboxylate **(4d)**

Compounds *trans*-**4d** and *cis*-**6d** were isolated as a mixture, with a *trans/cis* ratio of 90:10.

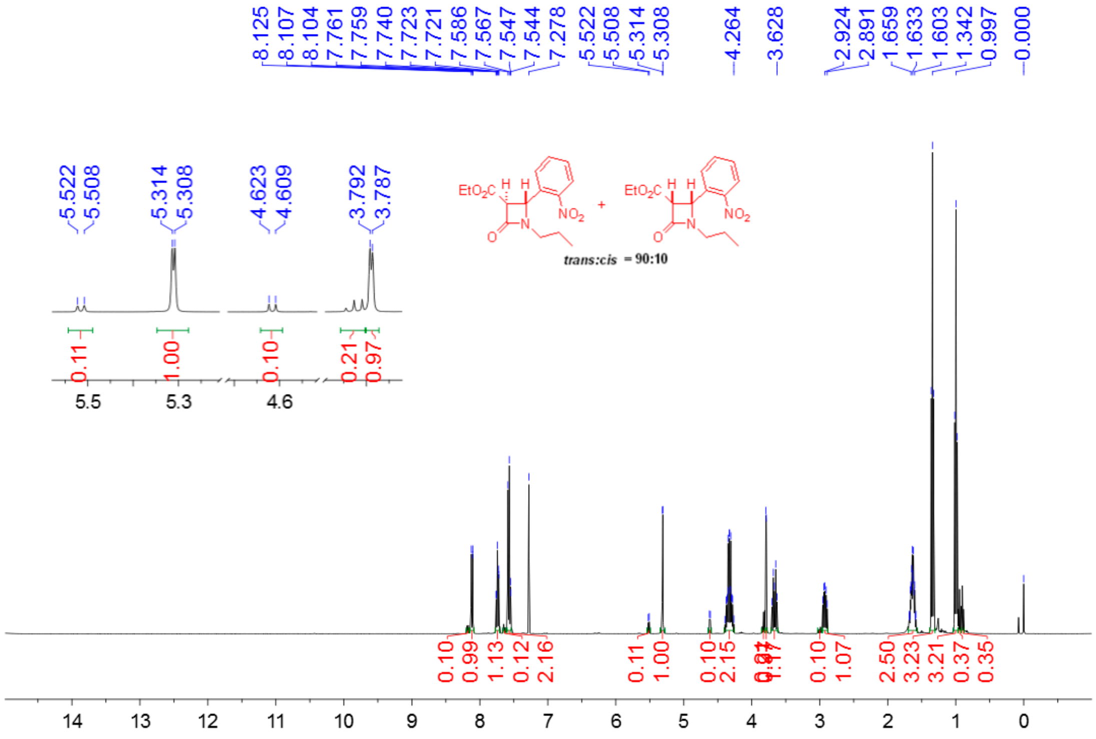

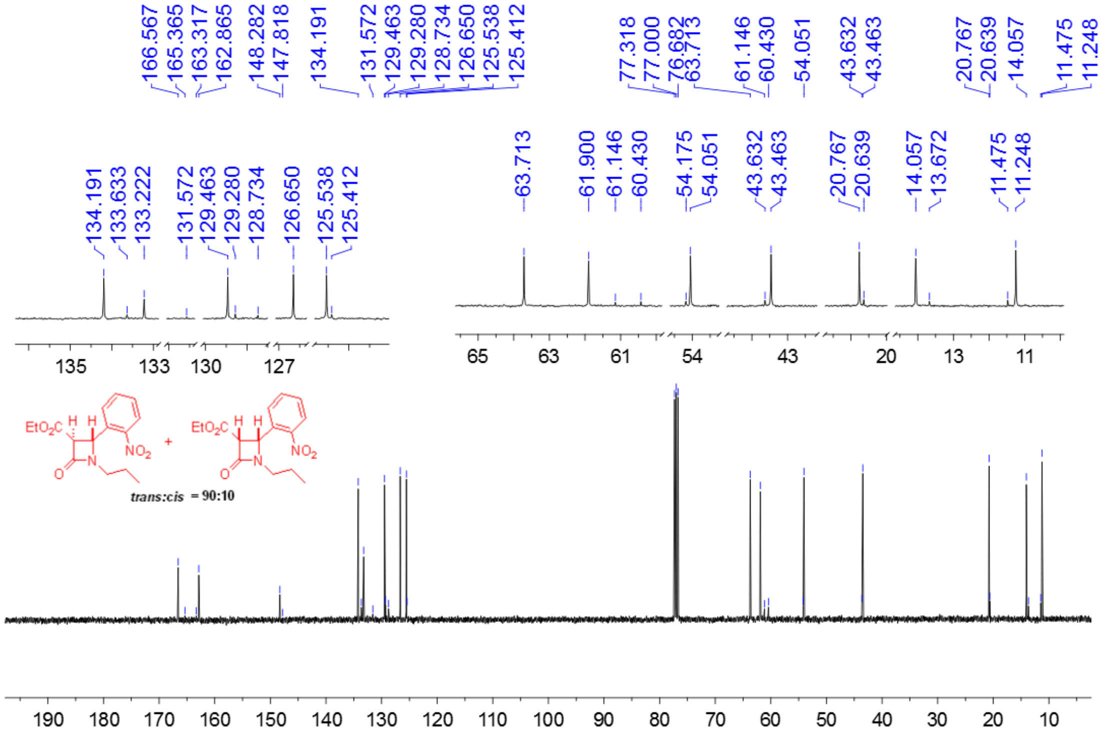

Ethyl trans-4-(3-nitrophenyl)-1-propylazetidin-2-one-3-carboxylate **(4r)**

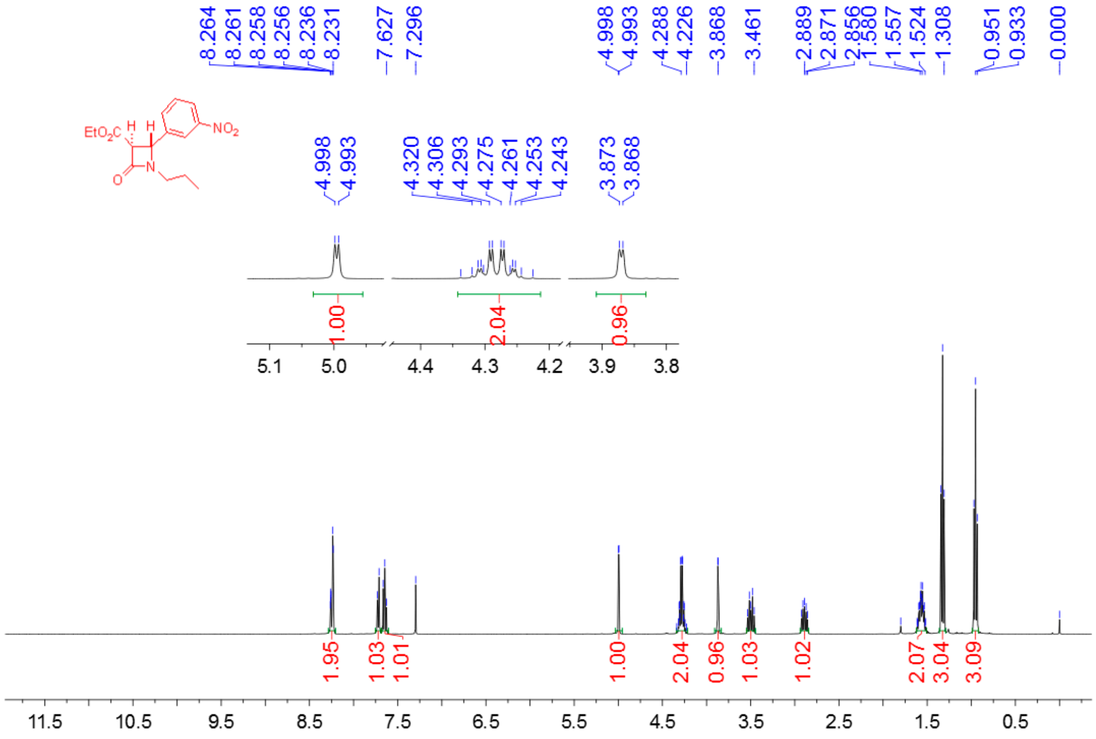

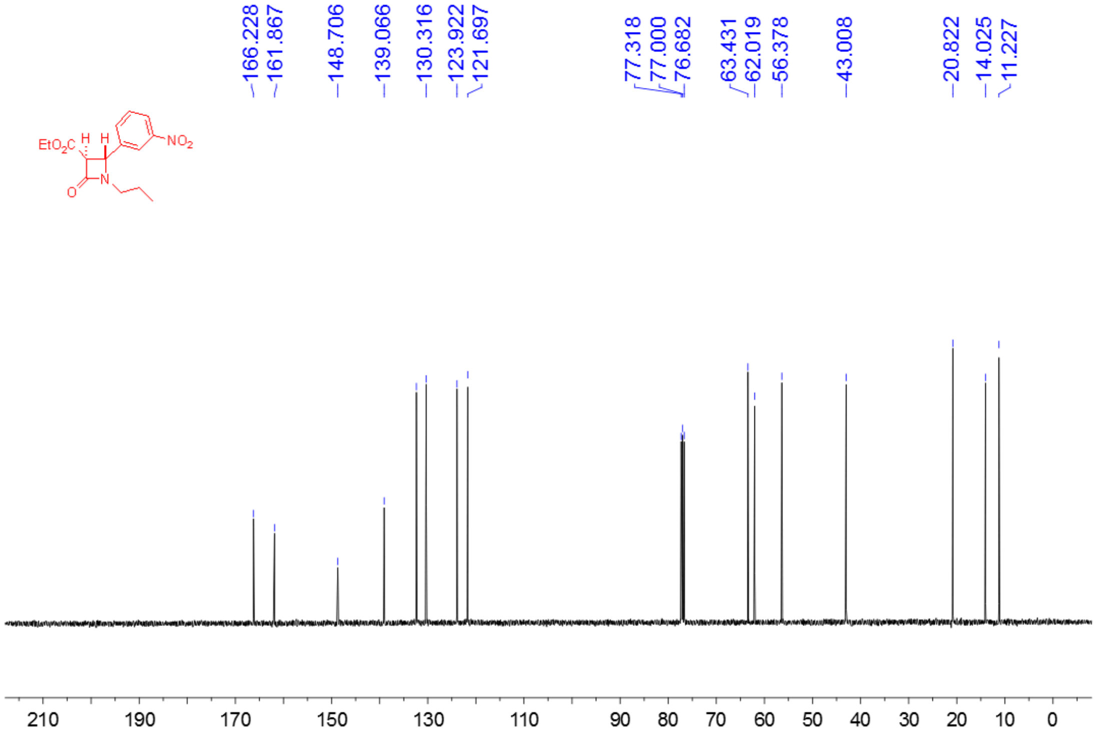

Ethyl trans-1-methyl-2-(2-nitrophenyl)-4-oxoazetidine-3-carboxylate **(4a)**

Compounds *trans*-**4a** and *cis*-**6a** were isolated as a mixture, with a *trans/cis* ratio of 85:15.

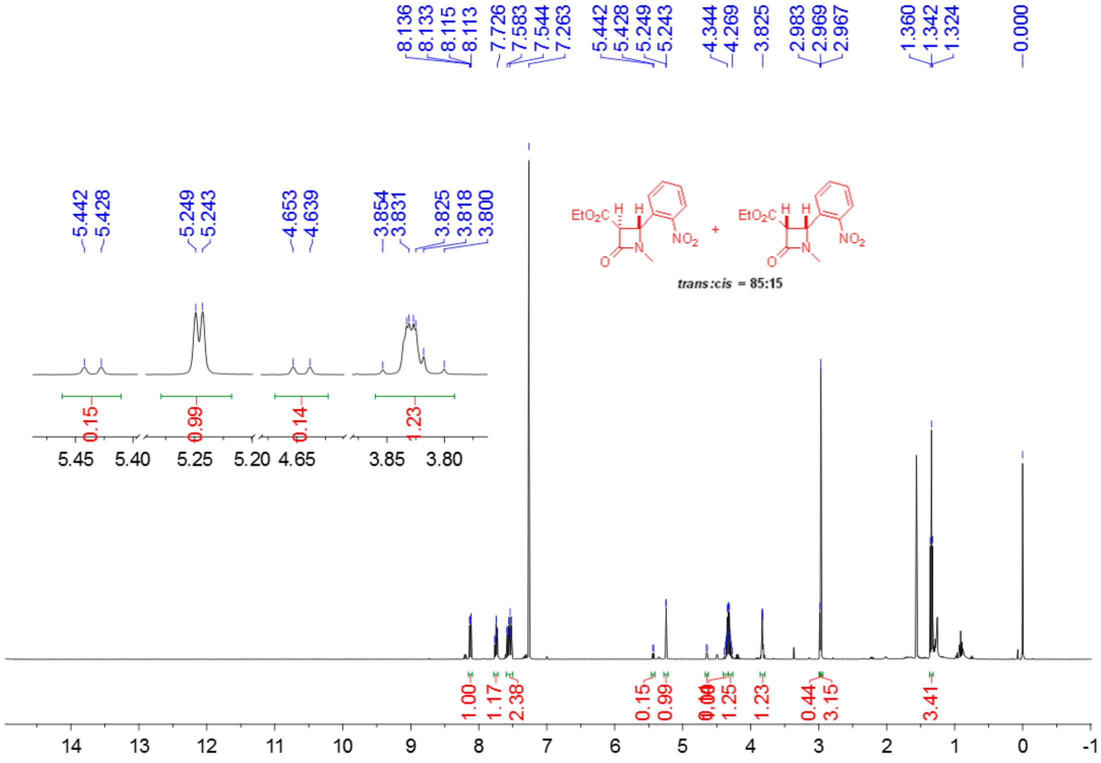

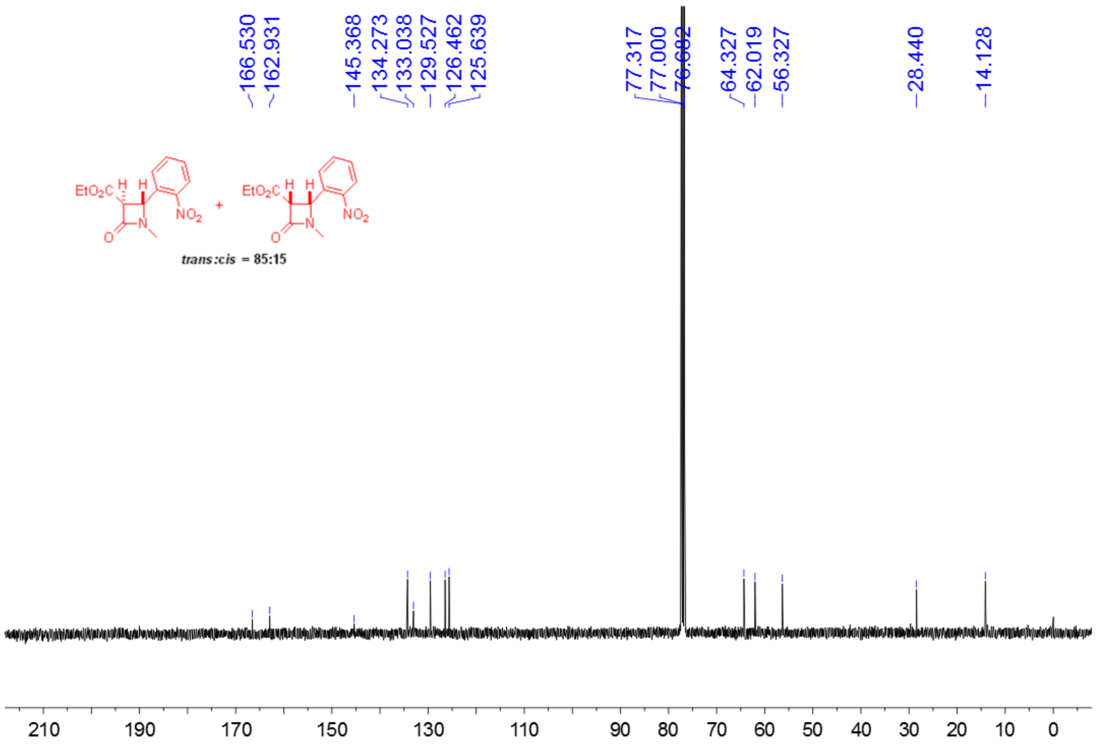

Ethyl trans-1-benzyl-2-(2-nitrophenyl)-4-oxoazetidine-3-carboxylate (**4e**)

Compounds *trans*-**4e** and *cis*-**6e** were isolated as a mixture, with a *trans/cis* ratio of 95:5.

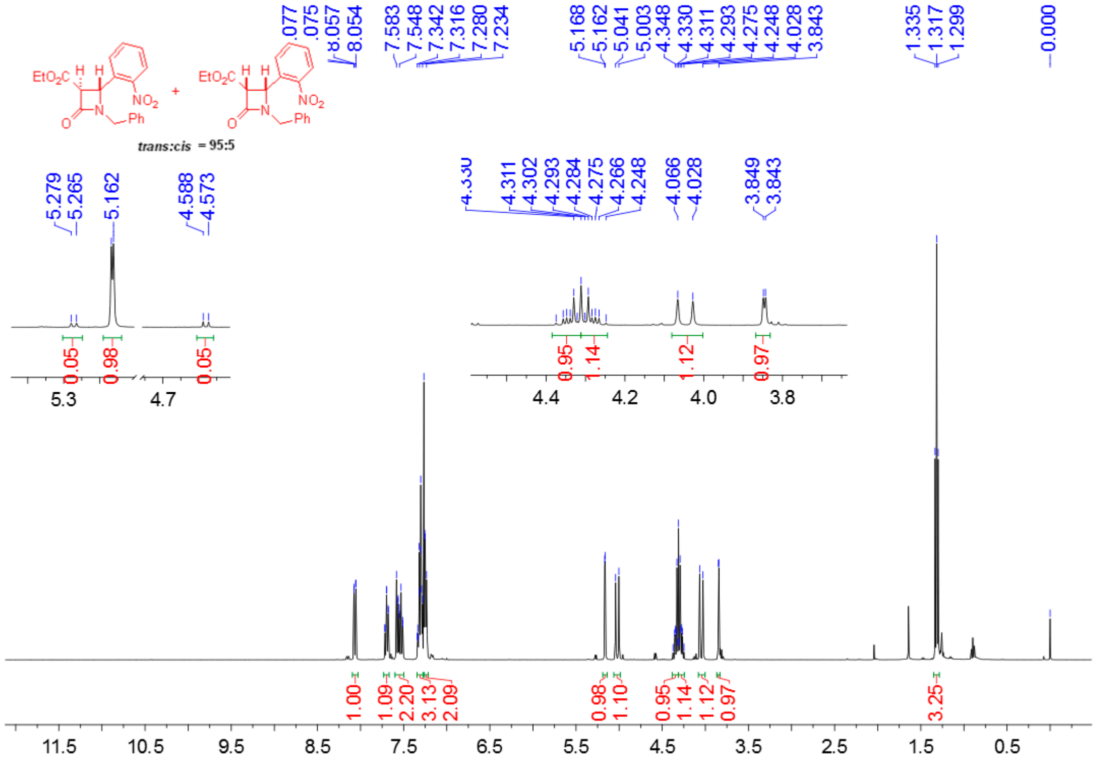

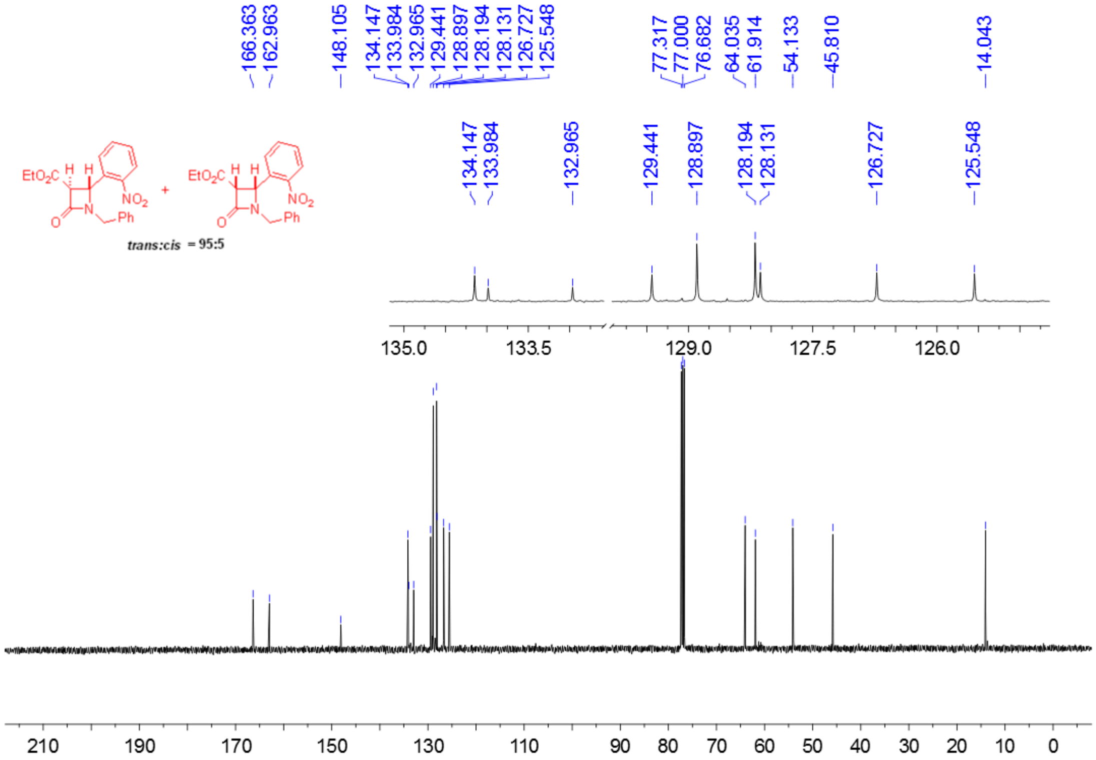

Ethyl trans-1-(tert-butyl)-2-(2-nitrophenyl)-4-oxoazetidine-3-carboxylate **(4f)**

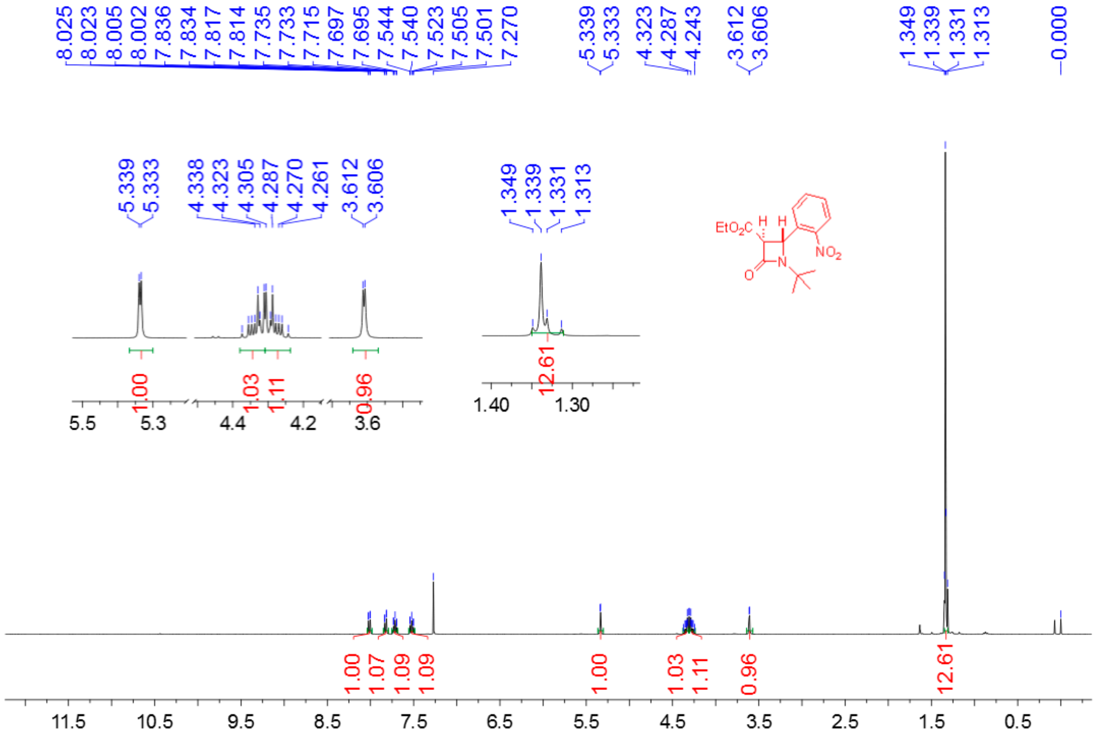

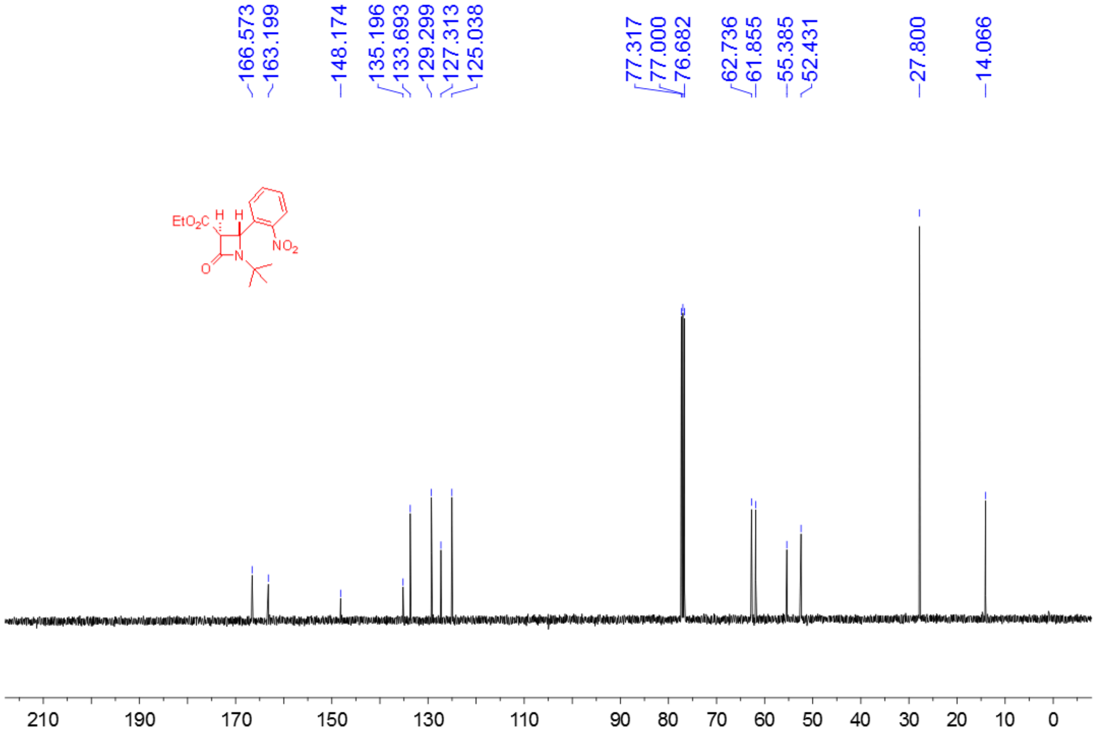

Ethyl cis-1-(tert-butyl)-2-(2-nitrophenyl)-4-oxoazetidine-3-carboxylate **(6f)**

This compound was isolated as a mixture with EtO_2CCH_2COOH; however, further purification of the mixture by treating the column chromatography with Et_3N led to isomerization of *cis*-**6f** into *trans*-**4f**. Thus, herein we present ^{1}H and ^{13}C NMR spectra of the mixture.

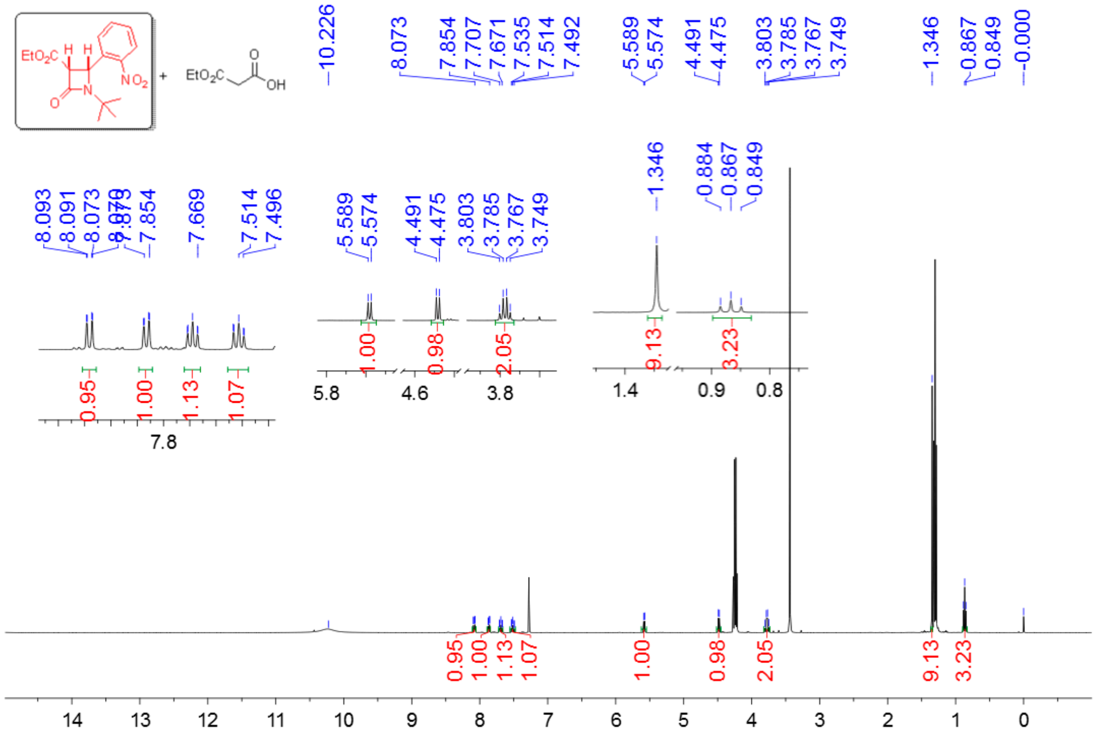

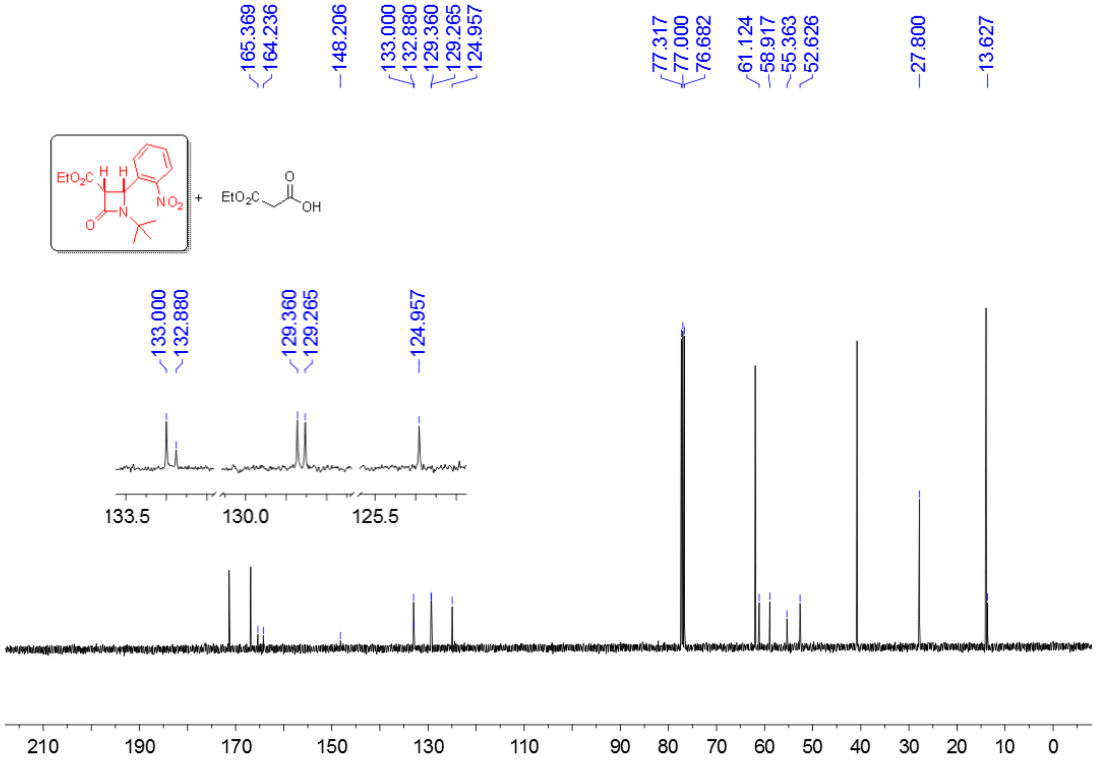

2. Copies of representative ^{1}H spectra of reaction mixtures.

Note: Most of *cis*- and *trans*-products involving the *ortho*-nitro effect were inseparable, as indicated in the experimental section. For sulfa-Staudinger reactions, determining the *trans/cis* ratios by the isolated mixtures or crude reaction mixtures gave the same results. For Staudinger reactions, the ratios were determined by their ^{1}H NMR spectra of the crude reaction mixtures, because Et_3N added in the purification step by column chromatography led to the isomerization of *cis*-products into *trans*-products. Herein, only some of spectra of crude reaction mixtures are given.

Table 1, entry 10.

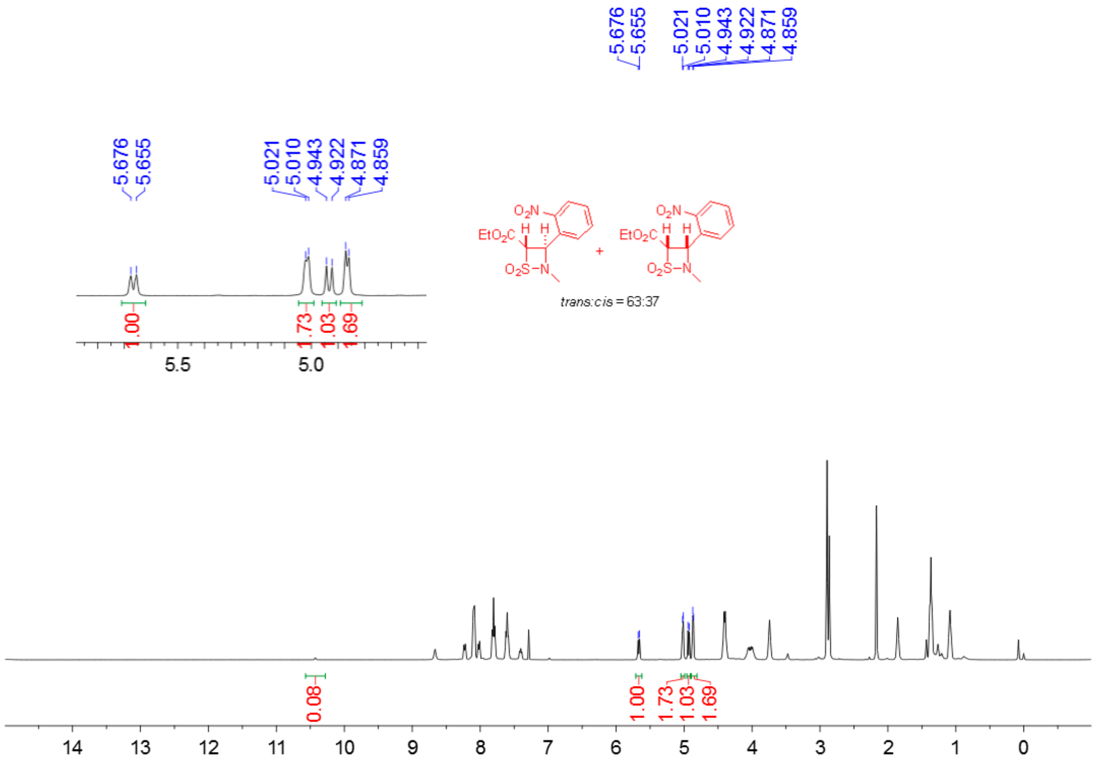

Table 1, entry 11

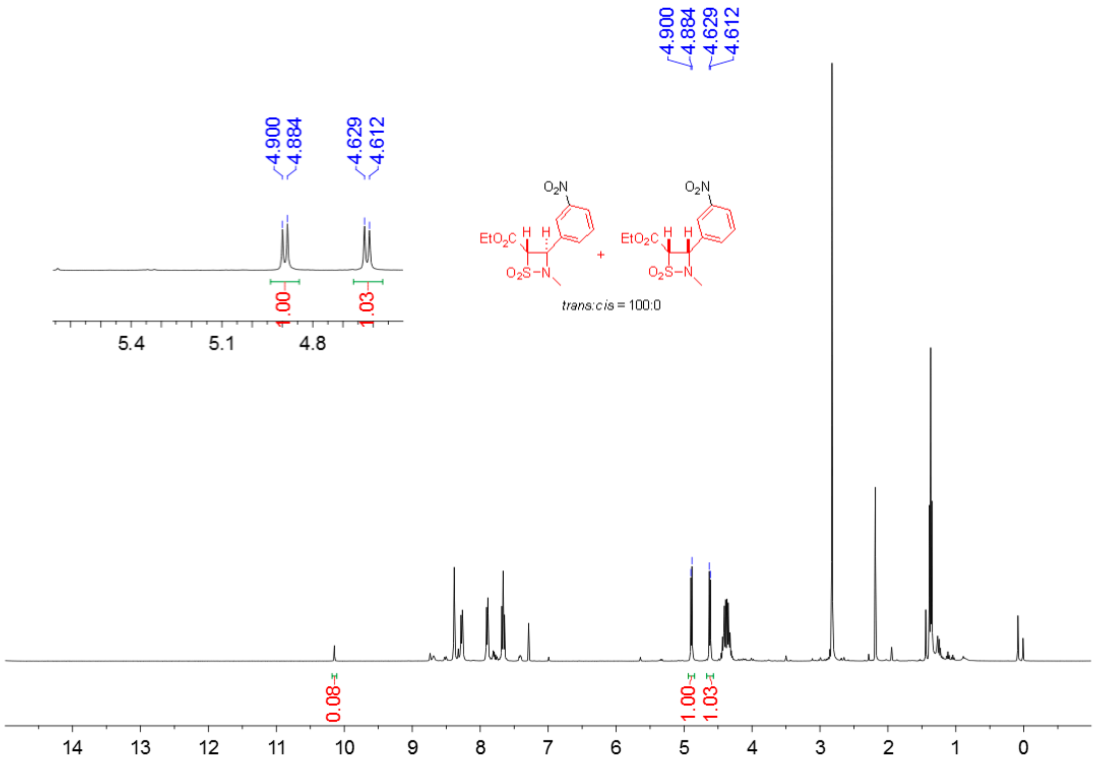

Table 1, entry 12

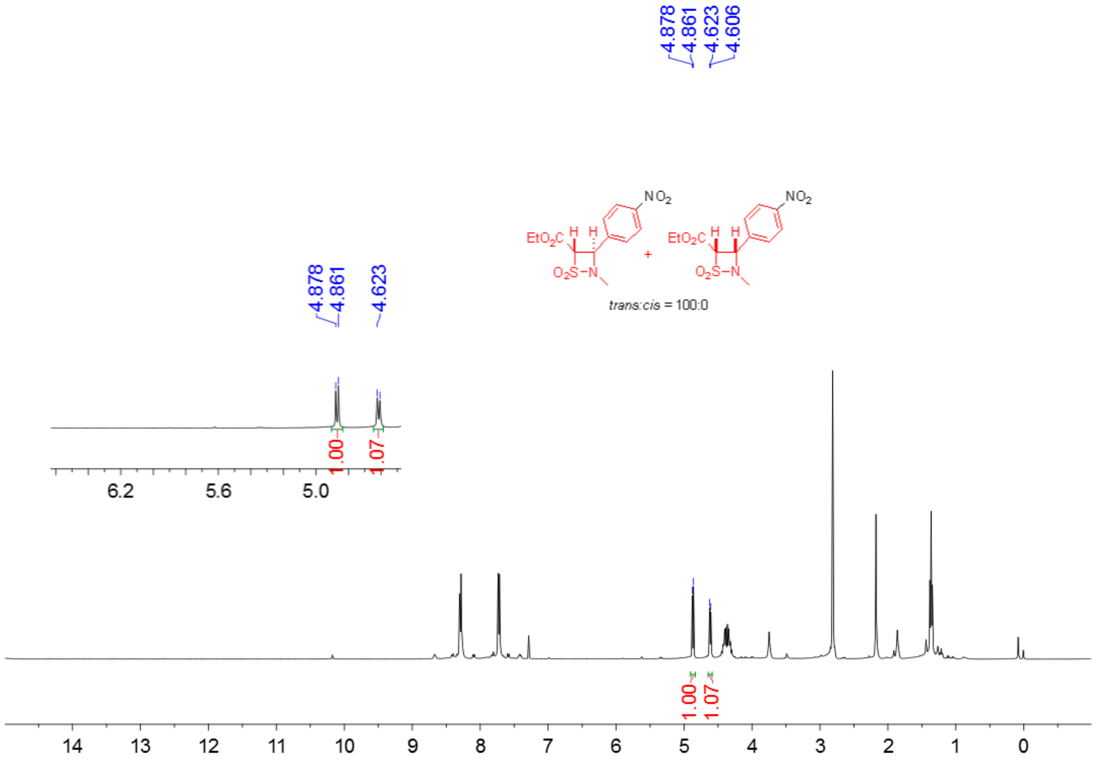

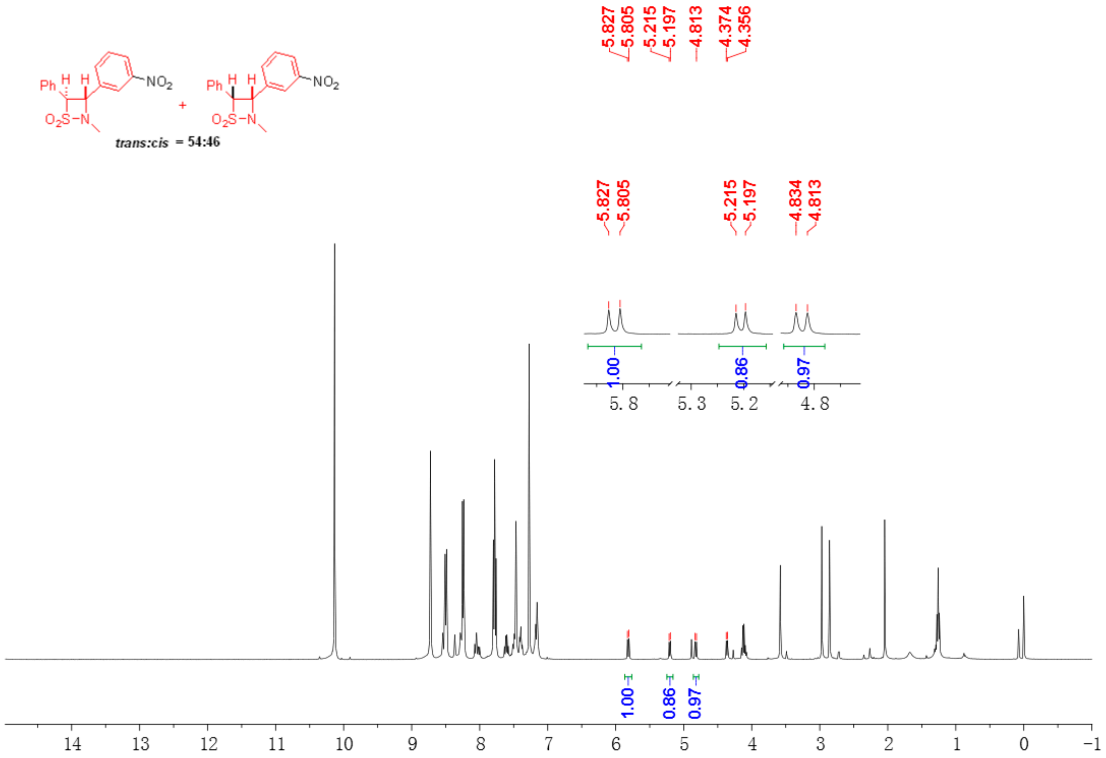

Chemical shifts 6.53 and 5.87 ppm were not the characteristic peaks of *cis*-products, based on the following facts: (a) the coupling constant of 10.4 Hz is too great; (b) *cis*-product was not isolated.

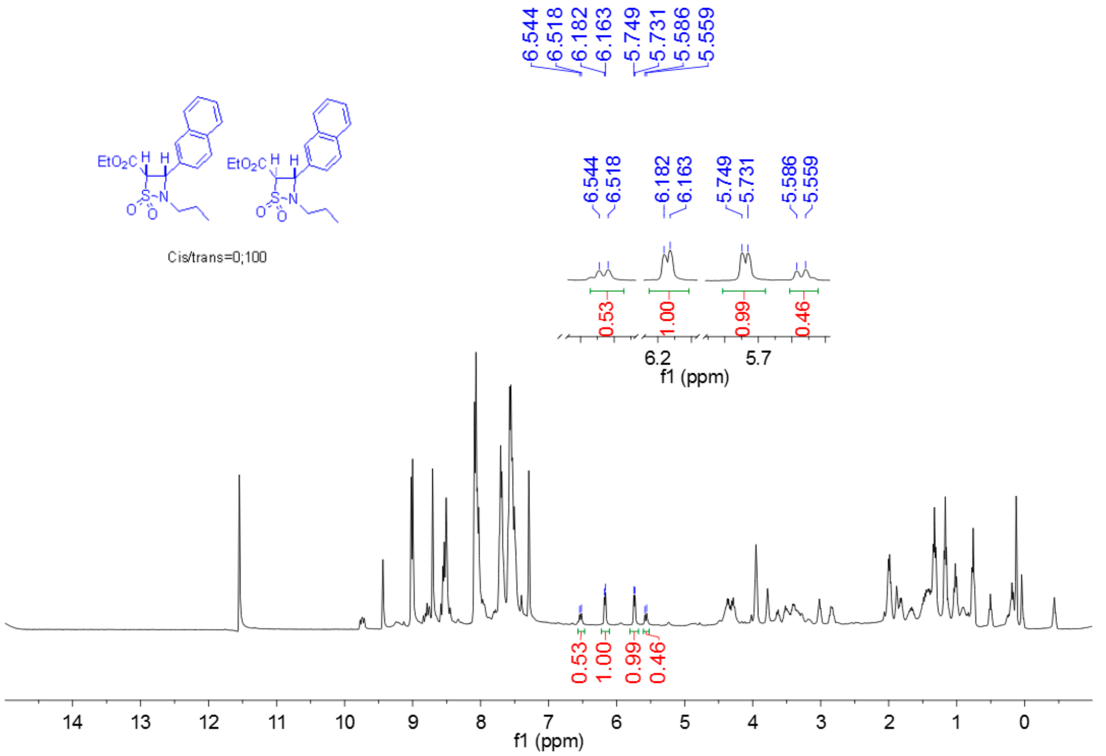

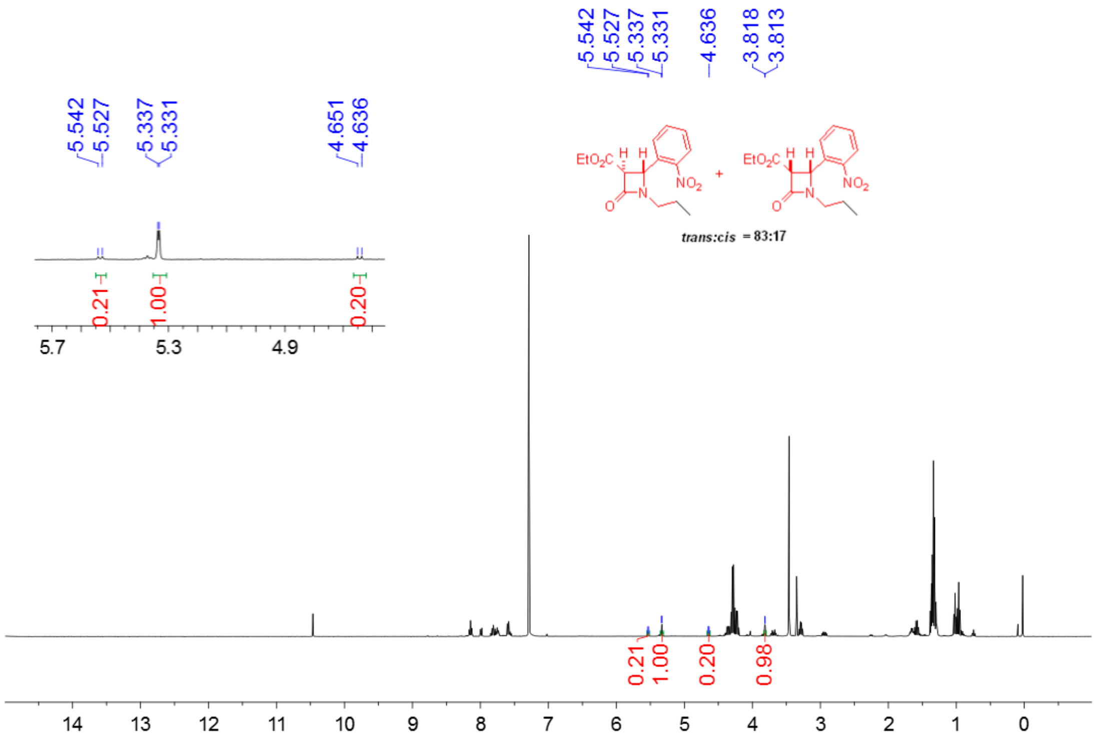
5.542
5.527
5.337
5.331
4.636
3.818
3.813
5.542
5.527
5.337
5.331
4.651
4.636
0.21
1.00
0.20
5.7
5.3
4.9
EtO2C
H
H
NO2
N
O
+
trans:cis = 83:17
0.21
1.00
0.20
0.98
14
13
12
11
10
9
8
7
6
5
4
3
2
1
0

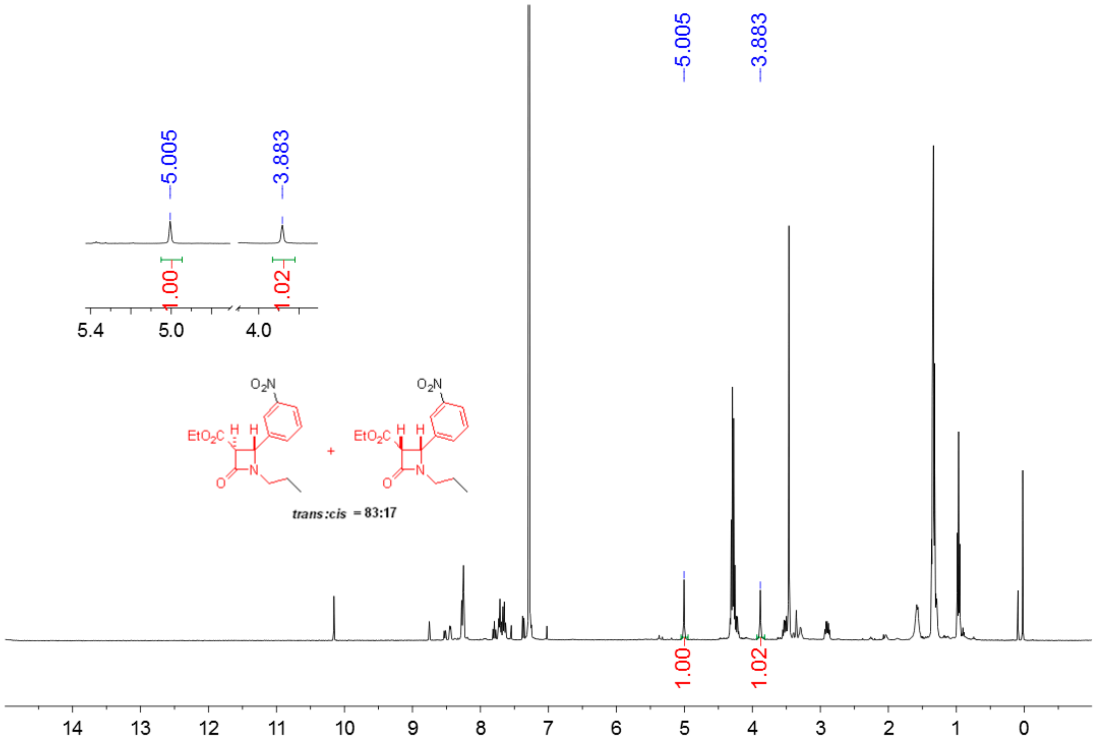
5.005
3.883
5.005
3.883
1.00
1.02
5.4
5.0
4.0
O2N
EtO2C
H
H
N
O
+
trans:cis = 83:17
1.00
1.02
14
13
12
11
10
9
8
7
6
5
4
3
2
1
0

Trans/cis = 100:0. Dimethyl malonate (6.27 and 3.85 ppm) was added as an internal standard to determine the yield (59%).

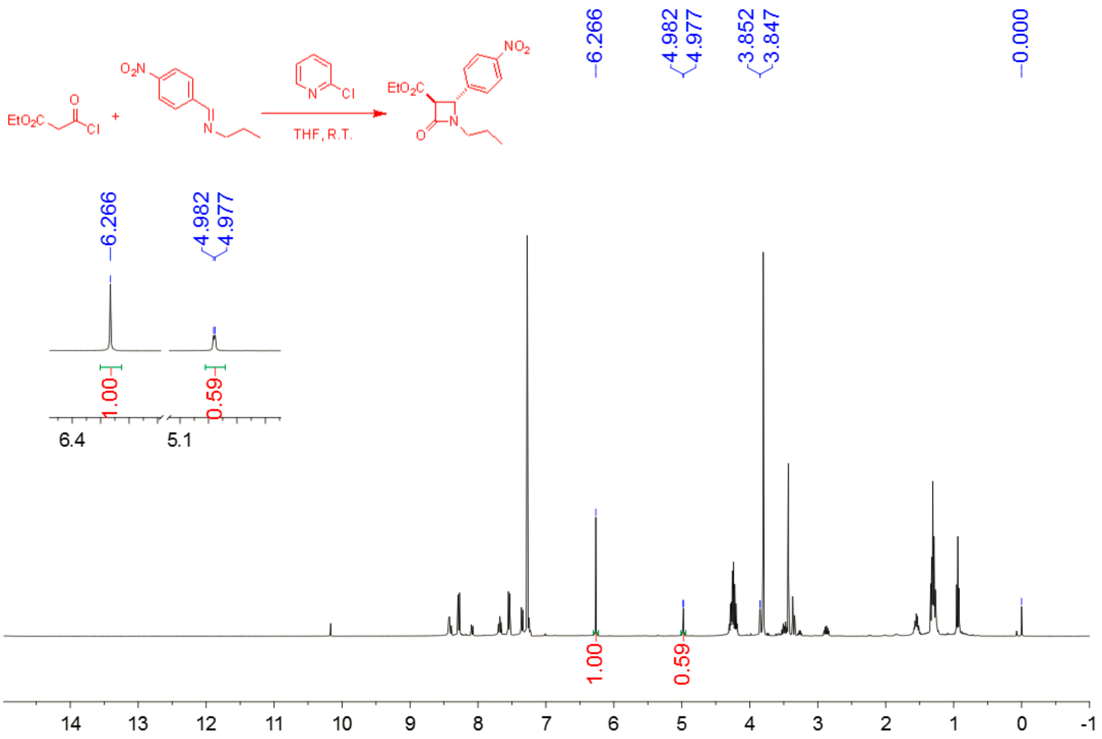

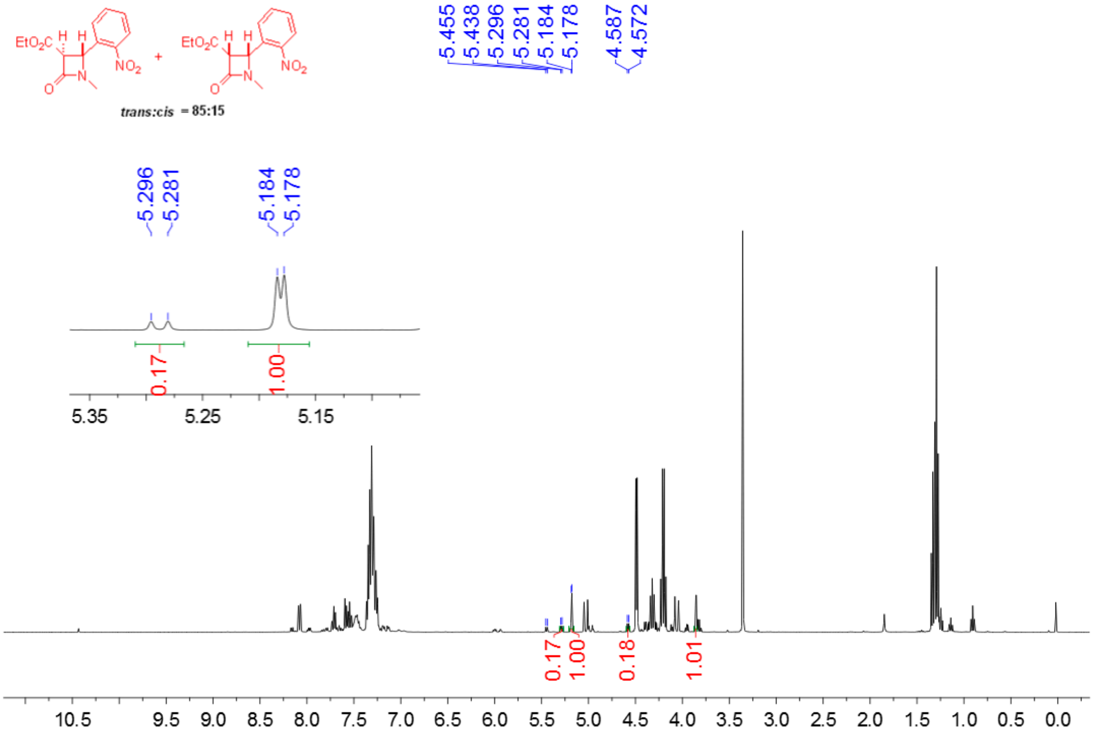

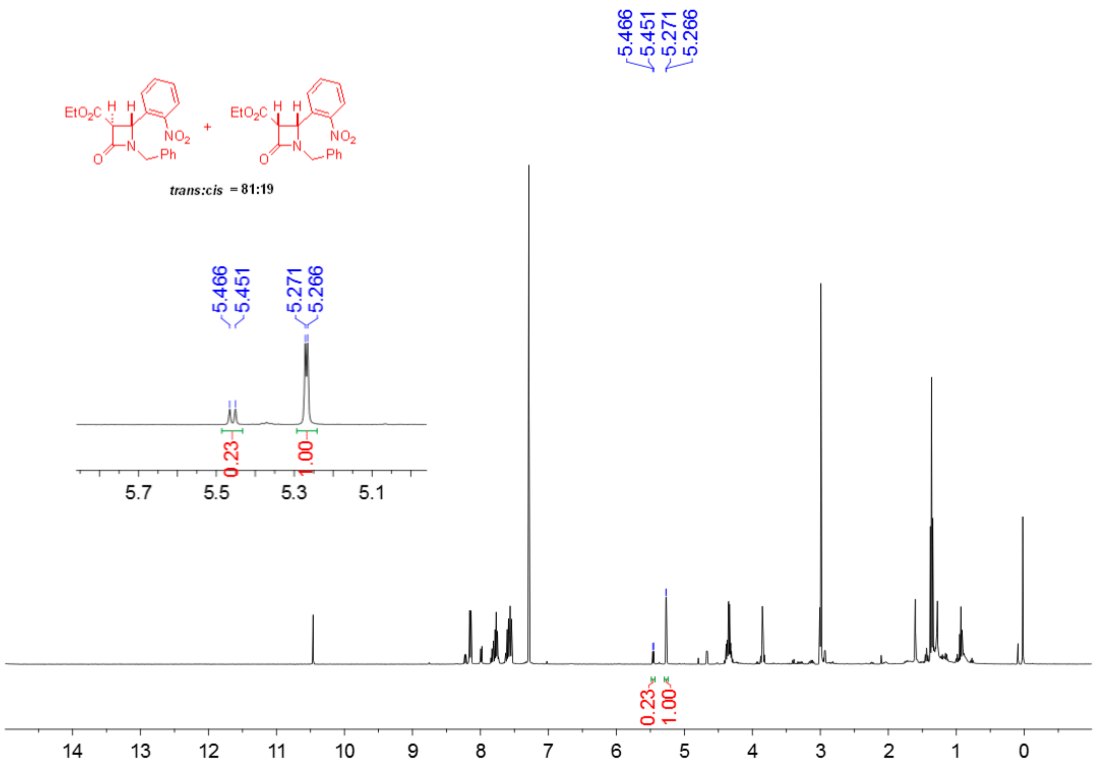

5.466
5.451
5.271
5.266
EtO2C H H
NO2
N
Ph
O
+
trans:cis = 81:19
0.23
1.00
5.7
5.5
5.3
5.1
14
13
12
11
10
9
8
7
6
5
4
3
2
1
0

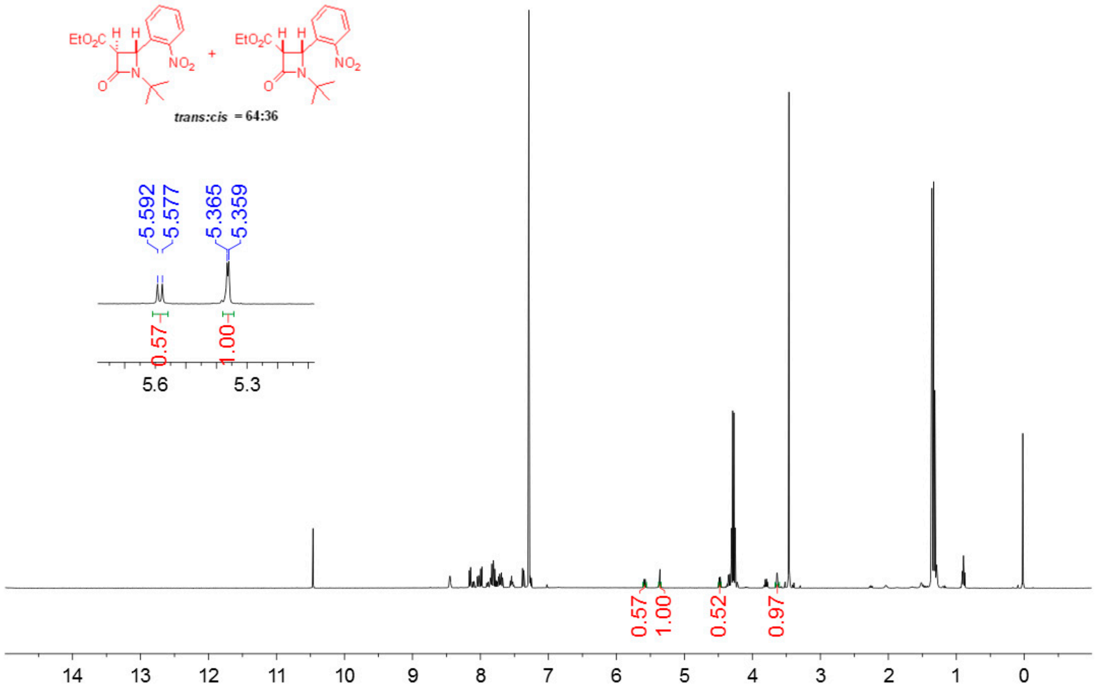

EtO2C H H
NO2
N
O
+
trans:cis = 64:36
5.592
5.577
5.365
5.359
0.57
1.00
5.6
5.3
0.52
0.97
14
13
12
11
10
9
8
7
6
5
4
3
2
1
0
